# Design, Synthesis, Biological Evaluation and In Silico Study of Benzyloxybenzaldehyde Derivatives as Selective ALDH1A3 Inhibitors

**DOI:** 10.3390/molecules26195770

**Published:** 2021-09-23

**Authors:** Ali I. M. Ibrahim, Balqis Ikhmais, Elisabet Batlle, Waed K. AbuHarb, Vibhu Jha, Khaled T. Jaradat, Rafael Jiménez, Raquel Pequerul, Xavier Parés, Jaume Farrés, Klaus Pors

**Affiliations:** 1 Faculty of Pharmacy, Al-Zaytoonah University of Jordan, Amman 11733, Jordan; b.ikhmais@zuj.edu.jo (B.I.); waedkhalil1997@gmail.com (W.K.A.); 2 Faculty of Life Sciences, Institute of Cancer Therapeutics, School of Pharmacy and Medical Sciences, University of Bradford, Bradford BD7 1DP, UK; e.batllerocafort@bradford.ac.uk; 3 Department of Biochemistry and Molecular Biology, Faculty of Biosciences, Universitat Autònoma de Barcelona, E-08193 Barcelona, Spain; rafael.jimenez@uab.cat (R.J.); raquel.pequerul@gmail.com (R.P.); xavier.pares@uab.cat (X.P.); jaume.farres@uab.cat (J.F.); 4 Department of Pharmacy, University of Pisa, 56126 Pisa, Italy; vibhujha16@gmail.com; 5 College of Engineering and Technology, American University of the Middle East, Kuwait City 54200, Kuwait; khaled.jaradat@aum.edu.kw

**Keywords:** aldehyde dehydrogenase, ALDH1A1, ALDH1A3, ALDH3A1, A549 cells, H1299 cells, non-small cell lung cancer, cytotoxicity, in silico

## Abstract

Aldehyde dehydrogenase 1A3 (ALDH1A3) has recently gained attention from researchers in the cancer field. Several studies have reported ALDH1A3 overexpression in different cancer types, which has been found to correlate with poor treatment recovery. Therefore, finding selective inhibitors against ALDH1A3 could result in new treatment options for cancer treatment. In this study, ALDH1A3-selective candidates were designed based on the physiological substrate resemblance, synthesized and investigated for ALDH1A1, ALDH1A3 and ALDH3A1 selectivity and cytotoxicity using ALDH-positive A549 and ALDH-negative H1299 cells. Two compounds (ABMM-15 and ABMM-16), with a benzyloxybenzaldehyde scaffold, were found to be the most potent and selective inhibitors for ALDH1A3, with IC_50_ values of 0.23 and 1.29 µM, respectively. The results also show no significant cytotoxicity for ABMM-15 and ABMM-16 on either cell line. However, a few other candidates (ABMM-6, ABMM-24, ABMM-32) showed considerable cytotoxicity on H1299 cells, when compared to A549 cells, with IC_50_ values of 14.0, 13.7 and 13.0 µM, respectively. The computational study supported the experimental results and suggested a good binding for ABMM-15 and ABMM-16 to the ALDH1A3 isoform. From the obtained results, it can be concluded that benzyloxybenzaldehyde might be considered a promising scaffold for further drug discovery aimed at exploiting ALDH1A3 for therapeutic intervention.

## 1. Introduction

The aldehyde dehydrogenase (ALDH) superfamily is a group of NAD(P)^+^-dependent enzymes comprised by 19 different human isoforms widely distributed and diversely expressed in the body tissues [1]. The main function of these enzymes is to catalyze the oxidation of both endogenous and exogenous aldehydes to their corresponding carboxylic acids [1]. Several ALDH isoforms, mainly ALDH1A isoforms, are involved in the biosynthetic oxidation of retinaldehyde to retinoic acid (RA), the physiologically active metabolite of vitamin A [2,3]. RA is a well-known regulator of cell-signaling pathways during embryonic development, which acts through binding to the nuclear retinoic acid receptors (RARs and RXRs), and has been found to be involved in the diverse modulatory roles of the differentiation and proliferation of normal cells by modulating gene transcription [4,5]. In cancer, it has been hypothesized that the replicating cancer cells are derived from a small subpopulation of cancer stem cells (CSCs) or tumor-initiating cells, in which RA-mediated signaling pathways are essential for their proliferation and differentiation [6,7,8]. The importance of identifying ALDH activity during CSC differentiation and cancer initiation has been extensively reviewed and remarked [9,10,11].

Different isoforms of the ALDH1 subfamily have been commonly described as biomarkers for CSCs in several cancer cell types [12,13], where both ALDH1A1 and ALDH1A3 are highly expressed in stem-cell-like subpopulations of different cancer types [14,15]. In particular, ALDH1A3 targeting has been investigated for its potential clinical applications in several cancer types, including metastatic breast cancer [16], non-small-cell lung cancer (NSCLC) [17] and mesenchymal subtype gliomas [18]. In addition, ALDH1A3 expression has been correlated with poor prognosis in breast cancer [19], chemoresistance of mesothelioma [20] and radioresistance of head and neck cancer [21], and its inhibition has been proven to induce the cytotoxicity of those cancer cells that present an ALDH1A3 overexpression [19,22]. Moreover, ALDH1A3 downregulation has been found to reduce CD44 and epithelium-specific antigen, as well as CSCs isolated from breast cancer [23].

Targeting ALDHs through the analysis of their catalytic centers has been implemented [24,25,26]. Although ALDH1A3 is a tetramer, each monomer can work independently, with a NAD(P)^+^-binding pocket and a substrate-binding pocket. The latter is occupied by a strictly conserved catalytic cysteine residue (Cys314 in ALDH1A3 or Cys303 in ALDH1A1 according to original numbering [27]), which is essential for the active-site nucleophilic attack. Alongside, there are several aromatic amino acid residues, such as Trp189 or Tyr472 (ALDH1A3 numbering), all contributing to the substrate-binding pocket [28,29,30]. An in-depth knowledge of the isoform-specific ALDH active sites has been found helpful in drug discovery, not only in the design of compounds based on the substrate-binding pocket architecture, but also in calculating and predicting the affinity and binding interactions of substrates and inhibitors [31].

The modulation of ALDH isoforms with small molecules has been a matter of interest in recent years [32,33,34]. A well-known and widely used compound is diethylamino benzaldehyde (DEAB), commonly exploited as a pan-inhibitor for ALDH enzymes, but it was found to have a variable modulating activity by either working as a substrate, an inhibitor or both [35]. The binding interaction between DEAB and ALDH7A1 active site has been identified, whereby the contribution of the benzaldehyde moiety of DEAB with the aromatic and nucleophilic amino acids of the catalytic center has been highlighted [26]. Nevertheless, DEAB remains a small molecule with limited functional groups that binds unselectively to ALDH enzymes; hence, the search for isoform-targeting selective molecules remains of interest. This is of particular interest for those diseases where a transient or permanent imbalance in isoform-selective ALDH function and/or expression occur. Moreover, having isoform-selective inhibitors could be a useful tool to probe ALDH expression and activity in a more accurate way.

**Figure 1:**
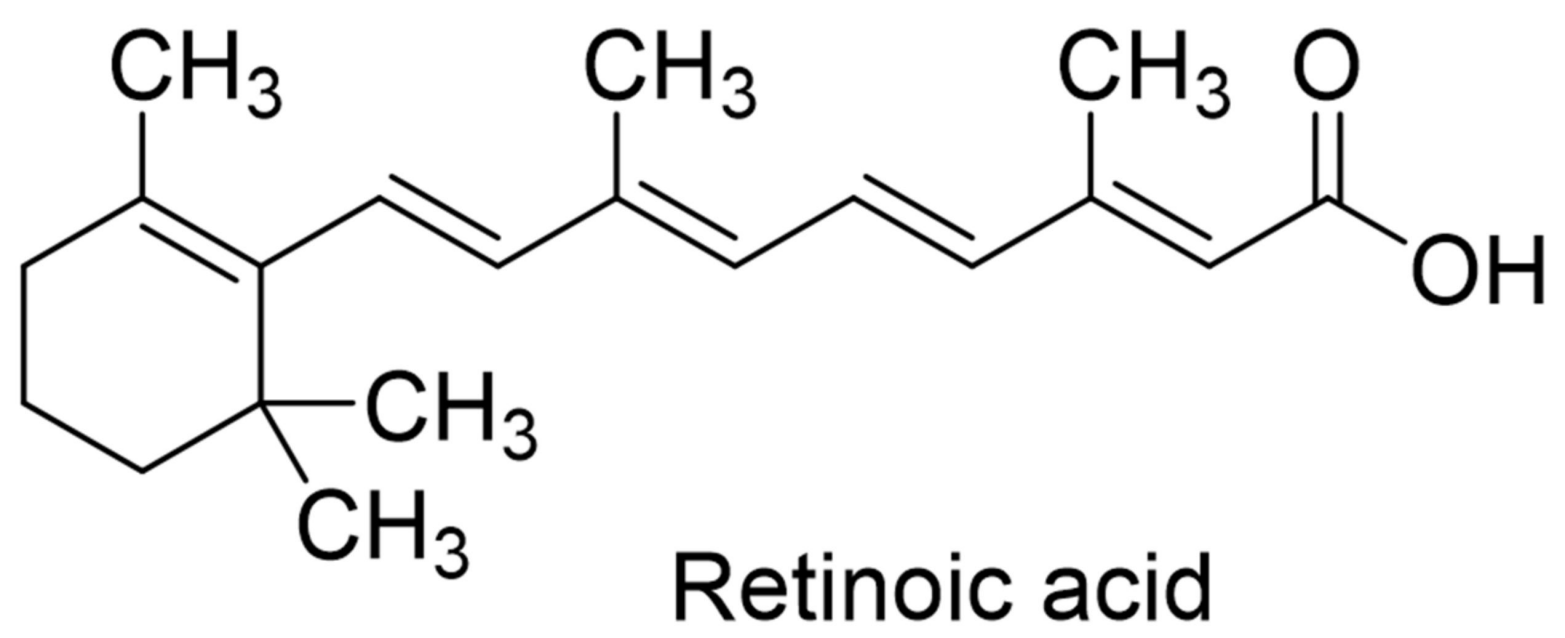
Chemical structure of retinoic acid.

The approach presented in the current study may slightly differ from the classical design of molecules for ALDH inhibition, as the compounds here have been strategically developed to be recognized by the enzyme as substrates and form a stable enzyme–ligand complex that causes an inhibition. Therefore, three main structural aspects were taken into account to design a set of compounds that could potentially target ALDH1A3: First, extending the benzaldehyde moiety by a benzene ring to enhance the affinity toward the enzyme active site. This enhancement was predicted due to the existence of aromatic amino acids at the enzyme active sites, which may potentiate the intermolecular binding interactions. Second, the addition of a double bond between the aldehyde and benzene ring, attempting to relatively mimic the conjugation of retinoic acid (structure shown in Figure 1). Finally, the replacement of the aldehyde by an alcohol in some compounds and a carboxylic acid in another, to (i) evaluate its importance for both enzyme activity and cytotoxicity, and (ii) assess the outcomes of its removal, since it is the group that provides the ability of the compounds to act as ALDH substrates.

**Scheme 1:**
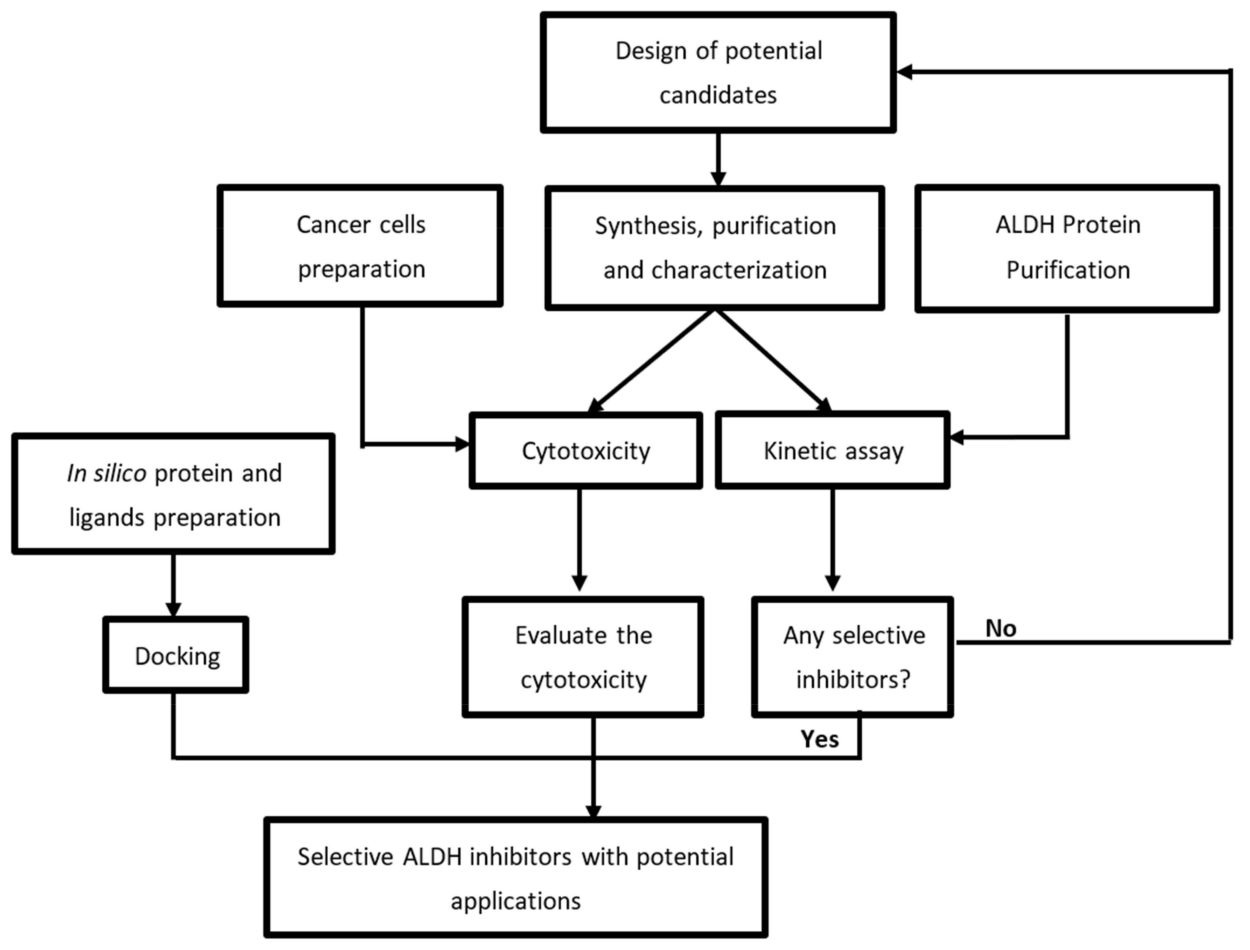
Flow chart describing the discovery of ALDH1A3 selective inhibitors.

Hence, the design of this library was based on the physiological substrate resemblance, aiming to achieve a high affinity for the substrate pocket but with an inhibitory effect. Scheme 1 shows a flow chart that describes our process of discovering a selective inhibitor for the ALDH1A3 isoform.

## 2. Results and Discussion

### 2.1. Chemistry

**Scheme 2:**
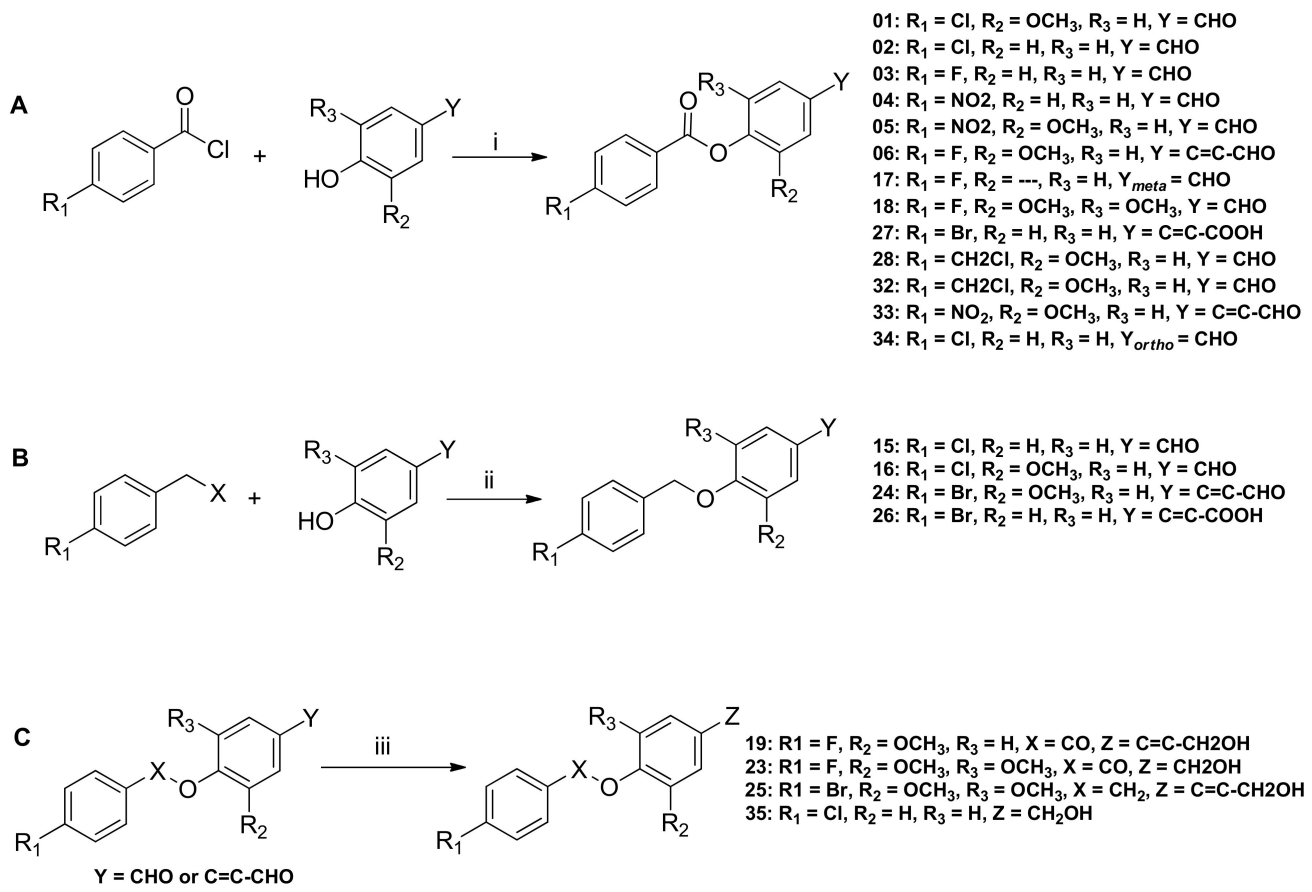
Synthesis of the target ABMM compounds: (**A**) i: DMF, trimethylamine, r.t, overnight; (**B**) ii: DMF, K_2_CO_3_, 80 °C, overnight; (**C**) iii: NaBH_4_, methanol, r.t, 2 h.

Several derivatives containing the phenylbenzoate and (benzyloxy)benzene scaffolds were synthesized by esterification (Scheme 2A) and O-alkylation (Scheme 2B) reactions, respectively. Compounds containing an aldehyde functionality were reduced to alcohols using sodium borohydride (Scheme 2C). The final compounds were either alcohols, aldehydes or carboxylic acids (Y group in Scheme 1) to enable their exploration as substrates with the purpose of inhibiting their catalytic functions, either reversibly or irreversibly. These compounds have the Y group connected to an aromatic ring either directly or through an olefinic bond in order to partially mimic the double-bond conjugation of retinoids. In addition, these compounds have two aromatic rings connected together by either an ester or a methyloxy group.

### 2.2. Inhibition Screening

Nine compounds were tested at 10 µM for their inhibitory effect against ALDH1A1, ALDH1A3 and ALDH3A1, as shown in Table 1. These compounds were selected to obtain preliminary data according to their structural diversity. The ALDH activity was determined by an enzymatic assay in which the substrate is oxidized in the presence of the cofactor NAD(P)^+^, generating NAD(P)H, which is measured fluorimetrically with an excitation at 340 nm and emission at460 nm. The measured fluorescence increase per time unit is directly proportional to the ALDH activity rate. Hexanal or 4-NBA was used as the substrate due to their high catalytic efficiency [30]. The inhibition screening was preferably performed at a saturating substrate concentration except for ALDH1A3, where compounds were also tested at a substrate concentration near the *K_m_* value in order to obtain a wider preliminary inhibitory activity.

As shown in Table 1, none of the ABMM compounds showed significant inhibition of ALDH1A1 activity (lower than 30% of the remaining activity at 10 µM), with the lowest values 48% and 42% being obtained for compounds ABMM-15 and ABMM-16, respectively. However, these two compounds showed promising inhibitory potency toward ALDH1A3, in which the remaining activity was 0.14% for ABMM-15 and 4.27% for ABMM-16. ABMM-18 also resulted in a low remaining activity (16%) against ALDH1A3 at a *K_m_* concentration, followed by ABMM-1 with a remaining activity of 21%.

Since ABMM-15 and ABMM-16 were the most promising compounds regarding an ALDH1A3 selective inhibition, a time-dependent inhibition assay was performed in order to study the possibility of a slow binding. Hence, each compound was pre-incubated for 20 min as opposed to the usual 5 min before initiating the reaction. No significant differences were observed, as shown in Table 2.

Following these findings, we decided to explore the structure–activity relationships (SAR) for the ALDH1A3 selectivity by ABMMs. Both compounds ABMM-15 and ABMM-16 have a methyloxy (-CH_2_O-) group as a linker between the two benzene rings, while the linker in all other tested compounds is an ester group. This structural difference seemed to contribute to a remarkable difference in the inhibitory activity. For example, ABMM-2 and ABMM-1 share a structural similarity with ABMM-15 and ABMM-16, respectively, but have an ester linker instead of a methyloxy, which provided a much higher remaining activity (94% of ABMM-2 vs. 0.14% of ABMM-15, and 21% of ABMM-1 vs. 4.27% of ABMM-16). This finding highlights the importance of the methyloxy linkers in the inhibitory binding with the ALDH1A3 active site. In addition, the aromatic substitution seemed to have an effect on the inhibitory activity. For example, ABMM-16 is structurally different from ABMM-15 by having a methoxy group at a position meta to the aldehyde, which provided a higher remaining activity (0.14% vs. 4.27%). However, ABMM-1 has an extra methoxy group when compared to ABMM-2, yet it provided a lower remaining activity (21% vs. 94%). Unexpectedly, ABMM-18 has two methoxy groups at both positions meta to the aldehyde group, but it resulted in 16% of the remaining activity, which is lower than both ABMM-1 and ABMM-2.

An esterase activity has been described for the ALDH1A and ALDH3 subfamilies to a certain extent, in addition to the dehydrogenase activity. As some of the compounds have an ester linker and show no inhibitory activity on ALDH1A3, they could act as substrates, either for the aldehyde or for the ester group [36,37,38].

None of the compounds seemed to cause a significant inhibition of ALDH3A1 activity and thus they were not considered for further characterization. Moreover, some compounds, such as ABMM-2 and ABMM-15, showed a remaining activity higher than 100% for ALDH3A1, suggesting a potential substrate activity. In fact, the structures appear to be in a good agreement with the preferred type of aldehyde substrate for the ALDH3A1 pocket, as they are small aromatic aldehydes.

### 2.3. Determination of Half-Maximal Inhibitory Concentrations (IC_50_)

**Figure 2:**
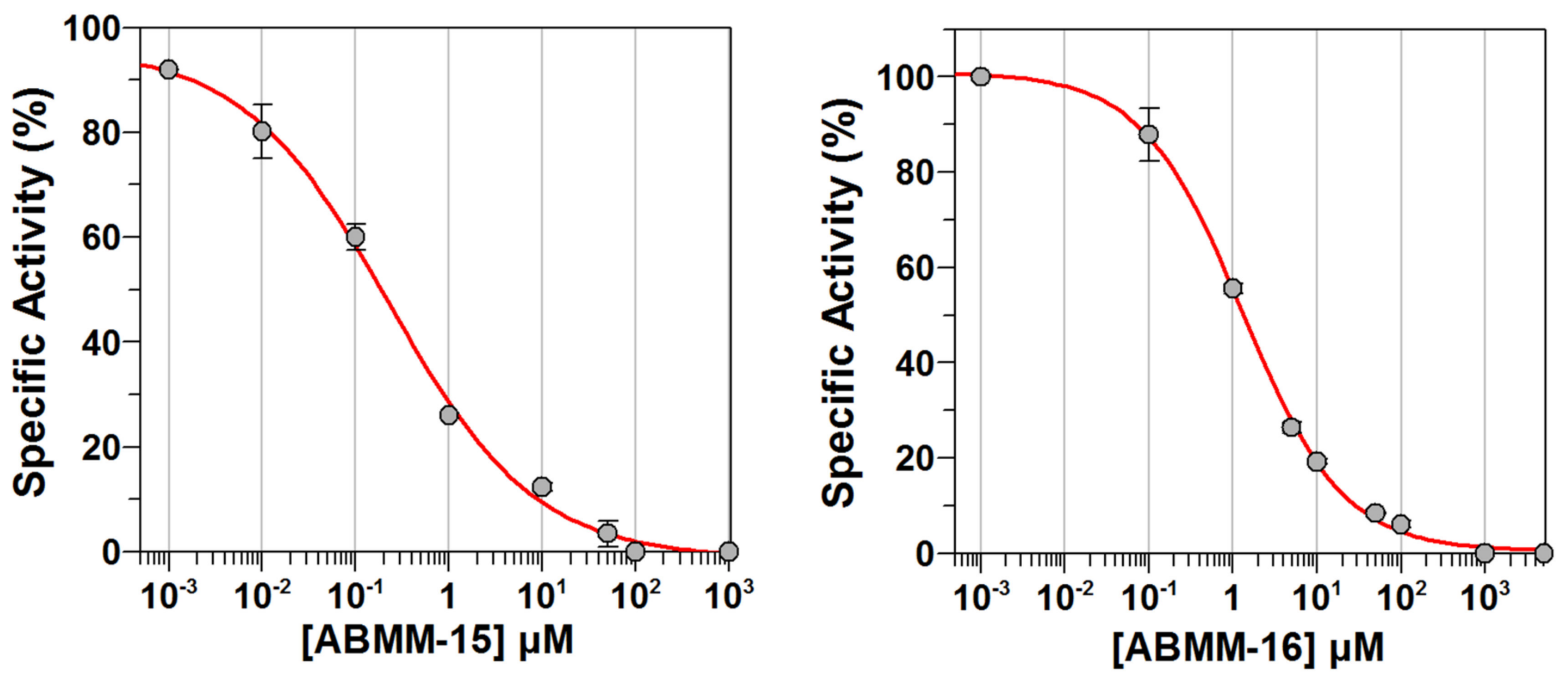
Representative sigmoidal curves to calculate the IC_50_ values of ABMM-15 (**left**) and ABMM-16 (**right**) against ALDH1A3 at a saturating concentration of hexanal (250 µM). The percentage of specific activity is plotted against the logarithm of inhibitor concentration. Experimental values are shown as the mean ± SE of duplicate experiments.

The IC_50_ values were calculated for compounds ABMM-15 and ABMM-16 as the best fits for ALDH1A3, with the results shown in Figure 2. Experiments for both compounds were performed at a saturating concentration of hexanal and resulted in IC_50_ values of 0.23 ± 0.05 µM and 1.29 ± 0.10 µM for ABMM-15 and ABMM-16, respectively.

### 2.4. Cytotoxicity Experiments

NSCLC cell lines, including A549 and H1299, were chosen because of their well-known expression level of ALDH isoforms, where it has been proven that A549 is highly expressing ALDH1A1 and ALDH3A1, but with a lower level of ALDH1A3, while H1299 is deficient in these isoforms and other family members [39,40]. In addition, as these cancer cell lines are commonly used as in vitro model for exploring new therapeutics [40], the synthesized compounds were investigated on these cell-lines to determine their effect on cell survival using the MTT assay after 24 and 96 h of exposure. The results are shown in Table 3 for 96 h of exposure. The highest concentration used for the compounds was 60 µM due to solubility issues in dimethylsulfoxide (DMSO), so that the final concentration of DMSO did not exceed the acceptable non-toxic concentration of 0.2%.

The ALDH3A1-expressing A549 cells exposed to ABMM-15 and ABMM-16 did not seem to be affected by exposure to these ALDH-affinic compounds. A possible explanation might be that these compounds are detoxified via conversion to their corresponding carboxylic acids.

**Figure 3:**
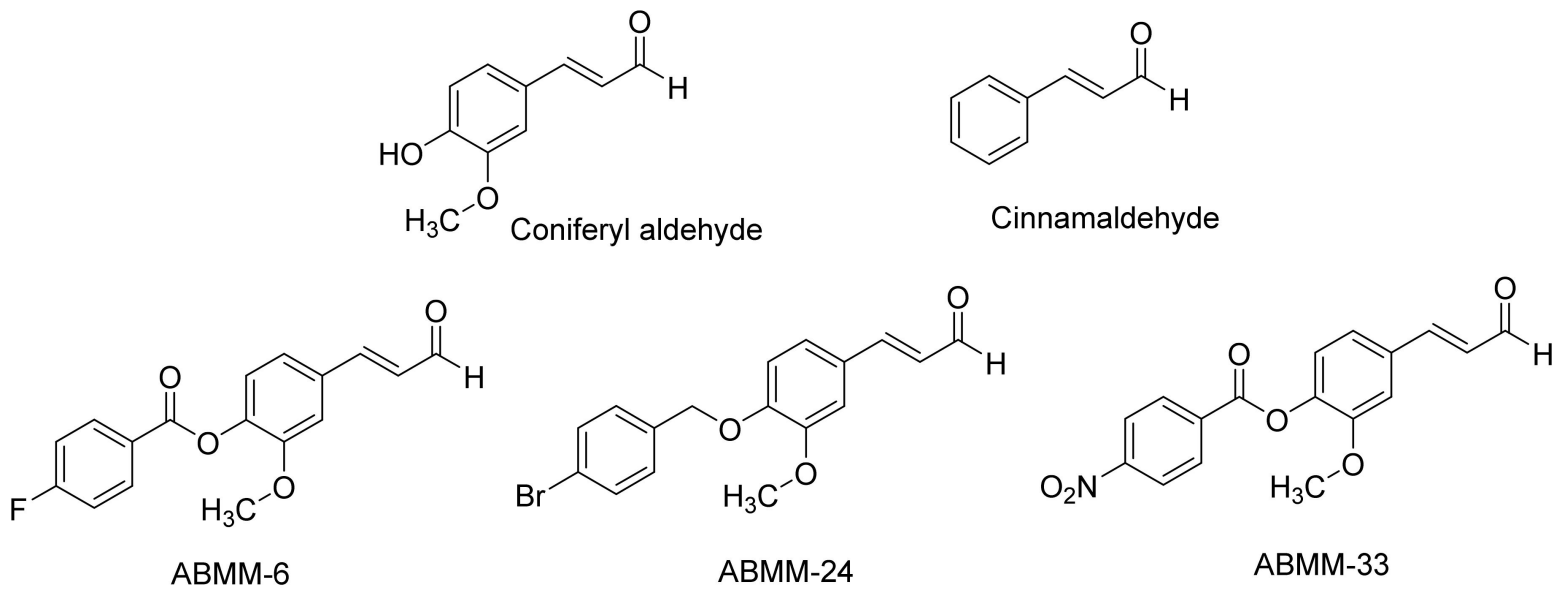
Chemical structures of selected compounds.

In contrast to their effect on A549 cells, some other compounds showed a considerable toxicity on H1299 cells, including ABMM-6, ABMM-24, ABMM-28, ABMM-32 and ABMM-33. This may be related to the fact that A549 cells express high levels of ALDHs, which may be involved in the compound detoxification. Compounds ABMM-6, ABMM-24 and ABMM-33 are derivatives of coniferyl aldehyde, which contains a cinnamaldehyde nucleus (Figure 3). Cinnamaldehyde has been found to be cytotoxic on several cancer cell lines, such as leukemia K562 [41] and breast cancer MCF-7 cells [42]. Furthermore, cinnamaldehyde has been found to be a promising adjuvant candidate in combination with 5-fluorouracil and oxaliplatin against colorectal carcinoma [43], and to enhance the apoptosis induced by hyperthermia against the A549 cells [44] and by doxorubicin on glioblastoma cells (U87MG) [45]. This cytotoxicity of cinnamaldehyde may be due to the presence of an electrophilic Michael acceptor (α,β-unsaturated aldehyde) pharmacophore, which has been anticipated to provide this scaffold with a potential anticancer activity [46]. However, coniferyl aldehyde has not yet been reported to be cytotoxic and has been found non-toxic on MCF-7 and NCI-H187 cell lines [47]. In this study, the synthesized coniferyl aldehyde derivatives have an extended benzene ring linked to a hydroxyl group by either an ester group (as in ABMM-6) or a methyloxy group (as in ABMM-24), with no significant difference in their cytotoxicity according to their IC_50_ values of 14.0 and 13.7 µM, respectively. This elongation by an extra benzene ring seemed to play an important role in the cytotoxicity of the compounds. In addition, all these molecules share the aldehyde functionality, which also seemed to participate in the cytotoxicity. For example, when the aldehyde group in ABMM-24 was replaced by an alcohol (e.g., ABMM-25) or by a carboxylic acid (e.g., ABMM-26), the cytotoxicity was significantly reduced.

Compounds ABMM-28 and ABMM-32 have similar chemical structures to ABMM-1 and ABMM-2, respectively, but with a *p*-chloromethyl group instead of chlorine. Since both ABMM-1 and ABMM-2 showed no significant toxicity on H1299 cells, the presence of chloromethyl in both ABMM-28 and ABMM-32 seemed to be important for the compound cytotoxicity, possibly acting as an alkylating moiety. Interestingly, compounds ABMM-15 and ABMM-16 showed no significant toxicity on either cell lines. Therefore, compounds ABMM-15 and ABMM-16 are worth investigating in combination therapies with anticancer drugs, such as doxorubicin and paclitaxel, where the inhibition of ALDH has been found to reduce chemoresistance in breast cancer cells [48].

Compound ABMM-23, which is an alcohol version of compound ABMM-18, is the only compound that showed a higher toxicity on A549 cells (IC_50_ = 51.0 µM) than on H1299 cells (IC_50_ > 60 µM), when compared with compound ABMM-18, which showed no toxicity on either cell line at the maximum concentration of 60 µM. This can be explained by the fact that ABMM-23 did not act as a substrate for ALDHs, while ABMM-18 might have had a mixed activity of both substrate and inhibitor.

### 2.5. In Silico Study

**Figure 4:**
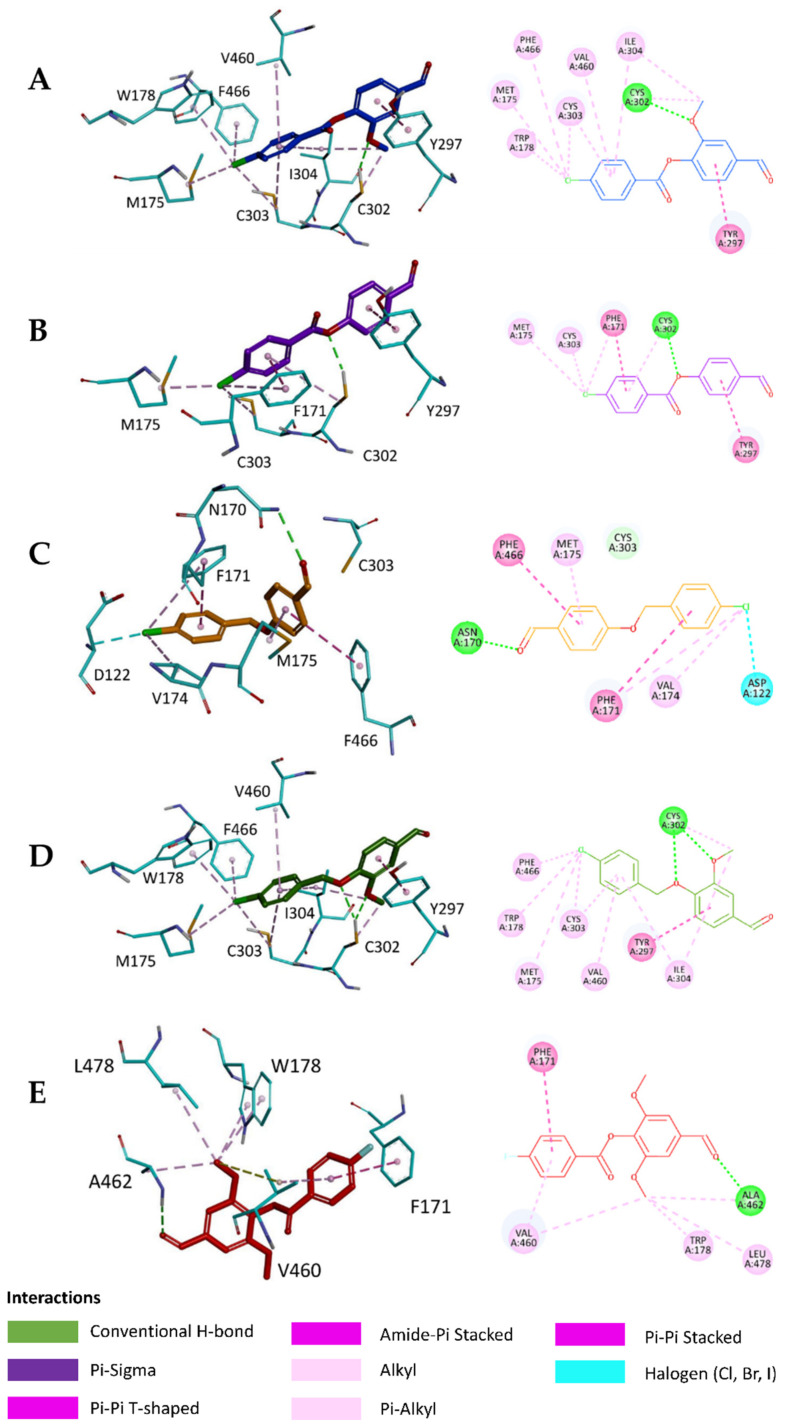
Molecular docking of: (**A**) ABMM-1 (blue), (**B**) ABMM-2 (purple), (**C**) ABMM-15 (orange), (**D**) ABMM-16 (green) and (**E**) ABMM-18 (red) into the ALDH1A1 binding site (cyan-colored residues).

**Figure 5:**
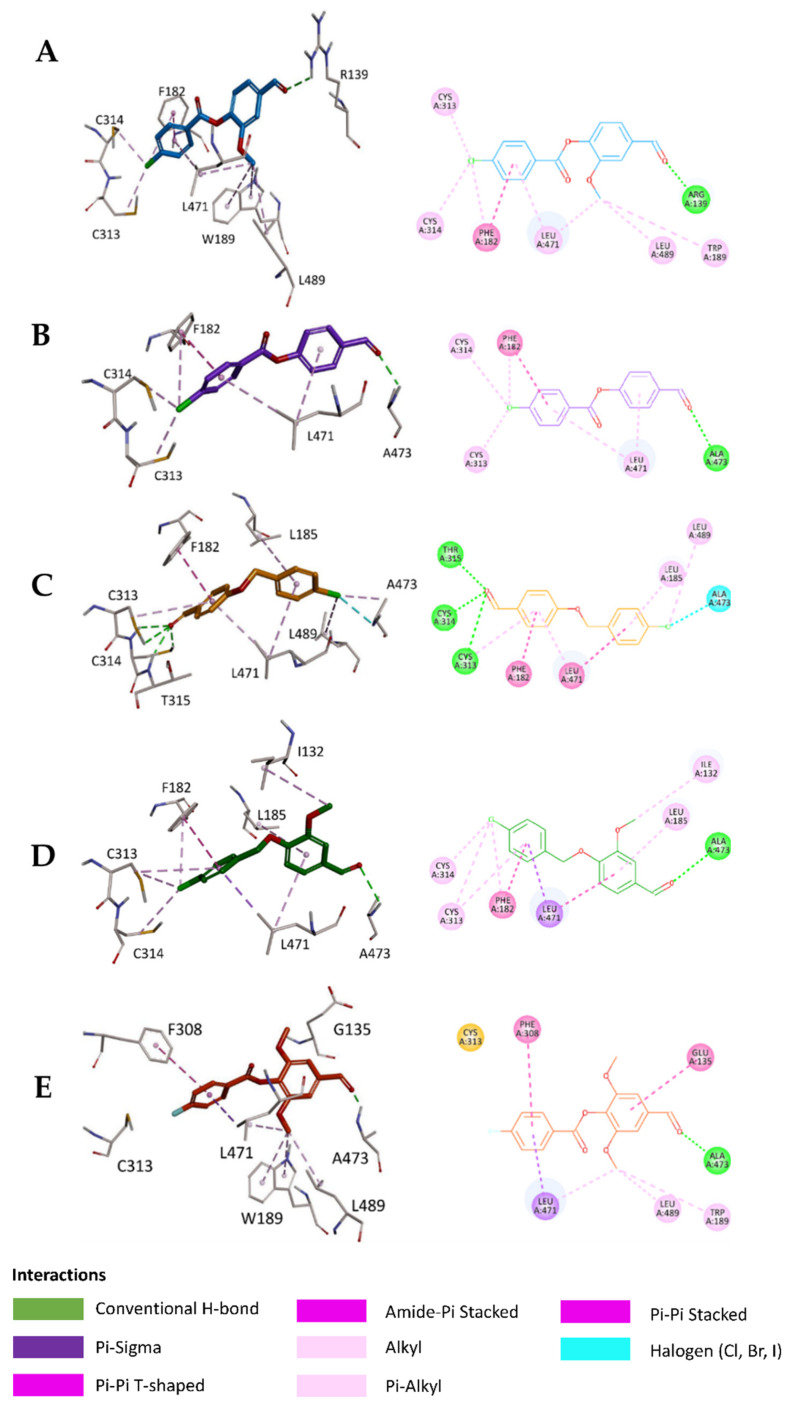
Molecular docking of: (**A**) ABMM-1 (cyan), (**B**) ABMM-2 (purple), (**C**) ABMM-15 (orange), (**D**) ABMM-16 (green) and (**E**) ABMM-18 (red) into the ALDH1A3 binding site (gray-colored residues).

**Figure 6:**
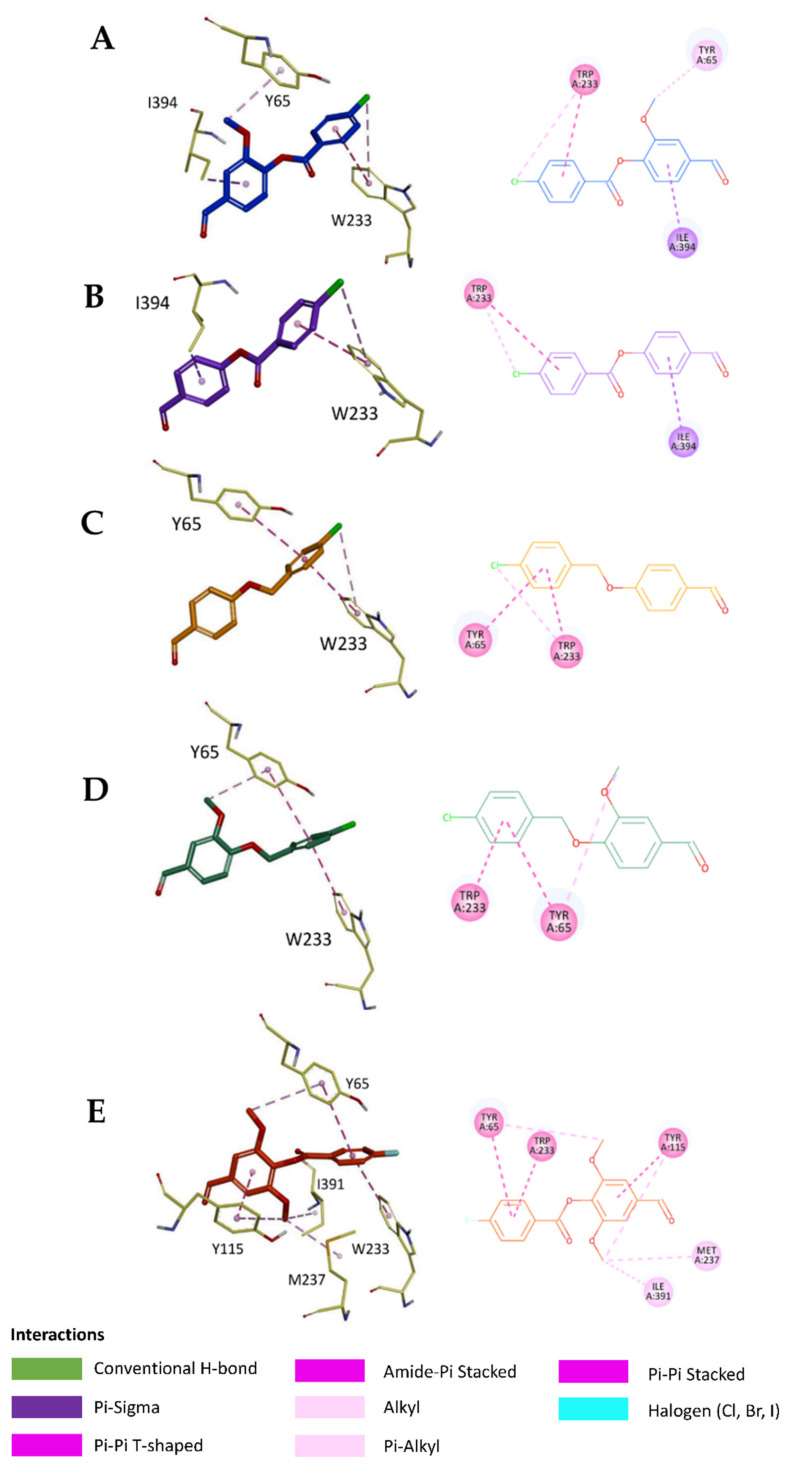
Molecular docking of: (**A**) ABMM-1 (blue), (**B**) ABMM-2 (purple), (**C**) ABMM-15 (orange), (**D**) ABMM-16 (green) and (**E**) ABMM-18 (red) into the ALDH3A1 binding site (yellow-colored residues).

Docking studies were performed on some selected compounds of the series, employing Glide program (Maestro, Schrodinger) with a Standard Precision (PS) scoring function. The structures of three proteins (ALDH1A1, ALDH1A3 and ALDH3A1) were downloaded from the protein data (see the materials and methods for details). These docking studies were originally planned to better understand the potential binding of ABMM-15 and ABMM-16 to the ALDH1A3 binding site, in comparison to their binding to ALDH1A1 and ALDH3A1 binding sites. To compare the results with other ABMM compounds, three more compounds (ABMM-1, ABMM-2 and ABMM-18), which showed reasonable biological activities, were also selected for the docking studies to the three ALDH isoforms. The results are shown below in Figure 4, Figure 5 and Figure 6.

**Figure 7:**
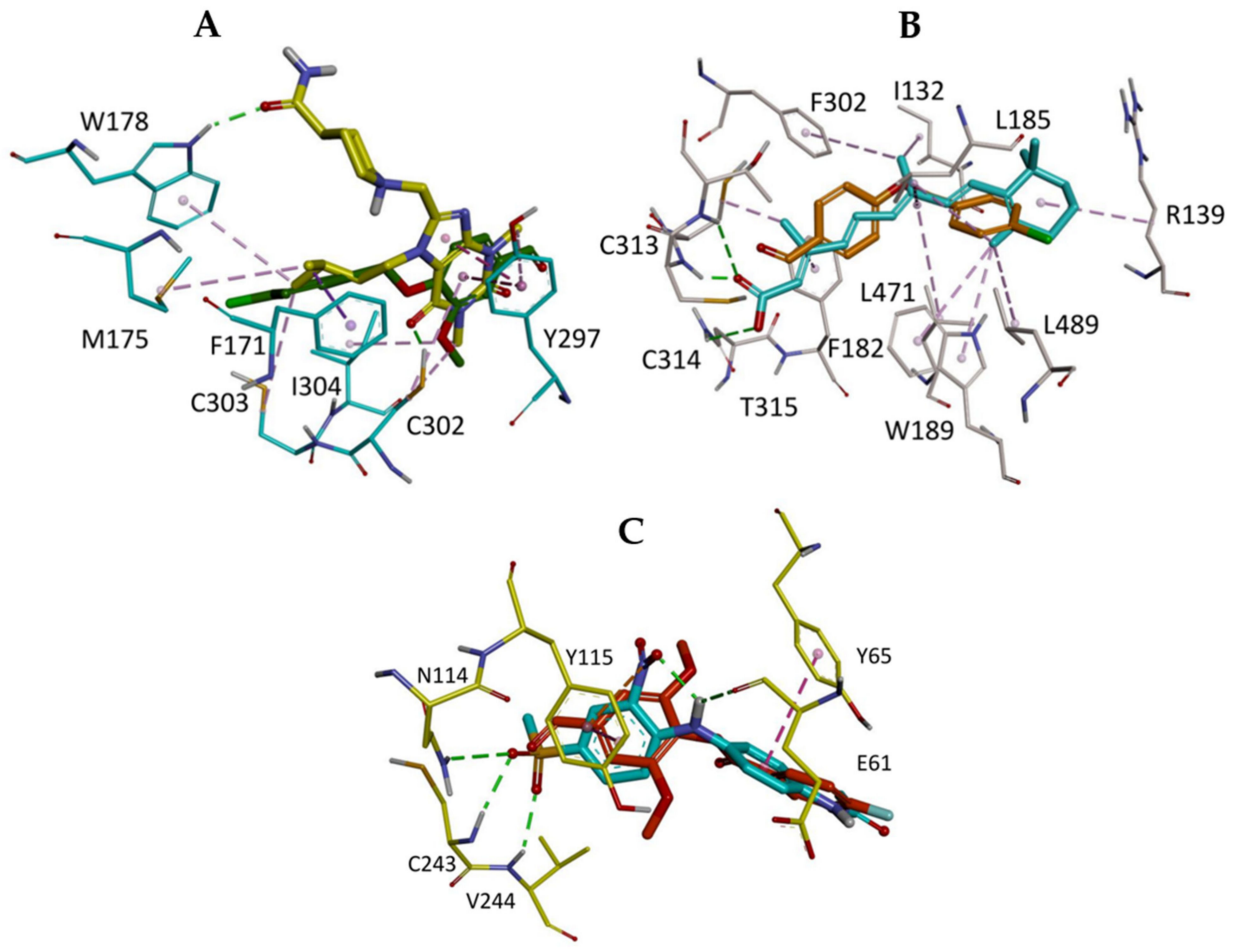
Superimposition of (**A**) co-crystallized inhibitor (green) of ALDH1A1 (cyan residues) and ABMM-16 (yellow), (**B**) substrate (RA, green) of ALDH1A3 (gray residues) and ABMM-15 (orange) and (**C**) co-crystallized inhibitor (cyan) of ALDH3A1 (yellow residues) and ABMM-18 (Red). Only the interactions of co-crystallized inhibitors/substrate are shown for clarity.

As depicted in Figure 4, both ABMM-1 and ABMM-2 participated in H-bonding interactions with catalytic C302 of the ALDH1A1 binding site; however, the placement of the *p*-chlorophenyl ring seemed to be marginally different in both cases. The *p*-chlorophenyl ring of ABMM-1 was involved in multiple hydrophobic contacts with M175, W178, C303, V460 and F466, whereas the *p*-chlorophenyl of ABMM-2 established hydrophobic contacts with M175, F171, C302 and C303 within the ALDH1A1 binding site. A greater no. of hydrophobic contacts of ABMM-1 correspond to the docking score of −7.709 kcal/mol (Table 4). The interactions of ABMM-2 with C302, C303 and F171 are predicted to be crucial, which may contribute to an increase in binding affinity towards the ALDH1A1 improved [S]et saturation of ABMM-2 over ABMM-1. The central benzaldehyde core of both ABMM-1 and ABMM-2 formed face-to-face pi–pi stacking with Y297. On comparison of ABMM-1 and ABMM-2 with the dihydropurine-based co-crystallized inhibitor of ALDH1A1, the co-crystallized inhibitor also depicted van der Waals interactions with F171, Y297, C302 and C303 (Figure 7A). The central benzaldehyde core of ABMM-15 oriented oppositely compared to ABMM-1 and ABMM-2 within the ALDH1A1 binding site and did not show any H-bond contacts. The hydrophobic interactions with F171, V174, M175 and F466 were constituted by ABMM-15, followed by H-bond contact with N170 and halogen bond formation with D122. The presence of these two additional interactions (H-bond and halogen) signifies the improved activity of ABMM-15 over ABMM-1 and ABMM-2. The best compound of the series (ABMM-16) was found to be in complete agreement with the highest docking score of −8.157 kcal/mol. The *p*-chlorophenyl moiety of ABMM-16 established hydrophobic contacts with M175, W178, C303, V460 and F466, while its benzaldehyde core was involved in face-to-face pi–pi stacking with Y297. Apart from hydrophobic contacts, the methoxy group and methoxy linker of ABMM-2 formed a couple of H-bonds with C302, which may contribute to the enrichment in the binding affinity of ABMM-16 over other compounds. The presence of the methyloxy linker in place of the ester linker supported a favorable binding disposition. ABMM-18 formed a relatively lesser no. of H-bond and hydrophobic interactions within the ALDH1A1 binding pocket, which corresponds to its poor binding affinity and docking score (−6.193 kcal/mol).

Figure 5 shows the docking poses of the ABMM compounds within the ALDH1A3 binding site. The p-halogen rings of ABMM-1, ABMM-2, ABMM-16 and ABMM-18 were accommodated into the hydrophobic cavity, whereas their benzaldehyde cores were placed at the entrance of the ALDH1A3 binding site. The *p*-chlorophenyl ring of ABMM-1 constituted hydrophobic contacts with F182, C313, C314 and L471, while the methoxy group of its benzaldehyde core facilitated hydrophobic contacts with W189 and L189. Furthermore, the aldehyde group of ABMM-1 formed a H-bond with R139. A similar H-bond and hydrophobic interaction pattern was observed for ABMM-2, ABMM-16 and ABMM-18; however, ABMM-18 demonstrated a lesser no. of H-bond and hydrophobic contacts. In particular, the presence of methoxy linker on the second-best compound of the series, ABMM-16, favored the binding to ALDH1A3, establishing hydrophobic contacts with L185 and L489. The best compound of the series, ABMM-15, was oriented oppositely relative to other compounds. The aldehyde of ABMM-15 constituted H-bond contacts with the C302, C303 and T315. These H-bond interactions are believed to crucially contribute to the binding affinity with the target, as the co-crystallized substrate of ALDH1A3 (RA) also displayed a similar interaction with C314 and T315 (Figure 7B). The central benzaldehyde core was involved in pi–pi interactions with F182 and L471, whereas the *p*-chlorophenyl ring of ABMM-1 implicated halogen bond interaction with A473 and hydrophobic contacts with L185 and L489. Despite the docking score of ABMM-16 (−8.306 kcal/mol) being relatively higher than ABMM-1 (−7.804 kcal/mol/), the presence of crucial H-bond contacts between ABMM-1 and ALDH1A3 hydrophobic cavity is envisioned to contribute to the improved binding affinity towards ALDH1A3.

As shown in Figure 6, compounds ABMM-1, ABMM-2, ABMM-15, ABMM-16 and ABMM-18 presented similar binding modes within the ALDH3A1 binding site. The p-halogen ring of these compounds established at least one pi–pi stacking interaction with W233. All these compounds displayed a poor interaction profile within the ALDH3A1 binding site compared to ALDH1A1 and ALDH1A3, which further resemble the docking scores and weak binding affinities. Only ABMM-18 was able to demonstrate multiple van der Waals contacts (Y65, Y115, W233, M237 and I391), which was also partly shown by the nitrophenylamine-based co-crystallized inhibitor of ALDH3A1 (Y65, Y115) (Figure 7C), but it still lacked ALDH3A1 inhibition.

As shown in Figure 7, the three most active compounds (ABMM-16 for ALDH1A1, ABMM-15 for ALDH1A3 and ABMM-18 for ALDH3A1) were compared with the crystallographic pose of the bound inhibitor/substrate from their respective proteins. Figure 7A depicts the crystallographic binding mode of the dihydropurine-based inhibitor, which formed crucial face-to-face pi–pi stacking with Y297. This interaction is identically formed by the benzaldehyde core of ABMM-16. One of the oxo from the dihydropurine-based co-crystallized inhibitor of ALDH1A1 established an important H-bond with C302, which is also shown by the methoxy-group of the ABMM-16. Hydrophobic interactions with M175, W178, C303 and I304 were shared by both the co-crystallized inhibitor and ABMM-16. As shown in Figure 7B, the co-crystallized substrate of ALDH1A3, RA and ABMM-15 were superimposed. RA constituted H-bond interactions with C314 and T315 in the ALDH1A3 binding site, similar to ABMM-15 (Figure 5C). The hydrophobic moiety of RA formed van der Waals contacts with L185, L489 and L471, which were also constituted by ADMM-15 within the ALDH1A3 binding site. Figure 7C displays the binding orientation of nitrophenylamine-based co-crystallized inhibitor, superimposed with ABMM-18 within the ALDH3A1 binding site. The co-crystallized inhibitor demonstrated important H-bond contacts with V114, C243 and V244 that are completely missing in ABMM-18, probably satisfying the poor inhibition profile of ABMM-18 against ALDH3A1. Two pi–pi interactions were found to be shared by both the co-crystallized inhibitor and ABMM-18, which may not be sufficient for ABMM-18 to effectively contribute to the increase in binding affinity; thus, ABMM-18 requires further structure-based hit optimization.

## 3. Materials and Methods

All materials and reagents were used as received with no further purification. 4-hydroxybenzaldehyde, syringaldehyde, coniferyl aldehyde, 4-fluorobenzoyl chloride, 4-chlorobenzoyl chloride, 4-NBA, hexanal, HEPES, EDTA, DTT, MgCl_2_, Tris-HCl, MTT and 4-nitorobenzoyl chloride were purchased from (Sigma, Dorset, UK); NAD^+^, NADH, NADP^+^ and NADPH were purchased from (Apollo Scientific, Bredbury, UK); 3-hydroxybenzaldehyde, 2-hydroxybenzaldehyde, Vanillin, and 4-(chloromethyl)benzoyl chloride were purchased from (Acros Organics, Belgium, USA); A549 and H1299 human NSCLC cell lines were purchased from ATCC with LOT number 70018877 and 70008730, respectively; PBS, RPMI-1640 medium, fetal bovine serum (FBS) and L-glutamine were purchased from (Euroclone, Pero, Italy); DMSO was purchased from TEDIA (USA). Chemical reactions were monitored by analytical thin layer chromatography using Merck 9385 silica gel 60 F254 aluminum-backed plates through visualizing the spotted plates under ultraviolet (UV) at 254 and 366 nm. Intermediates and final products were purified by column chromatography using silica gel (pore size 60 Å, 40–63 µm particle size). Proton and carbon NMR were analyzed for all intermediates and final products on Bruker AMX400 (400 MHz and 300 MHz) nuclear magnetic resonance spectrometer. Chemical shifts were reported in parts per million (δ, ppm) downfield from internal TMS. Coupling constants (J) were expressed in Hertz (Hz). High-resolution mass spectra were obtained by the Engineering and Physical Sciences Research Council (EPSRC) mass spectrometry service, Swansea, UK. The spectra for the products can be found in the Supplementary Materials. Chemical structures were drawn using ChemDraw (version 18.0.0231). Melting points were measured with a Gallenkamp melting point apparatus. Dose–response curves were generated using the GraphPad Prism 7 Software; optical densities were then measured on a SynergyHTX^®^ spectrophotometer; IC_50_ values were calculated by non-linear fitting of the experimental data to a sigmoidal plot using GraFit 5.0 (Erithacus software); enzymatic activity was measured using a Cary Eclipse Varian fluorimeter.

### 3.1. Chemistry

#### 3.1.1. General Procedure for the Synthesis of Compounds ABMM-1–6, 17, 18, 27, 28, 32–34

To a solution of the phenolic starting material (1.0 eq.) in 10 mL DMF, trimethylamine (2.0 eq.) was added, and the solution was stirred at room temperature for 30 min. To the stirred solution, acid chloride (1.0 eq.) was added slowly dropwise or by portions, and the resulting solution was stirred at room temperature for one hour. After the reaction completion, 20 mL saturated aqueous solution of sodium carbonate was added portionwise. The resulting precipitate was filtered, dried and purified by either recrystallization or column chromatography, affording the desired product.

#### 3.1.2. General Procedure for the Synthesis of Compounds ABMM-15, 16, 24, 26

To a solution of the phenolic starting material (1.0 eq.) and potassium carbonate (1.5 eq.) in 10 mL DMF, benzyl halide (1.0 eq.) was added, and the resulting solution was stirred overnight at 70 °C. After the reaction was completed, volume of DMF was reduced by evaporation under vacuum. To the resulting solution, 20 mL water was added and the resulting precipitation was filtered, dried and recrystallized from ethanol, affording the desired product.

#### 3.1.3. General Procedure for the Synthesis of Compounds ABMM-19, 23, 25, 35

The starting aldehyde (1.0 eq.) was dissolved in 10 mL absolute ethanol and stirred for 10 min in ice bath. NaBH4 (3.0 eq.) was then added slowly over a period of 10 min. The reaction was followed by TLC and when completed, 10 mL of distilled water was added and the mixture was stirred at 0 °C for 10 min. The product was then extracted by chloroform (3 × 20 mL). The collected organic phase was dried over magnesium sulfate and filtered. The solvent was evaporated under vacuum, affording the desired product.

##### 4-Formyl-2-methoxyphenyl 4-Chlorobenzoate (ABMM-1)

Off-white solid; 92% yield; mp 98–100 °C. ^1^H NMR (400 MHz, CDCl_3_) δ 9.92 (s, 1H, CHO), 8.08 (d, *J* = 8.8 Hz, 2H, ArH), 7.50–7.47 (m, 1H, ArH), 7.48–7.46 (m, 1H, ArH), 7.44 (d, *J* = 8.8 Hz, 2H, ArH), 7.28 (d, *J* = 7.9 Hz, 1H), 3.82 (s, 3H, CH_3_). ^13^C NMR (101 MHz, CDCl_3_) δ 191.05, 163.34, 152.13, 145.00, 140.46, 135.40, 131.78, 129.05, 127.30, 124.79, 123.51, 110.92, 56.16. HRMS (ESI) calculated for C_15_H_11_ClO_4_ [M + H]^+^ 291.03459, found 291.04347.

##### 4-Formylphenyl 4-Chlorobenzoate (ABMM-2)

White solid; 91% yield; mp 111–113 °C. ^1^H NMR (400 MHz, CDCl_3_) δ 9.96 (s, 1H, CHO), 8.07 (d, *J* = 8.8 Hz, 2H, ArH), 7.91 (d, *J* = 8.7 Hz, 2H, ArH), 7.44 (d, *J* = 8.8 Hz, 2H, ArH), 7.34 (d, *J* = 8.7 Hz, 2H, ArH). ^13^C NMR (101 MHz, CDCl_3_) δ 190.89, 163.69, 155.45, 140.68, 134.22, 131.65, 131.32, 129.15, 127.37, 122.48. HRMS (ESI) calculated for C_14_H_9_ClO_3_ [M + H]^+^ 261.02402, found 261.03153.

##### 4-Formylphenyl 4-Fluorobenzoate (ABMM-3)

White solid; 53% yield; mp 75–77 °C. ^1^H NMR (400 MHz, CDCl_3_) δ 10.03 (s, 1H, CHO), 8.23 (dd, *J* = 8.9, 5.4 Hz, 2H, ArH), 7.98 (d, *J* = 8.6 Hz, 2H, ArH), 7.41 (d, *J* = 8.6 Hz, 2H, ArH), 7.21 (dd, *J* = 15.8, 8.9 Hz, 2H, ArH). ^13^C NMR (101 MHz, CDCl_3_) δ 191.06, 169.05 (d, *J* = 246.9 Hz), 165.28, 155.67, 134.31, 133.10 (d, *J* = 9.6 Hz), 131.45, 125.31 (d, *J* = 3.2 Hz), 122.64, 116.26, (d, *J* = 22.1 Hz).

##### 4-Formylphenyl 4-Nitrobenzoate (ABMM-4)

White solid; 85% yield; mp 197–200 °C. ^1^H NMR (400 MHz, CDCl_3_) δ 9.98 (s, 1H, CHO), 8.32 (s, 4H, ArH), 7.94 (d, *J* = 8.6 Hz, 2H, ArH), 7.37 (d, *J* = 8.6 Hz, 2H, ArH). ^13^C NMR (101 MHz, CDCl_3_) δ 190.77, 162.71, 155.04, 151.15, 134.52, 134.30, 131.44, 131.41, 123.88, 122.33. HRMS (ESI) calculated for C_14_H_9_NO_5_ [M + H]^+^ 286.06372, found 286.07122.

##### 4-Formyl-2-methoxyphenyl 4-Nitrobenzoate (ABMM-5)

White solid; 88% yield; mp 174–176 °C. ^1^H NMR (400 MHz, CDCl_3_) δ 9.93 (s, 1H, CHO), 8.34–8.29 (m, 4H, ArH), 7.51–7.47 (m, 2H, ArH), 7.31 (d, *J* = 7.8 Hz, 1H), 3.84 (s, 3H, CH_3_). ^13^C NMR (101 MHz, CDCl_3_) δ 190.94, 162.33, 151.92, 151.06, 144.56, 135.68, 134.25, 131.54, 124.75, 123.79, 123.34, 111.01, 56.19.

##### (*E*)-2-Methoxy-4-(3-oxoprop-1-en-1-yl)phenyl 4-Fluorobenzoate (ABMM-6)

White solid; 82% yield; mp 118–120 °C. ^1^H NMR (400 MHz, CDCl_3_) δ 9.65 (d, *J* = 7.6 Hz, 1H, CHO), 8.16 (dd, *J* = 9.0, 5.4 Hz, 2H, ArH), 7.41 (d, *J* = 15.9 Hz, 1H, CH), 7.18–7.09 (m, 5H, ArH), 6.63 (dd, *J* = 15.9, 7.6 Hz, 1H, CH), 3.80 (s, 3H, CH_3_). ^13^C NMR (101 MHz, CDCl_3_) δ 193.45, 166.28 (d, *J* = 255.4 Hz), 163.46, 151.86, 151.81, 142.35, 133.09, 133.07, 132.98, 128.83, 125.25 (d, *J* = 3.0 Hz), 122.78 (d, *J* = 171.1 Hz), 115.86 (d, *J* = 22.1 Hz), 111.49, 56.03. HRMS (ESI) calculated for C_17_H_13_FO_4_ [M + H]^+^ 301.07979, found 301.08612.

##### 4-((4-Chlorobenzyl)oxy)benzaldehyde (ABMM-15)

White solid; 88% yield; mp 69–71 °C. ^1^H NMR (400 MHz, CDCl_3_) δ 9.82 (s, 1H, CHO), 7.77 (d, *J* = 8.9 Hz, 2H, ArH), 7.35–7.25 (m, 4H, ArH), 6.99 (d, *J* = 8.7 Hz, 2H, ArH), 5.05 (s, 2H, CH_2_). ^13^C NMR (101 MHz, CDCl_3_) δ 190.75, 163.44, 134.46, 134.22, 132.03, 130.32, 128.95, 128.81, 115.13, 69.48. HRMS (ESI) calculated for C_14_H_11_ClO_2_ [M + H]^+^ 247.04476, found 246.05249.

##### 4-((4-Chlorobenzyl)oxy)-3-methoxybenzaldehyde (ABMM-16)

White solid; 86% yield; mp 77–80 °C. ^1^H NMR (400 MHz, CDCl_3_) δ 9.78 (s, 1H, CHO), 7.37 (d, *J* = 1.8 Hz, 1H, ArH), 7.33 (dd, *J* = 8.2, 1.8 Hz, 1H, ArH), 7.31–7.27 (m, 4H, ArH), 6.89 (d, *J* = 8.2 Hz, 1H, ArH), 5.13 (s, 2H, CH_2_), 3.88 (s, 3H, CH_3_). ^13^C NMR (101 MHz, CDCl_3_) δ 190.88, 157.44, 134.67, 133.22, 130.20, 128.96, 128.60, 126.50, 112.43, 109.46, 70.14, 56.08. HRMS (ESI) calculated for C_15_H_13_ClO_3_ [M + H]^+^ 277.05532, found 277.06171.

##### 3-Formylphenyl 4-Fluorobenzoate (ABMM-17)

White solid; 85% yield; mp 58–60 °C. ^1^H NMR (400 MHz, CDCl_3_) δ 9.97 (s, 1H, CHO), 8.16 (dd, *J* = 9.0, 5.4 Hz, 2H, ArH), 7.74 (ddd, *J* = 7.6, 2.4, 1.3 Hz, 1H, ArH), 7.71–7.64 (m, 1H, ArH), 7.55 (dd, *J* = 8.1, 7.6 Hz, 1H, ArH), 7.43 (ddd, *J* = 8.1, 2.4, 1.1 Hz, 1H, ArH), 7.14 (dd, *J* = 9.0, 8.5 Hz, 2H, ArH). ^13^C NMR (101 MHz, CDCl_3_) δ 191.11, 166.37 (d, *J* = 255.8 Hz), 163.92, 151.43, 137.89, 132.97, 132.87, 130.26, 127.67 (d, *J* = 37.1 Hz), 125.26 (d, *J* = 3.0 Hz), 122.40, 115.98 (d, *J* = 22.1 Hz). HRMS (ESI) calculated for C_14_H_9_FO_3_ [M]^+^ 244.05357, found 244.06335.

##### 4-Formyl-2,6-dimethoxyphenyl 4-Fluorobenzoate (ABMM-18)

White solid; 44% yield; mp 72–74 °C. ^1^H NMR (400 MHz, CDCl_3_) δ 9.88 (s, 1H, CHO), 8.18 (dd, *J* = 9.0, 5.4 Hz, 2H, ArH), 7.16–7.08 (m, 4H, ArH), 3.83 (s, 6H, OCH_3_). ^13^C NMR (101 MHz, CDCl_3_) δ 191.07, 166.27 (d, *J* = 255.2 Hz), 162.95, 153.13, 134.49, 133.90, 133.14 (d, *J* = 9.5 Hz), 125.14 (d, *J* = 3.0 Hz), 115.81 (d, *J* = 22.1 Hz), 106.14, 56.42. HRMS (ESI) calculated for C_16_H_13_FO_5_ [M + H]^+^ 305.07470, found 305.90508.

##### (*E*)-4-(3-Hydroxyprop-1-en-1-yl)-2-methoxyphenyl 4-Fluorobenzoate (ABMM-19)

White solid; 70% yield; mp 124–126 °C. ^1^H NMR (400 MHz, CDCl_3_) δ 8.16 (dd, *J* = 9.0, 5.4 Hz, 2H, ArH), 7.11 (t, *J* = 8.7 Hz, 2H, ArH), 7.03 (d, *J* = 8.1 Hz, 1H, ArH), 6.99–6.90 (m, 2H, ArH), 6.55 (d, *J* = 15.9 Hz, 1H, CH), 6.28 (dt, *J* = 15.9, 5.7 Hz, 1H, CH), 4.28 (td, *J* = 5.7, 1.4 Hz, 2H, CH_2_), 3.76 (s, 3H, OCH_3_), 1.41 (t, *J* = 5.9 Hz, 1H, OH). ^13^C NMR (101 MHz, CDCl_3_) δ 166.15 (d, *J* = 254.8 Hz), 163.81, 151.30, 139.43, 135.92, 132.99, 132.89, 129.73 (d, *J* = 156.2 Hz), 125.63 (d, *J* = 3.0 Hz), 122.94, 119.22, 115.74 (d, *J* = 22.0 Hz), 110.25, 63.62, 55.91. HRMS (ESI) calculated for C_17_H_15_FO_4_ [M + H]^+^ 303.09544, found 303.10255.

##### 4-(Hydroxymethyl)-2,6-dimethoxyphenyl 4-Fluorobenzoate (ABMM-23)

White solid; 68% yield; mp 105–107 °C. ^1^H NMR (400 MHz, DMSO) δ 8.84–8.21 (m, 4H, ArH), 7.85 (d, *J* = 7.7 Hz, 2H, ArH), 4.06 (d, *J* = 6.9 Hz, 2H, CH_2_), 2.10–1.63 (m, br, 1H, OH), ^13^C NMR (101 MHz, CDCl_3_) δ 168.20 (d, *J* = 256.1 Hz), 165.95, 152.13 (2C), 140.09, 133.90 (2C), 133.14 (d, *J* = 9.4 Hz), 125.14 (d, *J* = 3.0 Hz), 115.81 (d, *J* = 22.3 Hz, 2C), 106.14 (2C), 65.6, 55.72 (2C). HRMS (ESI) calculated for C_16_H_15_FO_4_ [M + NH_4_]^+^ 324.1242, found 324.1183.

##### (*E*)-3-(4-((4-Bromobenzyl)oxy)-3-methoxyphenyl)acrylaldehyde (ABMM-24)

Off-white solid; 44% yield; mp 98–100 °C. ^1^H NMR (400 MHz, DMSO) δ 9.62 (d, *J* = 7.8 Hz, 1H, CHO), 7.65 (d, J = 15.8 Hz, 1H, CH), 7.61 (d, J = 8.4 Hz, 2H, ArH), 7.43–7.39 (m, 3H, ArH), 7.28 (dd, *J* = 8.3, 1.8 Hz, 1H, ArH), 7.11 (d, *J* = 8.4 Hz, 1H, ArH), 6.83 (dd, *J* = 15.8, 7.8 Hz, 1H, CH), 5.16 (s, 2H, CH_2_), 3.84 (s, 3H, OCH_3_). ^13^C NMR (101 MHz, DMSO) δ 194.65, 153.95, 150.80, 149.80, 136.61, 131.87, 130.40, 127.85, 127.26, 123.95, 121.58, 113.70, 111.42, 69.48, 56.22. HRMS (ESI) calculated for C_17_H_15_BrO_3_ [M + H]^+^ 347.0205, found 347.0211.

##### (*E*)-3-(4-((4-Bromobenzyl)oxy)-3-methoxyphenyl)prop-2-en-1-ol (ABMM-25)

Pale yellow solid; 82% yield; mp 80–82 °C. ^1^H NMR (400 MHz, DMSO) δ 7.59 (d, *J* = 8.3 Hz, 2H, ArH), 7.40 (d, *J* = 8.3 Hz, 2H, ArH), 7.07 (s, 1H, ArH), 6.91 (dt, *J* = 8.3, 4.9 Hz, 2H, ArH), 6.47 (d, *J* = 15.9 Hz, 1H, CH), 6.27 (dt, *J* = 15.9, 5.2 Hz, 1H, CH), 5.06 (s, 2H, CH2), 4.82 (t, *J* = 5.4 Hz, 1H, OH), 4.10 (t, *J* = 4.6 Hz, 2H, CH_2_), 3.80 (s, 3H, OCH_3_). ^13^C NMR (101 MHz, DMSO) δ 149.73, 147.45, 137.14, 131.78, 130.96, 130.29, 129.33, 128.89, 121.37, 119.47, 114.22, 109.96, 69.58, 62.06, 56.00. HRMS (ESI) calculated for C_17_H_17_BrO_3_ [M + H]^+^ 349.0361, found 349.0451

##### (*E*)-3-(4-((4-Bromobenzyl)oxy)phenyl)acrylic Acid (ABMM-26)

Off-white solid; 61% yield; mp 80–82 °C. ^1^H NMR (300 MHz, DMSO) δ 7.58 (d, *J* = 15.9 Hz, 1H, CH), 7.44 (d, *J* = 8.3 Hz, 2H, ArH), 7.37–7.13 (m, 2H, ArH), 7.13–6.90 (m, 2H, ArH), 6.85 (d, *J* = 8.2 Hz, 2H, ArH), 6.25 (d, *J* = 15.9 Hz, 1H, CH), 5.79 (s, 2H, CH_2_). ^13^C NMR (75 MHz, DMSO) δ 166.91, 148.11, 146.76, 145.53, 135.23, 131.73, 129.91, 126.85, 123.22, 122.24, 114.93, 114.73, 109.33, 65.40, 55.94. HRMS (ESI) calculated for C_16_H_13_BrO_3_ [M + H]^+^ 333.0048, found 333.0288.

##### (*E*)-3-(4-((4-Fluorobenzoyl)oxy)phenyl)acrylic Acid (ABMM-27)

Off-white solid; 41% yield; mp 112–114 °C. ^1^H NMR (300 MHz) δ 8.16 (dd, J = 8.4, 5.5 Hz, 2H, ArH), 8.10–7.92 (m, 2H, ArH), 7.71 (d, J = 15.9 Hz, 1H, CH), 7.29–6.95 (m, 4H, ArH), 6.37 (d, J = 15.9 Hz, 1H, CH). ^13^C NMR (75 MHz, DMSO) δ 171.71, 170.53, 166.25 (d, J = 257.4 Hz), 151.65, 146.25, 141.91, 132.98 (d, J = 13.0 Hz), 123.45, 121.65, 117.59, 115.82 (d, J = 22.0 Hz), 111.54. HRMS (ESI) calculated for C_16_H_11_FO_4_ [M + H]^+^ 287.0641, found 287.0912.

##### 4-Formyl-2-methoxyphenyl 4-(Chloromethyl)benzoate (ABMM-28)

White solid; 48% yield; mp 93–95 °C. NMR (300 MHz) δ 9.93 (s, 1H, CHO), 8.15 (d, *J* = 8.1 Hz, 2H, ArH), 7.79–7.12 (m, 5H, ArH), 4.60 (s, 2H, CH_2_), 3.83 (s, 3H, OCH_3_). NMR (75 MHz) δ 191.07, 163.64, 152.16, 145.11, 143.26, 135.35, 131.00, 130.84, 128.97, 128.80, 128.74, 124.78, 123.54, 110.92, 56.14, 45.26. HRMS (ESI) calculated for C_16_H_13_ClO_4_ [M + H]^+^ 304.0502, found 304.0584.

##### 4-Formylphenyl 4-(Chloromethyl)benzoate (ABMM-32)

White solid; 58% yield; mp 90–92 °C. NMR (300 MHz, CDCl_3_) δ 10.05 (s, CHO), 8.22 (d, *J* = 8.2 Hz, 2H, ArH), 8.00 (d, *J* = 8.5 Hz, 2H, ArH), 7.58 (d, *J* = 8.2 Hz, 2H, ArH), 7.44 (d, *J* = 8.5 Hz, 2H, ArH), 4.68 (s, 2H, CH_2_). NMR (75 MHz, CDCl_3_) δ 190.91, 164.00, 155.56, 143.48, 134.16, 131.30, 130.73, 128.80, 122.51, 45.19. HRMS (ESI) calculated for C_15_H_11_ClO_3_ [M + H]^+^ 275.0397, found 275.0444.

##### (*E*)-2-Methoxy-4-(3-oxoprop-1-en-1-yl)phenyl 4-Nitrobenzoate (ABMM-33)

Yellow solid; 48% yield; mp 125–127 °C. NMR (300 MHz, DMSO) δ 9.67 (d, *J* = 6.9 Hz, CHO), 8.62–8.08 (m, 4H, ArH), 7.43 (d, *J* = 15.5 Hz, 1H, CH), 7.25–6.92 (m, 3H, ArH), 6.65 (dd, *J* = 15.5, 6.9 Hz, 1H, CH), 3.82 (s, 3H, OCH_3_). NMR (75 MHz, DMSO) δ 193.35, 162.57, 151.54, 151.00, 141.85, 134.41, 133.49, 131.49, 129.06, 123.74, 123.37, 121.88, 111.54, 56.04. HRMS (ESI) calculated for C_17_H_14_NO_6_ [M + H]^+^ 328.0743, found 328.0822.

##### 2-Formylphenyl 4-Chlorobenzoate (ABMM-34)

White solid; 74% yield; mp 85–87 °C. NMR (300 MHz, CDCl_3_) δ 10.11 (s, 1H, CHO), 8.11 (d, *J* = 8.2 Hz, 2H, ArH), 8.02 (d, *J* = 8.3 Hz, 1H, ArH), 7.89 (d, *J* = 7.6 Hz, 1H, ArH), 7.68–7.58 (m, 1H, ArH), 7.46 (d, *J* = 8.2 Hz, 2H, ArH), 7.26 (d, *J* = 8.0 Hz, 1H, ArH). NMR (75 MHz, CDCl_3_) δ 188.44, 135.39, 131.88, 131.72, 131.66, 130.94, 129.38, 129.17, 128.91, 126.67, 126.33, 124.04, 123.52. HRMS (ESI) calculated for C_14_H_9_ClO_3_ [M + H]^+^ 261.0240, found 261.0316.

##### 4-(Hydroxymethyl)phenyl 4-Chlorobenzoate (ABMM-35)

White solid; 64% yield; mp 93–95 °C. NMR (300 MHz, CDCl_3_) δ 8.08 (d, *J* = 7.3 Hz, 2H, ArH), 7.41 (m, 4H, ArH), 7.14 (d, *J* = 7.3 Hz, 2H, ArH), 4.67 (s, 2H, CH_2_), 1.64 (s, 1H, OH). NMR (75 MHz, CDCl_3_) δ 164.40, 150.18, 140.20, 138.72, 131.56, 128.97, 128.19, 127.94, 121.74, 64.79. HRMS (ESI) calculated for C_14_H_11_ClO_3_ [M + H]^+^ 263.0397, found 263.0475.

### 3.2. Purification of Recombinant Human ALDHs and Enzymatic Assays

Human ALDH1A1 and ALDH1A3 were cloned, recombinantly expressed and affinity-purified as previously described [30]. Human ALDH3A1 was also cloned and recombinantly expressed from the pET-30 Xa/LIC vector and purified using the same procedure. Enzymes including the N-terminal (His)_6_ tag were stored at −20 °C in 20 mM Tris/HCl, 0.5 M NaCl, pH 8.0, and 5 mM DTT until use. Activity under standard conditions was measured fluorimetrically to follow the purification procedure and to check the enzyme concentration before each kinetic experiment. Standard activity of each isoform was measured using specific reaction buffers and at saturating concentrations of substrate, as described below.

### 3.3. Inhibition Screening

Single-point measurements of enzymatic activity at 10 µM inhibitor were performed at 25 °C using a Cary Eclipse (Varian) fluorimeter. The dehydrogenase activity was monitored by following the NAD(P)H fluorescence, by sample excitation at 340 nm and fluorescence emission at 460 nm. The enzymatic reaction was initiated by the addition of the substrate to a 1 mL quartz cuvette containing the enzyme and the cofactor. The reaction mixture also contained 5 µM of NAD(P)H as an internal standard to obtain absolute reaction rates, as described [30,49]. Assays without enzyme were always carried out as a control to ensure the absence of activity. The reaction buffer was 50 mM HEPES, 0.5 mM EDTA and 0.5 mM DTT, pH 8.0, for ALDH1A1; 50 mM HEPES, 50 mM MgCl_2_ and 5 mM DTT, pH 8.0, for ALDH1A3; and 50 mM Tris-HCl and 5 mM DTT, pH 8.0, for ALDH3A1. Hexanal was used as the standard substrate for ALDH1A1 and ALDH1A3, while 4-nitrobenzaldehyde (4-NBA) was used as the substrate for ALDH3A1. The assay was typically performed at saturating substrate concentrations (30 µM hexanal for ALDH1A1, 250 µM hexanal for ALDH1A3 and 250 µM 4-NBA for ALDH3A1). ALDH1A3 activity was also measured at a substrate concentration near the *K_m_* value (10 µM hexanal). All substrates were prepared at a concentration of 2 mM and further diluted to reach the final concentrations required per experiment. All tested compounds were dissolved in DMSO and assayed at a final concentration of 1% (*v*/*v*) DMSO. In all assays, the reaction took place in a final volume of 1 mL and in the presence of 0.5 mM NAD^+^ for ALDH1A1/ALDH1A3 or 1 mM NADP^+^ for ALDH3A1, and 10 µM inhibitor or 1% DMSO for the control. The concentration of enzyme was kept from 50- to 100-fold lower than that of the substrate for all enzymatic assays. The reaction mixture was pre-incubated for 5 min at room temperature (except for the slow-binding assay where the mixture was pre-incubated for 20 min) before the substrate was added to initiate the reaction. All assays were performed as duplicates and the results are expressed as the mean, with a relative error always lower than 30%. The results are presented as the percentage of remaining activity, which is calculated as the ratio of activity in presence of inhibitor versus that of the control with 1% DMSO.

### 3.4. Determination of Half-Maximal Inhibitory Concentrations (IC_50_)

IC_50_ values of enzymatic activity were determined for the two best inhibitor candidates against ALDH1A3. Reaction rates were determined at various inhibitor concentrations at a fixed substrate concentration. The IC_50_ values were calculated by a non-linear fitting of the experimental data to a sigmoidal plot using GraFit 5.0 (Erithacus software), with the following 4-parameter equation: y = $\frac{\mathrm{range}}{1+{\left(\frac{x}{{\mathrm{IC}}_{50}}\right)}^{s}}+$ *background*, where y is the specific activity, x is the inhibitor concentration, *background* is the minimum y value, range is the fitted uninhibited value minus the background, and s is a slope factor. Reaction conditions were the same as those described in Section 3.3, and 250 µM hexanal was used as the substrate.

### 3.5. Biology

#### 3.5.1. Cell Culture

A549 and H1299 human NSCLC cell lines were cultured in RPMI-1640 medium, supplemented with 10% (*v*/*v*) heat inactivated fetal bovine serum and (2 mmol/L) l-glutamine. The cells were maintained in 5% CO_2_ humidified incubator at 37 °C.

#### 3.5.2. Cytotoxicity Experiment

A549 and H1299 cells were seeded at 750 and 1000 cells per well, respectively, into 96-well plates and left overnight to allow cell attachment. Cells were then treated with different compounds at various concentrations ranging between 0.01 and 60 µM for 24 h or 96 h. For the shorter exposure period, the drug-containing medium was removed after 24 h and replenished with fresh growth medium for the remainder of the 96 h period. The number of surviving cells was then determined using MTT (3-(4,5-dimethylthiazole-2-yl)-2,5-diphenyltetrazolium bromide) Cell Viability Assays, according to the Assay Guidance Manual [50]. MTT solution (2.5 mg/mL) was prepared in sterile PBS. Following 93 h of incubation, 50 µL MTT solution was added to the cells and incubated for 3 h at 37 °C. The medium was then removed and the remaining formazan crystals were dissolved in 200 µL DMSO (TEDIA, USA). Optical densities were then measured on a SynergyHTX^®^ spectrophotometer at 540 nm and then analyzed using Gen5 Software package. The results were then used to calculate the surviving fraction of cells relative to solvent-only controls. Dose–response curves were generated using the GraphPad Prism 7 Software, and nonlinear regression analysis was used to fit the data. IC_50_ values (defined as the concentration of drug required to decrease cell survival by 50% relative to controls) were determined from these curves.

### 3.6. In Silico Study

#### 3.6.1. Protein and Ligand Preparation

The X-ray structure of human ALDH1A1 in complex with 1-{[1,3-dimethyl-7-(3-methylbutyl)-2,6-dioxo-2,3,6,7-tetrahydro-1H-purin-8-yl]methyl}piperidine-4-carboxamide (PDB ID: 4WPN), human ALDH1A3 in complex with RA and NAD+ (PDB ID: 5FHZ) and human ALDH3A1 in complex with selective inhibitor N-[4-(4-methylsulfonyl-2-nitroanilino)phenyl]acetamide (PDB ID: 4H80) were downloaded from the Protein Data Bank [29,51,52]. The resolution of the X-ray structures was found to be 1.95, 2.90, and 2.50 Å for ALDH1A1, ALDH1A3 and ALDH3A1, respectively. All the structures were primarily selected, after satisfying the X-ray diffraction resolution of not more than 3.0 Å (higher values are associated with poor quality). Furthermore, the X-ray structures for ALDH1A1 and ALDH3A1 were selected based on the presence of a selective co-crystallized inhibitor, which should have a reasonable inhibitory effect (IC_50_, K_i_) and should demonstrate a significant no. of H-bond and hydrophobic contacts within the binding site, which could be exploited to compare with the synthesized analogues. The X-ray structure of human ALDH1A3 is co-crystallized with the reaction product retinoic acid (RA), which forms H-bond interactions with the active site C313/C314/T315 residues, whereas other available X-crystal structures of ALDH1A3, which have co-crystallized inhibitors, do not exemplify the H-bond interactions with these catalytic residues, and thus the X-ray structure of ALDH1A3 with PDB ID, 5FHZ, was chosen in our study. The co-crystallized ligands, ions and water molecules were removed from the X-ray complexes and H-bonds, and missing residues were added to the protein with the aid of the protein preparation wizard of Maestro. All the compounds were drawn using the Build panel of Maestro and subjected to LigPrep tool interfaced with the Maestro module of Schrödinger suite. The 3D structures, including all possible tautomers and ionization states at pH 7.0 ± 2.0 of all the compounds, were generated and geometrically minimized using OPLS3e force field.

#### 3.6.2. Molecular Docking

All docking calculations were carried out on the above-mentioned X-ray structures of ALDH1A1, ALDHA3 and ALDH3A1. Glide 5.0 with standard precision (SP) method [53] was used for the docking of all compounds.

GLIDE 5.0. The binding site was defined by a rectangular box of 10 Å in the x, y, and z directions centered on the ligand. The GLIDE defaults were used for all parameter settings. The GlideScore fitness function is based on Chemscore but includes a steric-clash term, adds buried polar terms to penalize electrostatic mismatches and involves modifications on other secondary terms. The docking analyses were carried out using the Standard Precision (SP) method. A total of 50 docking solutions were generated for each ligand, and the top-ranked docking pose was considered the final pose.

The reliability of the docking program: Glide 5.0 was assessed by performing self-docking analysis and calculating the root-mean-square deviation (RMSD) between the crystallographic position of the ligand and the ligand’s disposition predicted by docking. The rms_analysis program of Gold suite was used to calculate the RMSD difference, considering only the heavy atoms of the ligand. The docking method is able to produce a binding pose within 2.0 Å RMSD of the crystallographic disposition; therefore, it is considered reliable [54].

#### 3.6.3. Visualization of the Docked Poses

Two-dimensional and three-dimensional images of all the docked poses were generated by using Discovery Studio Client 2021.

## 4. Conclusions

In summary, we implemented several experiments to prove that ABMM-15 and ABMM-16, which are based on a benzyloxybenzaldehyde scaffold, act as selective ALDH1A3 inhibitors. For this purpose, these two compounds were evaluated against two other isoforms (ALDH1A1 and ALDH3A1) and showed no significant inhibition of their activities. Other isoforms, such as ALDH1A2 and ALDH2, might be of interest to be kinetically assayed using ABMM-15 and ABMM-16 to better assert their selective inhibition of ALDH1A3. The IC_50_ values for these two compounds on ALDH1A3 were promising, which could potentially be considered for therapeutic intervention via further compound optimization. Accordingly, both ABMM-15 and ABMM-16 were evaluated alone in a panel of two NSCLC cell lines (including ALDH-positive A549 and ALDH-negative H1299 cells), and toxicity was clearly absent at the maximum concentration of 60 µM. However, these results suggest that ABMM-15 and ABMM-16 can be used in a wide range of nontoxic concentrations, in which they may have a potential role in diminishing the chemoresistance of certain anticancer drugs when used in combination therapy. Hence, further research on investigating their cytotoxicity alone and in combination with anticancer drugs in drug-resistant cancer cell lines, particularly those expressing elevated levels of the ALDH1A3 isoform, is worth pursuing. In addition, more analogues for ABMM-15 can also be designed, synthesized and evaluated for ALDH isoform selectivity and in a panel of cancer cell lines to obtain a broader conclusive SAR study.

The synthesized coniferyl aldehyde analogues were also found to be interesting regarding their enhanced cytotoxicity on H1299 cell lines, but with insignificant ALDH inhibition. Therefore, the mechanistic pathway of their selective toxicity remains unknown and warrants further investigation.

## Figures and Tables

**Table 1:** Inhibition screening of the selected ABMM compounds against ALDH1A1, ALDH1A3 and ALDH3A1. Enzyme activity was measured with hexanal (ALDH1A1 and ALDH1A3) or 4-NBA (ALDH3A1). Experimental values are shown as the mean ± standard error (SE). ND: Not Determined.

	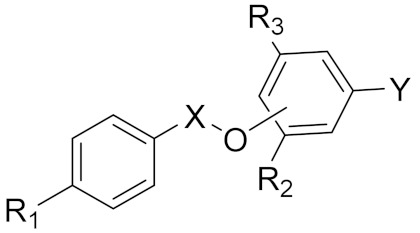					Remaining Activity ± SE (%) at 10 µM Compound			
						ALDH1A1	ALDH1A3		ALDH3A1
	R_1_	R_2_	R_3_	X	Y	[S] at Saturation (30 µM Hex)	[S] near Km (10 µM Hex)	[S] at Saturation (250 µM Hex)	[S] at Saturation (250 µM 4-NBA)
ABMM-1	Cl	OCH_3_	H	C=O	CHO	81.93 ± 2.9	21.07 ± 0.1	61.14 ± 2.6	ND
ABMM-2	Cl	H	H	C=O	CHO	56.98 ± 5.8	94.76 ± 8.3	79.70 ± 1.9	162.08 ± 10.7
ABMM-5	NO_2_	OCH_3_	H	C=O	CHO	92.38 ± 4.6	61.93 ± 5.8	79.78 ± 5.9	ND
ABMM-6	F	OCH_3_	H	C=O	CH=CHCHO	94.76 ± 8.3	76.26 ± 3.5	91.27 ± 1.8	92.54 ± 11.4
ABMM-15	Cl	H	H	CH_2_	CHO	48.22 ± 2.8	0.14 ± 0.1	12.35 ± 0.7	146.35 ± 2.7
ABMM-16	Cl	OCH_3_	H	CH_2_	CHO	42.27 ± 1.7	4.27 ± 1.8	19.22 ± 0.5	117.43 ± 3.5
ABMM-17 *	Cl	H	---	C=O	CHO	57.85 ± 5.5	113.0 ± 0.5	95.06 ± 1.9	104.43 ± 2.4
ABMM-18	F	OCH_3_	OCH_3_	C=O	CHO	70.86 ± 2.6	16.01 ± 3.4	77.30 ± 9.9	96.69 ± 3.3
ABMM-19	F	OCH_3_	H	C=O	CH=CHCH_2_OH	89.13 ± 0.4	88.32 ± 0.7	77.91 ± 7.4	124.32 ± 9.3

* Compound ABMM-17 has the oxygen at a position *meta* to Y; thus, no R_3_ group exists.

**Table 2:** Results of the inhibition screening after 5 and 20 min of pre-incubation for ALDH1A3 at 250 µM hexanal.

ALDH1A3 Remaining Activity ± SE (%) at 10 µM Compound		
Pre-Incubation	5 min	20 min
ABMM-15	12.35 ± 0.72	13.10 ± 4.35
ABMM-16	19.22 ± 0.51	18.88 ± 1.81

**Table 3:**
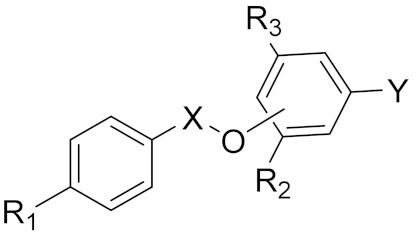
The chemical composition of ABMM analogues with IC_50_ values against A549 and H1299 cells (following 96 h of exposure) and against the ALDH1A3 enzyme. ND: Not Determined.

Compound	(O) Position Relative To (Y)	R1	R2	R3	X	Y	Toxicity (IC_50_ (µM) ± SE)		IC_50_ (µM) ± SE ALDH1A3
							A549	H1299	
ABMM-1	*para*	Cl	OCH_3_	H	C=O	CHO	>60	>60	ND
ABMM-2	*para*	Cl	H	H	C=O	CHO	>60	>60	ND
ABMM-3	*para*	F	H	H	C=O	CHO	>60	>60	ND
ABMM-4	*para*	NO_2_	H	H	C=O	CHO	ND	ND	ND
ABMM-5	*para*	NO_2_	OCH_3_	H	C=O	CHO	ND	ND	ND
ABMM-6	*para*	F	OCH_3_	H	C=O	CH=CHCHO	>60	14 ± 2	ND
ABMM-15	*para*	Cl	H	H	CH_2_	CHO	>60	>60	0.23 ± 0.05
ABMM-16	*para*	Cl	OCH_3_	H	CH_2_	CHO	>60	>60	1.29 ± 0.10
ABMM-17	*meta*	Cl	H	---	C=O	CHO	>60	56 ± 2.65	ND
ABMM-18	*para*	F	OCH_3_	OCH_3_	C=O	CHO	>60	>60	ND
ABMM-19	*para*	F	OCH_3_	H	C=O	CH=CHCH_2_OH	>60	46.33 ± 4.48	ND
ABMM-23	*para*	F	OCH_3_	OCH_3_	C=O	CH2OH	51 ± 8	>60	ND
ABMM-24	*para*	Br	OCH_3_	H	CH_2_	CH=CHCHO	>60	13.67 ± 1.67	ND
ABMM-25	*para*	Br	OCH_3_	H	CH_2_	CH=CHCH_2_OH	>60	>60	ND
ABMM-26	*para*	Br	OCH_3_	H	CH_2_	CH=CHCOOH	>60	>60	ND
ABMM-27	*para*	F	OCH_3_	H	C=O	CH=CHCOOH	>60	>60	ND
ABMM-28	*para*	CH_2_Cl	OCH_3_	H	C=O	CHO	50 ± 1	37.5 ± 1.5	ND
ABMM-32	*para*	CH_2_Cl	H	H	C=O	CHO	51 ± 5	13 ± 2.08	ND
ABMM-33	*para*	NO_2_	OCH_3_	H	C=O	CH=CHCHO	>60	35.33 ± 1.76	ND
ABMM-34	*ortho*	Cl	H	H	C=O	CHO	>60	>60	ND
ABMM-35	*para*	Cl	H	H	C=O	CH_2_OH	>60	>60	ND

**Table 4:** Docking scores (kcal/mol) for compounds ABMM-1, 2, 15, 16 and 18, against the three ALDH isoforms (ALDH1A1, 1A3 and 3A1).

	Docking Scores (kcal/mol)		
	ALDH1A1	ALDH1A3	ALDH3A1
ABMM-1	−7.709	−7.717	−7.238
ABMM-2	−7.104	−7.844	−5.826
ABMM-15	−7.246	−7.804	−6.707
ABMM-16	−8.157	−8.306	−7.029
ABMM-18	−6.193	−7.753	−7.402

## Data Availability

Data is contained within the article and in the Supplementary Materials.

## Supplementary Materials

^1^H-NMR, ^13^C-NMR and HRMS for the synthesized compounds can be found in the Supplementary Materials.

## Abbreviations

ALDH, aldehyde dehydrogenase; NAD^+^, nicotinamide adenine dinucleotide; NADH, nicotinamide adenine dinucleotide reduced form; RA, retinoic acid; DEAB, diethylamino benzaldehyde; SCs, stem cells; CSCs, cancer stem cells; TLC, thin layer chromatography; HRMS, high resolution mass spectrometry; 4-NBA, 4-nitrobenzaldehyde; 1H-NMR, proton nuclear magnetic resonance; 13C-NMR, carbon-13 nuclear magnetic resonance; DMSO, dimethylsulfoxide; Hz, hertz; MHz, megahertz; DMF, dimethylformamide; Rf, retention factor; mp, melting point; RMSD, root-mean-square deviation; IC_50_, half-maximal inhibitory concentration; PDB, protein data bank; NSCLC, non-small cell lung cancer; MTT, (3-(4,5-dimethylthiazole-2-yl)-2,5-diphenyltetrazolium bromide); PBS, phosphate-buffered saline.
