# Supplementary material for: Design, Synthesis, Biological Evaluation and In Silico Study of Benzyloxybenzaldehyde Derivatives as Selective ALDH1A3 Inhibitors
Source: Molecules. 2021 Sep 23;26(19):5770. doi: 10.3390/molecules26195770 (PMC8510124; doi:10.3390/molecules26195770)
Supplement: Supplementary file 1 [file molecules-26-05770-s001.zip › HRMS Supplementary Data.pdf]

ABnn1

260221_ABnn1_HRMS #6-13 RT: 0.05-0.10 AV: 8 NL: 1.34E7
T: FTMS + p ESI Full ms [65.00-2000.00]

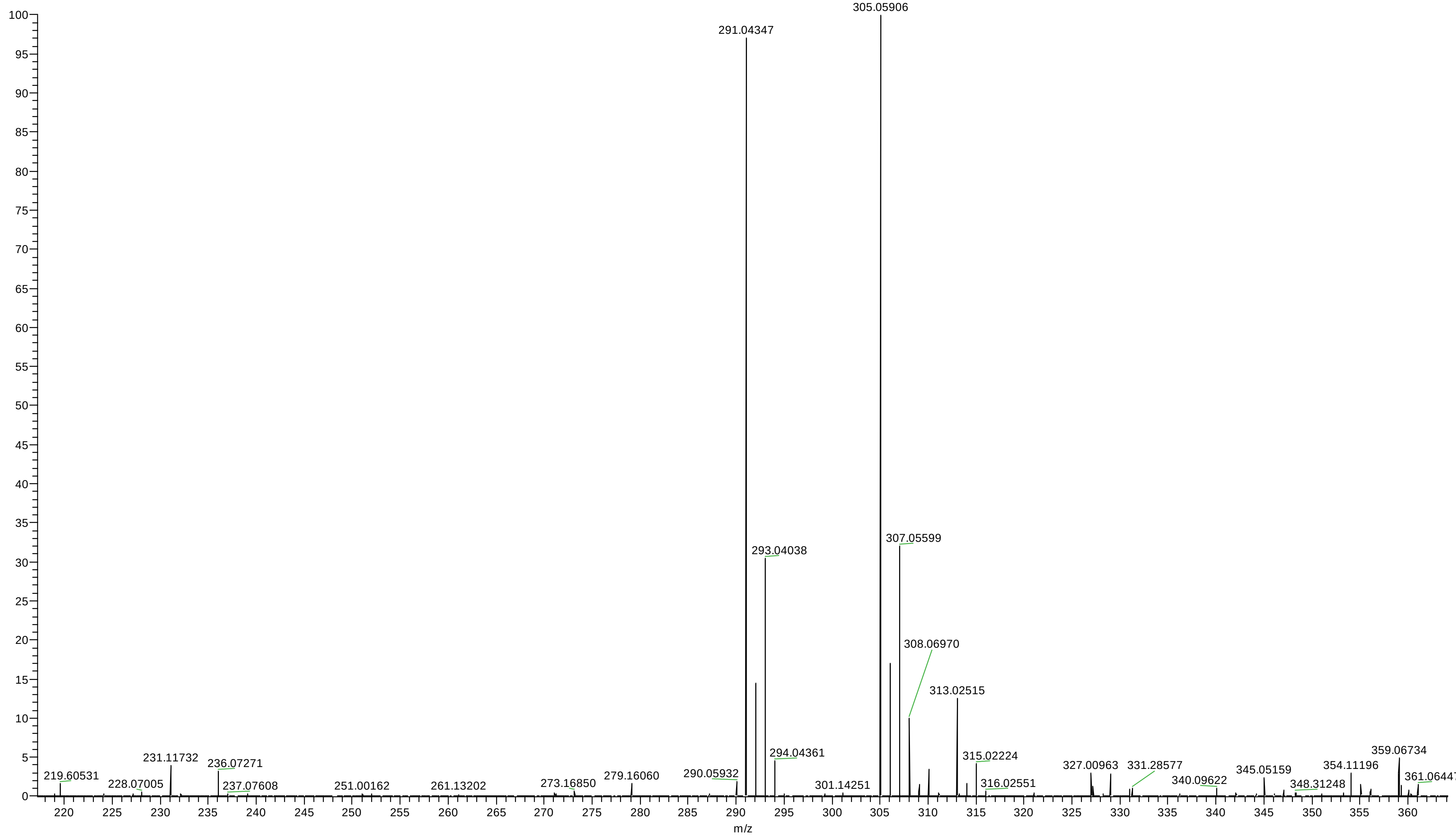

ABMM-2

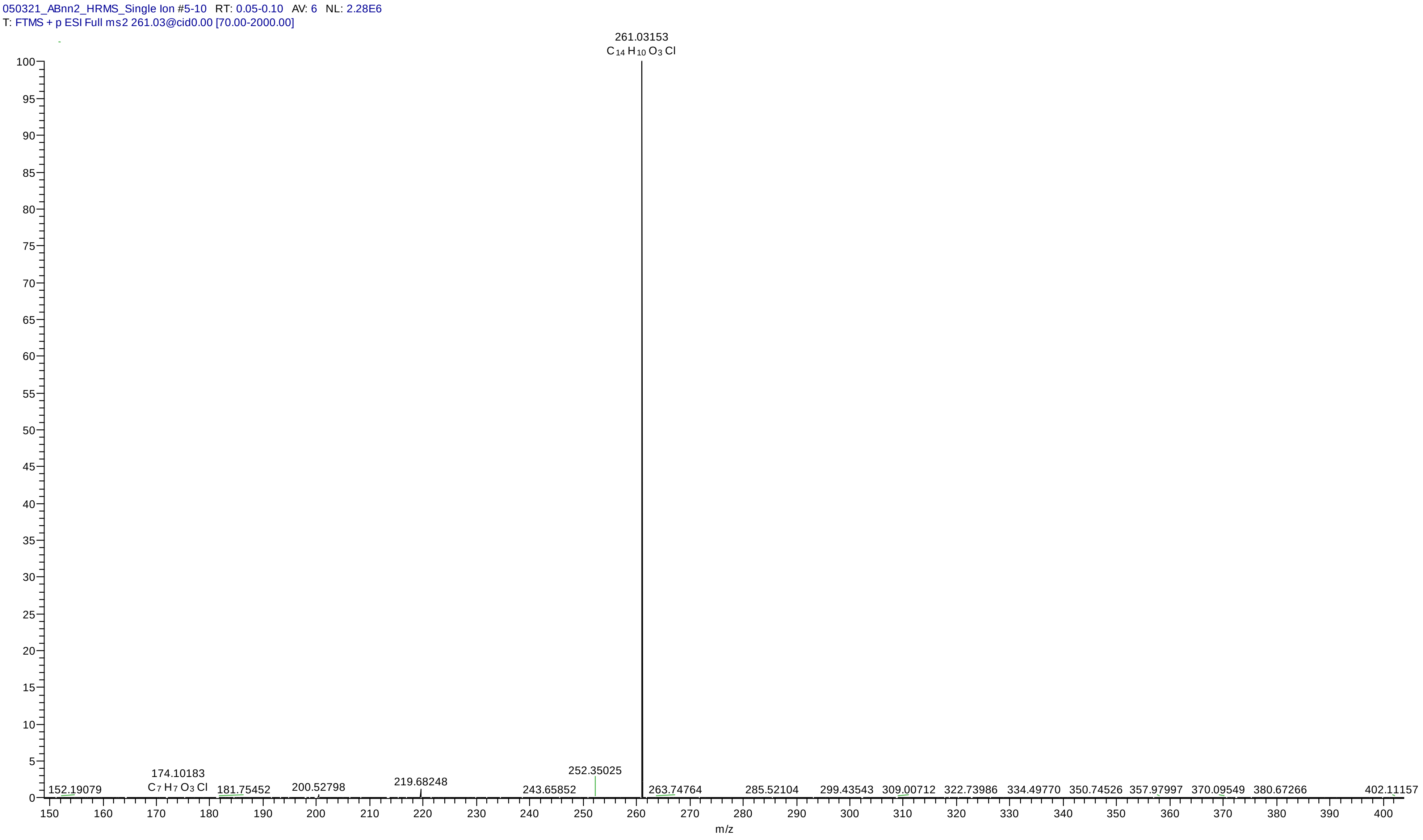

050321_ABnn2_HRMS_Single Ion #5-10 RT: 0.05-0.10 AV: 6 NL: 2.28E6
T: FTMS + p ESI Full ms2 261.03@cid0.00 [70.00-2000.00]
261.03153
C 14 H 10 O 3 Cl
174.10183
C 7 H 7 O 3 Cl
152.19079
181.75452
200.52798
219.68248
243.65852
252.35025
263.74764
285.52104
299.43543
309.00712
322.73986
334.49770
350.74526
357.97997
370.09549
380.67266
402.11157
m/z

ABMM-4

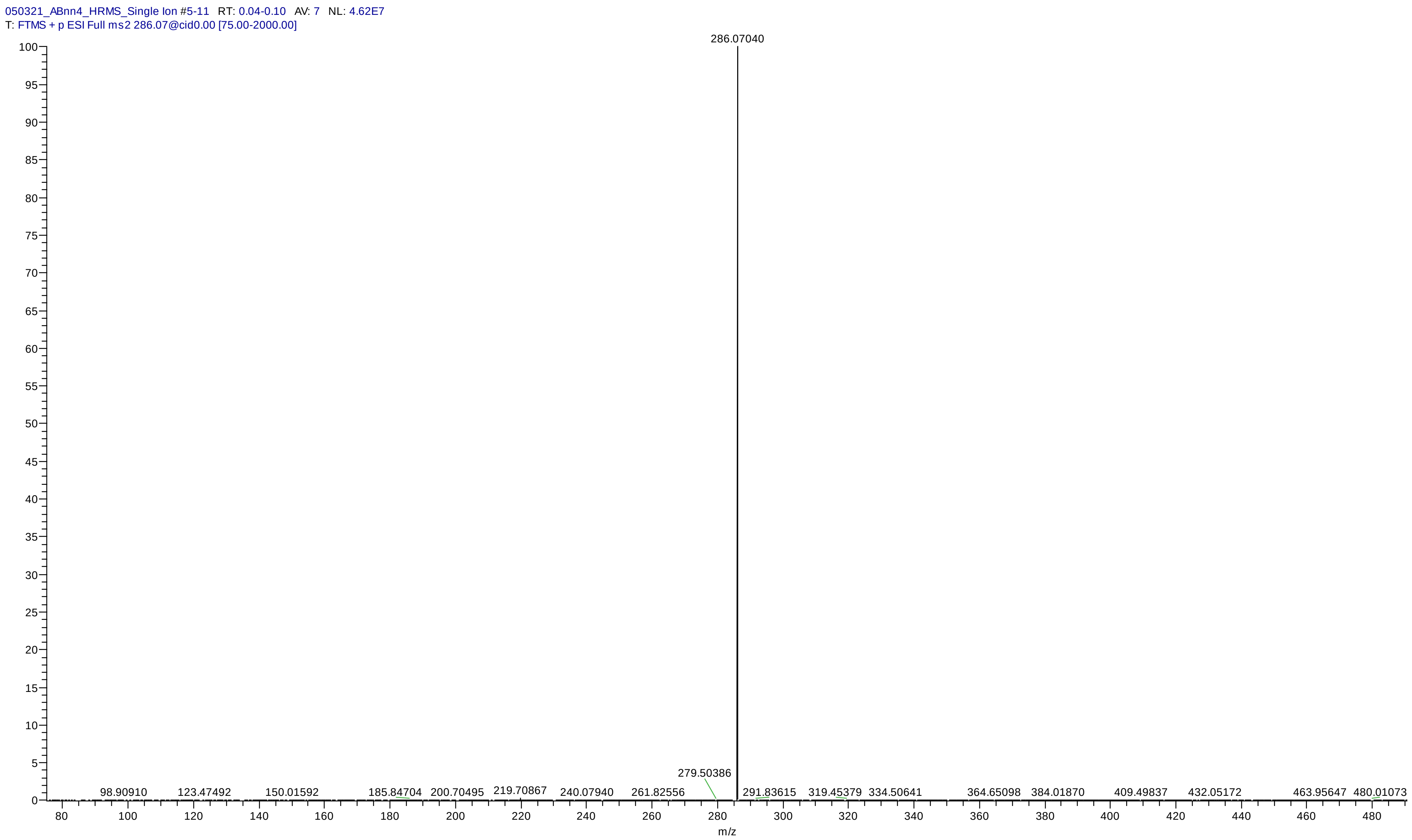

050321_ABnn4_HRMS_Single Ion #5-11 RT: 0.04-0.10 AV: 7 NL: 4.62E7
T: FTMS + p ESI Full ms2 286.07@cid0.00 [75.00-2000.00]
286.07040
279.50386
98.90910
123.47492
150.01592
185.84704
200.70495
219.70867
240.07940
261.82556
291.83615
319.45379
334.50641
364.65098
384.01870
409.49837
432.05172
463.95647
480.01073
m/z

ABMM-6

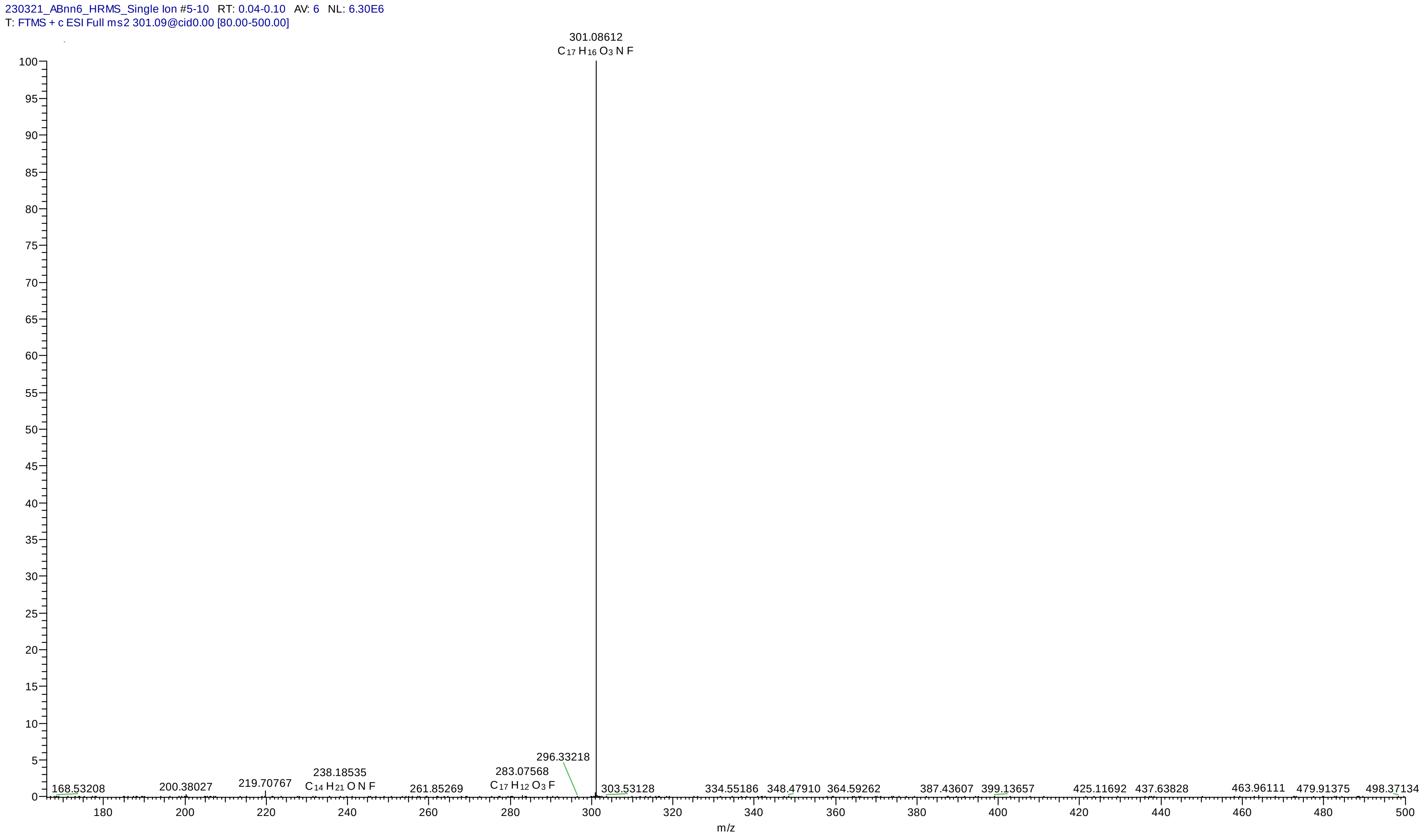
230321_ABnn6_HRMS_Single Ion #5-10 RT: 0.04-0.10 AV: 6 NL: 6.30E6
T: FTMS + c ESI Full ms2 301.09@cid0.00 [80.00-500.00]
301.08612
C17 H16 O3 N F
168.53208
200.38027
219.70767
238.18535
C14 H21 O N F
261.85269
283.07568
C17 H12 O3 F
296.33218
303.53128
334.55186
348.47910
364.59262
387.43607
399.13657
425.11692
437.63828
463.96111
479.91375
498.37134
m/z

ABMM-15

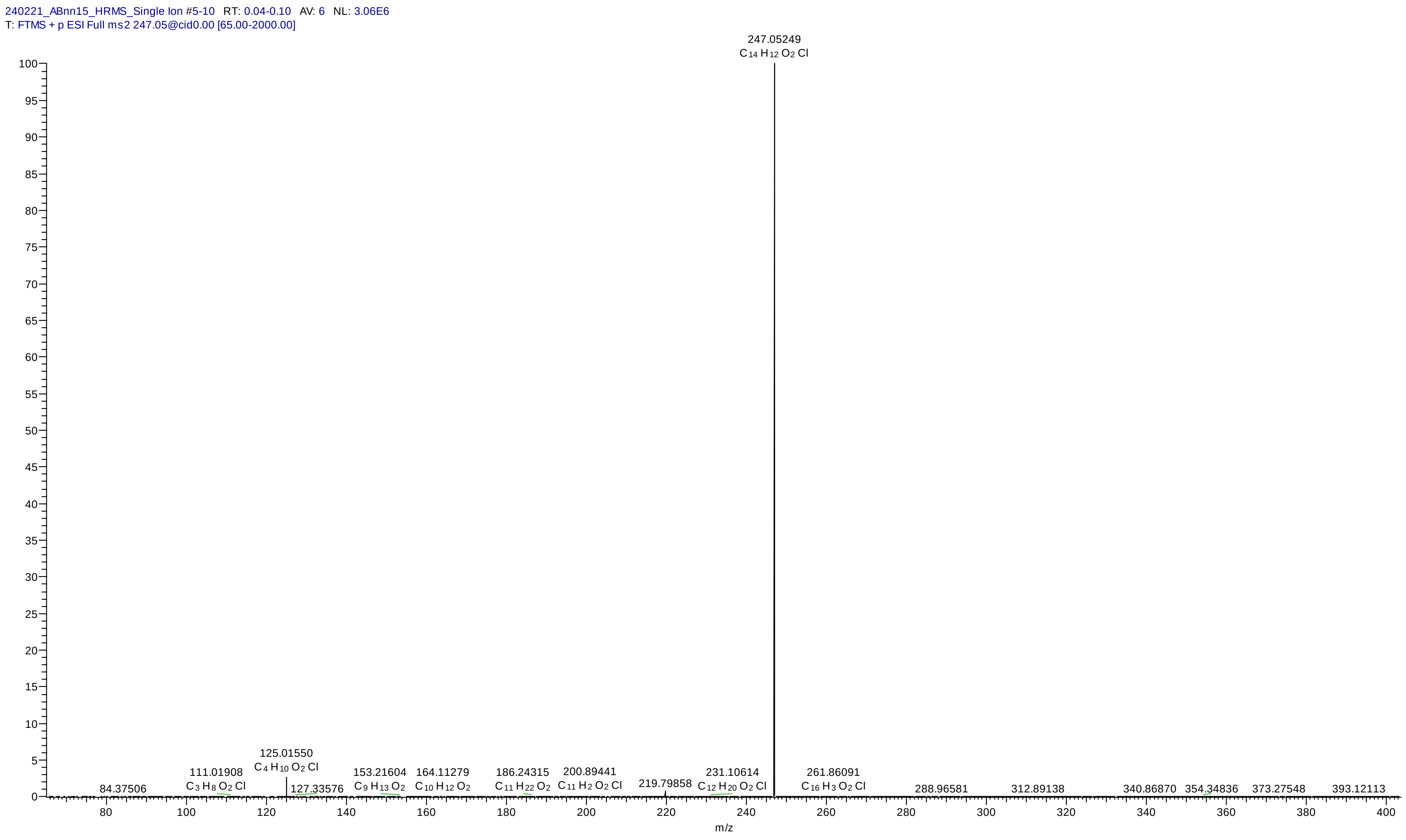
240221_ABnn15_HRMS_Single Ion #5-10 RT: 0.04-0.10 AV: 6 NL: 3.06E6
T: FTMS + p ESI Full ms2 247.05@cid0.00 [65.00-2000.00]
247.05249
C 14 H 12 O 2 Cl
125.01550
C 4 H 10 O 2 Cl
111.01908
C 3 H 8 O 2 Cl
84.37506
127.33576
153.21604
C 9 H 13 O 2
164.11279
C 10 H 12 O 2
186.24315
C 11 H 22 O 2
200.89441
C 11 H 2 O 2 Cl
219.79858
231.10614
C 12 H 20 O 2 Cl
261.86091
C 16 H 3 O 2 Cl
288.96581
312.89138
340.86870
354.34836
373.27548
393.12113
m/z

ABMM-16

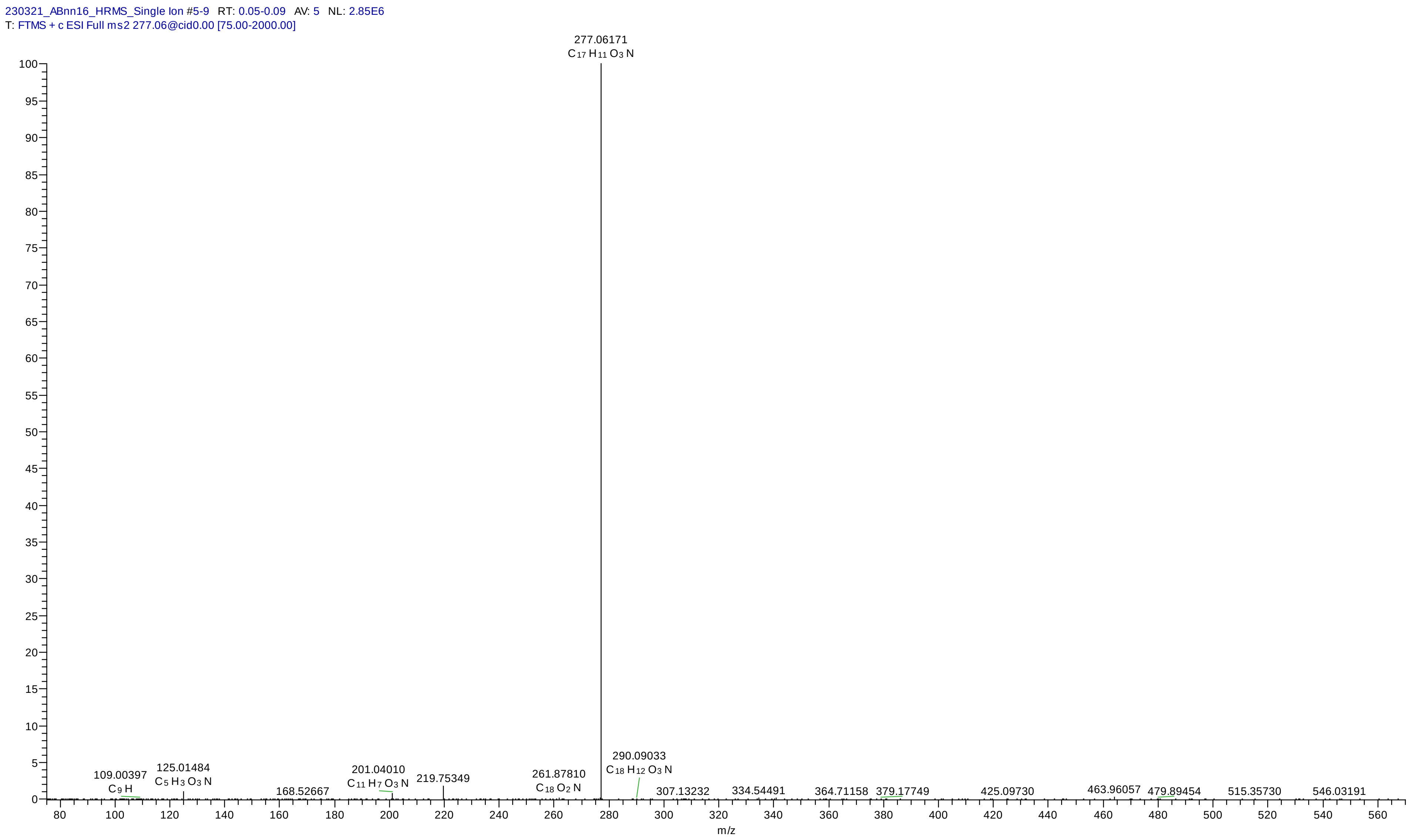

230321_ABnn16_HRMS_Single Ion #5-9 RT: 0.05-0.09 AV: 5 NL: 2.85E6
T: FTMS + c ESI Full ms2 277.06@cid0.00 [75.00-2000.00]
277.06171
$C_{17}H_{11}O_3N$
109.00397
C_9H
125.01484
$C_5H_3O_3N$
168.52667
201.04010
$C_{11}H_7O_3N$
219.75349
261.87810
$C_{18}O_2N$
290.09033
$C_{18}H_{12}O_3N$
307.13232
334.54491
364.71158
379.17749
425.09730
463.96057
479.89454
515.35730
546.03191
m/z

ABMM-17

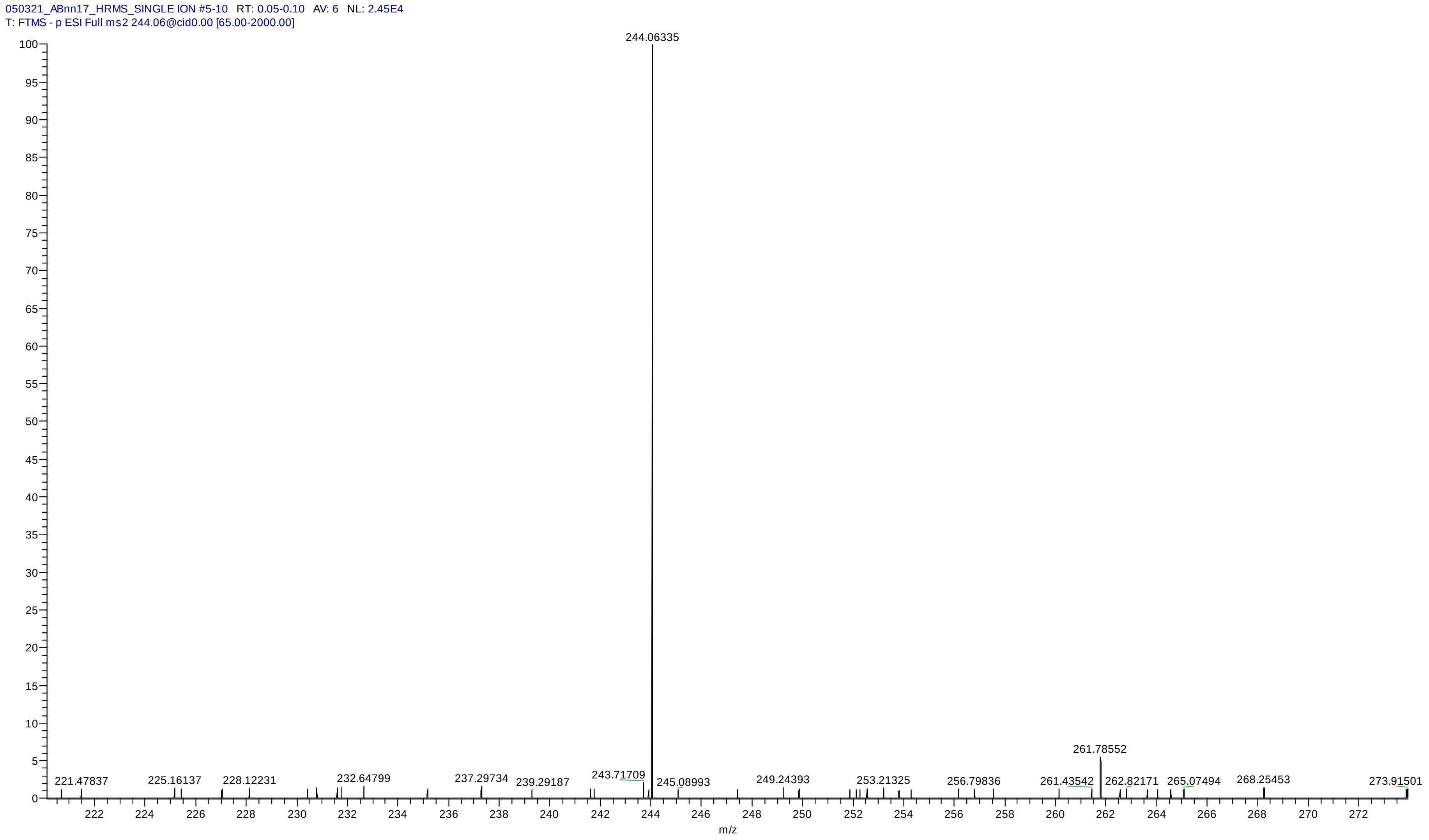
050321_ABnn17_HRMS_SINGLE ION #5-10 RT: 0.05-0.10 AV: 6 NL: 2.45E4
T: FTMS - p ESI Full ms2 244.06@cid0.00 [65.00-2000.00]
244.06335
261.78552
221.47837
225.16137
228.12231
232.64799
237.29734
239.29187
243.71709
245.08993
249.24393
253.21325
256.79836
261.43542
262.82171
265.07494
268.25453
273.91501
m/z

ABMM-18

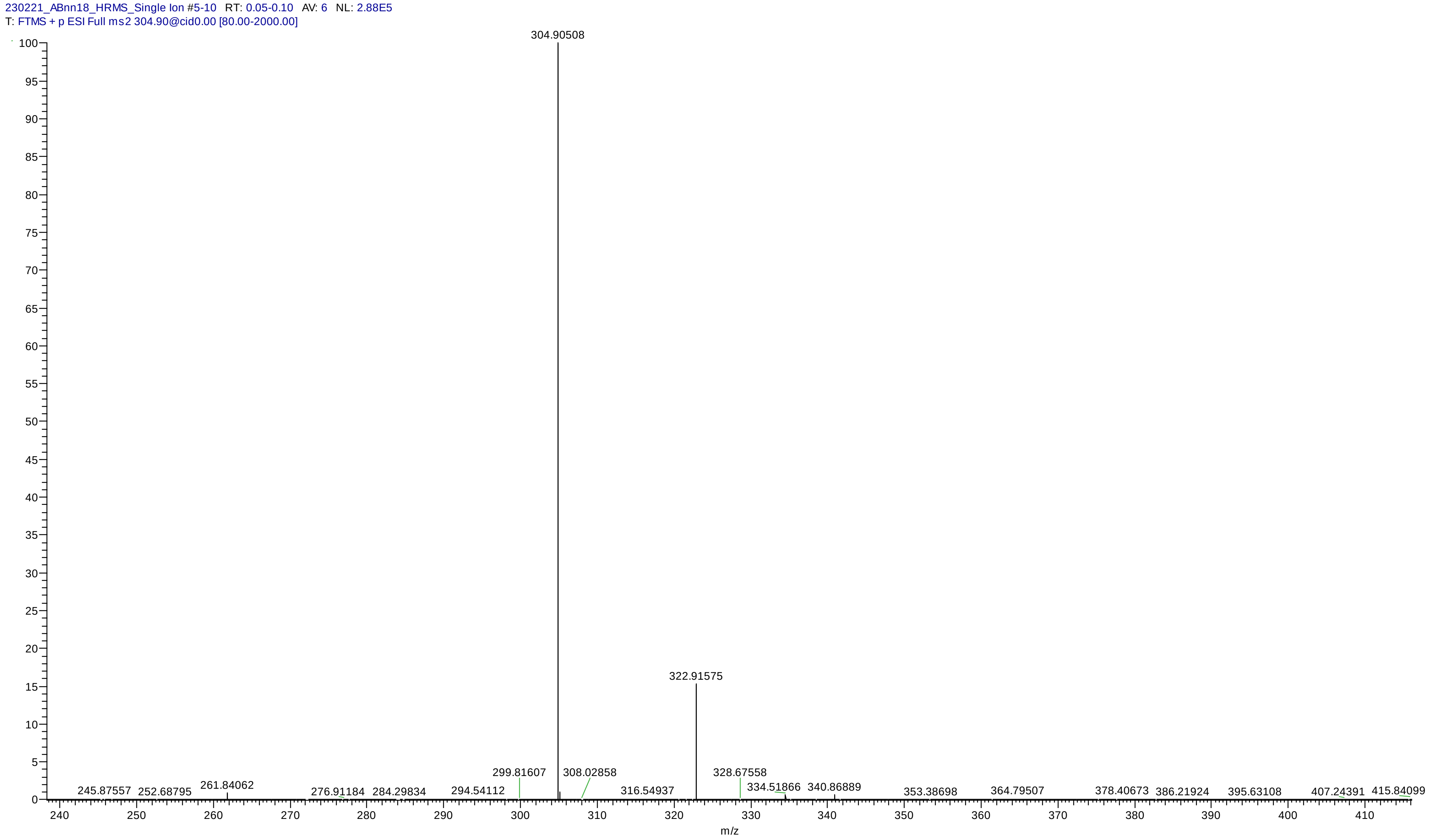
230221_ABnn18_HRMS_Single Ion #5-10 RT: 0.05-0.10 AV: 6 NL: 2.88E5
T: FTMS + p ESI Full ms2 304.90@cid0.00 [80.00-2000.00]
304.90508
322.91575
299.81607
308.02858
328.67558
245.87557
252.68795
261.84062
276.91184
284.29834
294.54112
316.54937
334.51866
340.86889
353.38698
364.79507
378.40673
386.21924
395.63108
407.24391
415.84099
m/z

ABMM-23

Compound Spectrum List Report

**Analysis Info**	ABMM-23	Acquisition Date	27/1/2021
Method	Kailani _MS/MS	Operator	Demo User
		Instrument	impact II 1825265.10265

Acquisition Parameter

Source Type	ESI	Ion Polarity	Positive	Set Nebulizer	2.0 Bar
Focus	Active	Set Capillary	2500 V	Set Dry Heater	200 °C
Scan Begin	30 m/z	Set End Plate Offset	-500 V	Set Dry Gas	8.0 l/min
Scan End	1000 m/z	Set Charging Voltage	2000 V		

C16H15FO5 + NH4

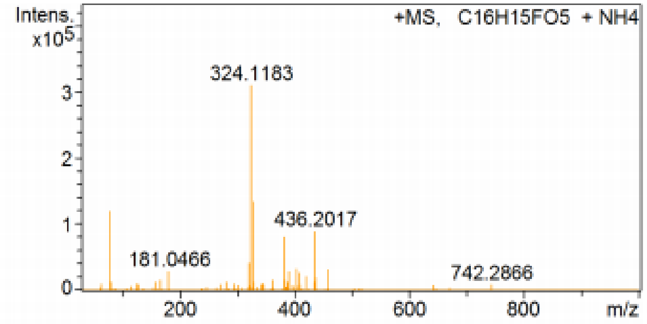

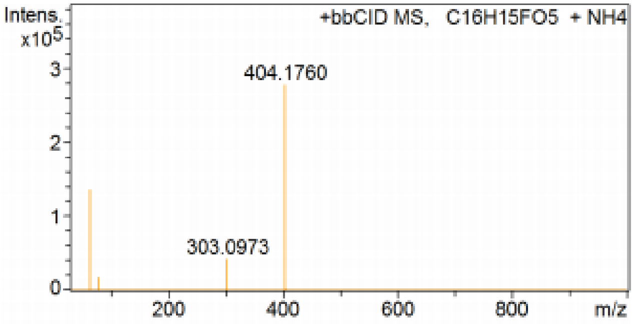

#	m/z	Res.	S/N	I	I %	FWHM
1	79.0195	21982	388.6	120771	38.9	0.0036
2	181.0466	27545	98.1	30481	9.8	0.0066
3	321.0712	33656	139.2	43279	13.9	0.0095
4	324.1183	40442	999.4	310611	100.0	0.0080
5	329.0737	36004	433.9	134865	43.4	0.0091
6	382.1595	35342	263.3	81839	26.3	0.0108
7	391.1578	34525	95.8	29782	9.6	0.0113
8	404.1760	34039	107.8	33490	10.8	0.0119
9	436.2017	35286	289.9	90112	29.0	0.0124
10	459.1481	37259	105.5	32778	10.6	0.0123

#	m/z	Res.	S/N	I	I %	FWHM
1	63.9964	19563	485.6	137131	49.3	0.0033
2	79.0196	20851	66.9	18884	6.8	0.0038
3	303.0608	32810	10.6	2981	1.1	0.0092
4	303.0973	33301	152.6	43085	15.5	0.0091
5	403.7835	31850	9.1	2574	0.9	0.0127
6	404.0919	29047	12.9	3644	1.3	0.0139
7	404.1427	30274	4.8	1363	0.5	0.0133
8	404.1760	36649	984.0	277886	100.0	0.0110
9	404.2798	28361	11.0	3115	1.1	0.0143
10	405.1789	34087	11.5	3245	1.2	0.0119

Compound Spectrum List Report

**Analysis Info**	ABMM-24	Acquisition Date	27/1/2021
Method	Kailani _MS/MS	Operator	Demo User
		Instrument	impact II 1825265.10265

Acquisition Parameter

Source Type	ESI	Ion Polarity	Positive	Set Nebulizer	2.0 Bar
Focus	Active	Set Capillary	2500 V	Set Dry Heater	200 °C
Scan Begin	30 m/z	Set End Plate Offset	-500 V	Set Dry Gas	8.0 l/min
Scan End	1000 m/z	Set Charging Voltage	2000 V		

C17H15BrO3

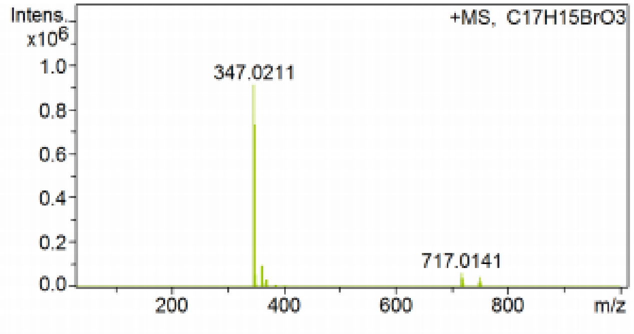

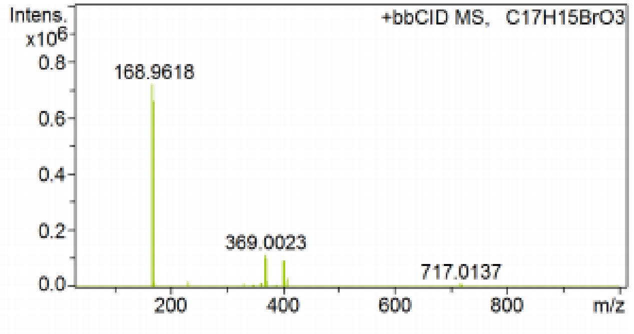

#	m/z	Res.	S/N	I	I %	FWHM
1	347.0211	24992	998.1	913088	100.0	0.0139
2	348.0243	41824	101.0	92377	10.1	0.0083
3	349.0192	25243	798.6	730584	80.0	0.0138
4	350.0223	42183	65.4	59849	6.6	0.0083
5	361.0362	35548	106.4	97370	10.7	0.0102
6	363.0343	35458	109.2	99932	10.9	0.0102
7	715.0158	41075	42.4	38786	4.2	0.0174
8	717.0141	43870	72.8	66607	7.3	0.0163
9	719.0127	38861	45.4	41538	4.5	0.0185
10	749.0393	36005	52.3	47889	5.2	0.0208

#	m/z	Res.	S/N	I	I %	FWHM
1	168.9618	21565	960.2	721344	100.0	0.0078
2	170.9597	22113	879.6	660729	91.6	0.0077
3	232.0661	30533	24.4	18326	2.5	0.0076
4	369.0023	41501	154.6	116161	16.1	0.0089
5	371.0003	41044	137.2	103039	14.3	0.0090
6	372.0036	35442	26.4	19817	2.7	0.0105
7	401.0281	35101	127.4	95701	13.3	0.0114
8	403.0261	35358	126.3	94896	13.2	0.0114
9	404.0296	33197	24.6	18506	2.6	0.0122
10	409.1670	34389	44.8	33661	4.7	0.0119

ABMM-25

Compound Spectrum List Report

**Analysis Info**	ABMM-25	Acquisition Date	27/1/2021
Method	Kailani _MS/MS	Operator	Demo User
		Instrument	impact II 1825265.10265

Acquisition Parameter

Source Type	ESI	Ion Polarity	Positive	Set Nebulizer	2.0 Bar
Focus	Active	Set Capillary	2500 V	Set Dry Heater	200 °C
Scan Begin	30 m/z	Set End Plate Offset	-500 V	Set Dry Gas	8.0 l/min
Scan End	1000 m/z	Set Charging Voltage	2000 V		

C17H17BrO3

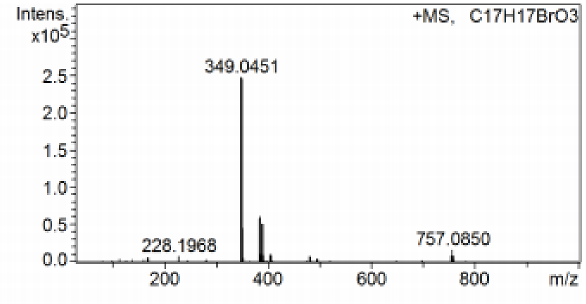

#	m/z	Res.	S/N	I	I %	FWHM
1	349.0451	38705	21902096.0	247809	100.0	0.0090
2	350.0485	37849	4358285.5	49311	19.9	0.0092
3	351.0432	38805	21757168.0	246169	99.3	0.0090
4	352.0466	37288	4106186.8	46459	18.7	0.0094
5	384.0827	38233	5235239.5	59234	23.9	0.0100
6	385.0860	37001	1070110.5	12108	4.9	0.0104
7	386.0808	38405	5448012.5	61641	24.9	0.0101
8	389.0381	38208	4599869.5	52045	21.0	0.0102
9	391.0362	37777	4525279.0	51201	20.7	0.0104
10	757.0850	38763	1542967.6	17458	7.0	0.0195

ABMM-26

Compound Spectrum List Report

**Analysis Info**	ABMM-26	Acquisition Date	27/1/2021
Method	Kailani _MS/MS	Operator	Demo User
		Instrument	impact II 1825265.10265

Acquisition Parameter

Source Type	ESI	Ion Polarity	Positive	Set Nebulizer	2.0 Bar
Focus	Active	Set Capillary	2500 V	Set Dry Heater	200 °C
Scan Begin	30 m/z	Set End Plate Offset	-500 V	Set Dry Gas	8.0 l/min
Scan End	1000 m/z	Set Charging Voltage	2000 V		

C16H13BrO3

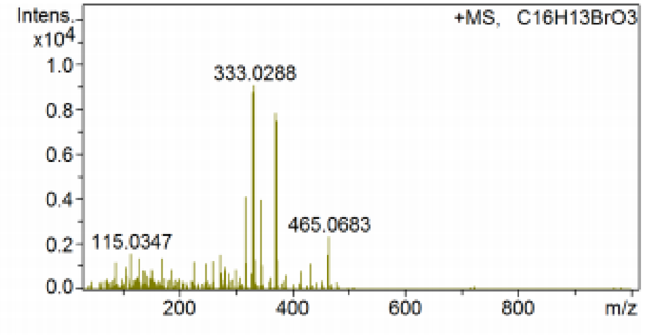

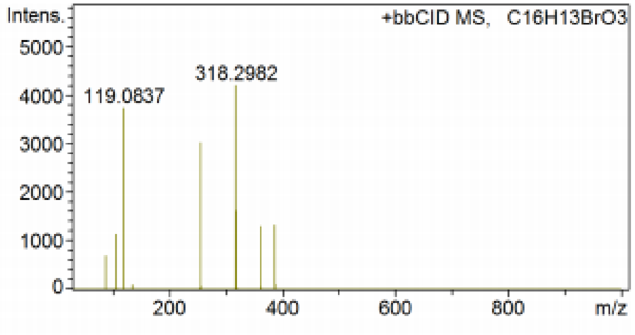

#	m/z	Res.	S/N	I	I %	FWHM
1	115.0347	24082	176.1	1601	17.6	0.0048
2	318.2982	35726	453.1	4119	45.3	0.0089
3	331.0307	35706	960.8	8734	96.2	0.0093
4	332.0338	36576	179.1	1628	17.9	0.0091
5	333.0288	35441	999.3	9084	100.0	0.0094
6	346.2204	36933	438.0	3982	43.8	0.0094
7	371.0233	36819	860.2	7820	86.1	0.0101
8	373.0214	36387	822.9	7480	82.4	0.0103
9	463.0705	38102	171.9	1563	17.2	0.0122
10	465.0683	35904	258.9	2353	25.9	0.0130

#	m/z	Res.	S/N	I	I %	FWHM
1	88.0740	21720	166.9	704	16.7	0.0041
2	106.0633	22872	93.5	394	9.4	0.0046
3	106.0845	23160	271.9	1146	27.2	0.0046
4	119.0837	24378	885.5	3732	88.6	0.0049
5	256.2615	33783	718.8	3029	72.0	0.0076
6	318.2982	36337	999.0	4210	100.0	0.0088
7	318.8433	33472	73.1	308	7.3	0.0095
8	319.3016	35646	389.0	1639	38.9	0.0090
9	362.3245	37356	309.8	1306	31.0	0.0097
10	386.9972	36028	316.7	1335	31.7	0.0107

ABMM-27

Compound Spectrum List Report

**Analysis Info**	ABMM-27	Acquisition Date	27/1/2021
Method	Kailani _MS/MS	Operator	Demo User
		Instrument	impact II 1825265.10265

Acquisition Parameter

Source Type	ESI	Ion Polarity	Positive	Set Nebulizer	2.0 Bar
Focus	Active	Set Capillary	2500 V	Set Dry Heater	200 °C
Scan Begin	30 m/z	Set End Plate Offset	-500 V	Set Dry Gas	8.0 l/min
Scan End	1000 m/z	Set Charging Voltage	2000 V		

C16H11FO4

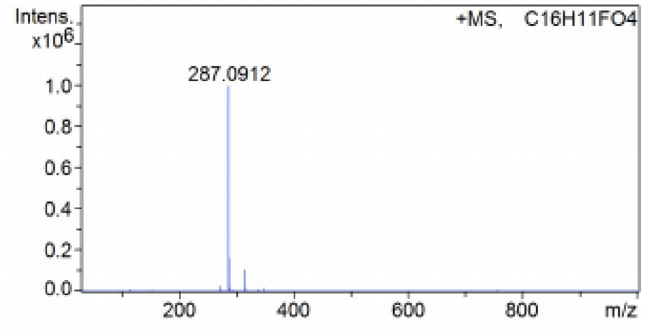

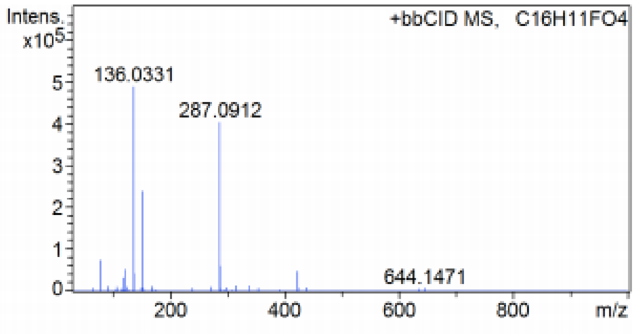

#	m/z	Res.	S/N	I	I %	FWHM
1	115.0312	22562	9.9	9946	1.0	0.0051
2	273.0761	34239	25.9	26080	2.6	0.0080
3	287.0912	37340	990.7	995811	100.0	0.0077
4	288.0944	33568	163.2	164044	16.5	0.0086
5	289.0969	29817	18.8	18859	1.9	0.0097
6	295.0574	31809	10.7	10791	1.1	0.0093
7	316.0856	34592	107.3	107820	10.8	0.0091
8	317.0888	31425	19.2	19342	1.9	0.0101
9	338.0670	32760	10.5	10518	1.1	0.0103
10	349.0301	36839	16.8	16875	1.7	0.0095

#	m/z	Res.	S/N	I	I %	FWHM
1	78.0423	20451	148.8	76572	15.6	0.0038
2	118.0469	23409	67.0	34501	7.0	0.0050
3	121.0227	23741	107.1	55090	11.2	0.0051
4	136.0331	25709	952.7	490247	100.0	0.0053
5	137.0381	15997	85.2	43838	8.9	0.0086
6	152.0639	26395	466.4	240010	49.0	0.0058
7	287.0912	37170	783.3	403090	82.2	0.0077
8	288.0943	32572	121.8	62660	12.8	0.0088
9	316.0856	32785	28.1	14478	3.0	0.0096
10	423.0419	35446	96.6	49688	10.1	0.0119

ABMM-28

Compound Spectrum List Report

Analysis Info

Analysis Name	\\Esitof\d\Data\March_29_2021\March_30_2021_Dr Ali ABMM-28_62_1_1895.d	Acquisition Date	3/30/2021 4:00:59 PM
Method	TargetScreener_impact-II_POS_bbCID.m	Operator	Demo User
Sample Name	March_30_2021_Dr Ali ABMM-28	Instrument	impact II 1825265.10265
Comment			

Acquisition Parameter

Source Type	ESI	Ion Polarity	Positive	Set Nebulizer	2.0 Bar
Focus	Active	Set Capillary	2500 V	Set Dry Heater	200 °C
Scan Begin	30 m/z	Set End Plate Offset	-500 V	Set Dry Gas	8.0 l/min
Scan End	1000 m/z	Set Collision Cell RF	1000.0 Vpp	Set Divert Valve	Waste

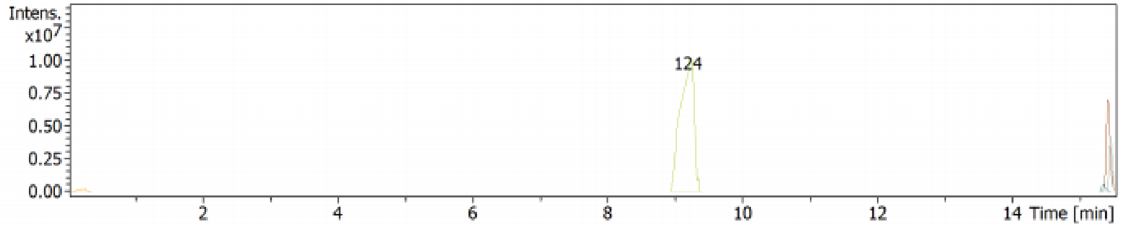

#	RT [min]	Area	Int. Type	I	S/N	Trace	Max. m/z	FWHM [min]
124	9.2	142352960	Dissect	8962920	21358.4	Dissect Cmpd 124, Dissect, 9.2 min	305.0584	0.2

C16H13ClO4

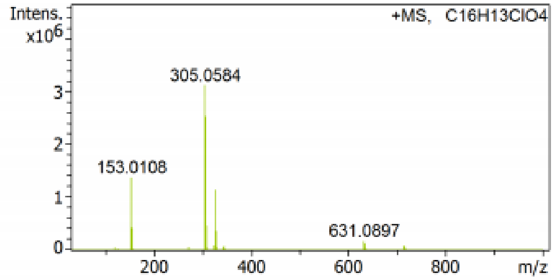

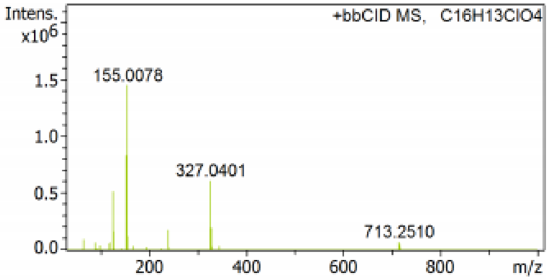

#	m/z	Res.	S/N	I	I %	FWHM
1	153.0108	28475	413.5	1371625	44.1	0.0054
2	155.0078	28277	130.4	432573	13.9	0.0055
3	305.0584	21424	938.6	3113619	100.0	0.0142
4	306.0615	39244	425.2	1410371	45.3	0.0078
5	307.0552	39423	761.6	2526172	81.1	0.0078
6	308.0587	36985	141.1	467906	15.0	0.0083
7	327.0400	40052	345.6	1146275	36.8	0.0082
8	328.0434	35134	62.6	207607	6.7	0.0093
9	329.0372	36048	113.0	374828	12.0	0.0091
10	631.0897	38113	55.7	184764	5.9	0.0166

#	m/z	Res.	S/N	I	I %	FWHM
1	125.0157	27027	349.8	518385	35.9	0.0046
2	127.0128	26680	115.0	170368	11.8	0.0048
3	153.0113	16435	560.8	831122	57.5	0.0093
4	154.0141	29195	274.7	407171	28.2	0.0053
5	155.0078	28295	975.6	1445866	100.0	0.0055
6	156.0112	28426	86.4	127997	8.9	0.0055
7	240.1951	34020	122.1	181013	12.5	0.0071
8	327.0401	38414	411.1	609264	42.1	0.0085
9	328.0436	33985	72.5	107474	7.4	0.0097
10	329.0374	34834	135.6	200920	13.9	0.0094

ABMM-32

Compound Spectrum List Report

**Analysis Info**	ABMM-28	Acquisition Date	27/1/2021
Method	Kailani _MS/MS	Operator	Demo User
		Instrument	impact II 1825265.10265

Acquisition Parameter

Source Type	ESI	Ion Polarity	Positive	Set Nebulizer	2.0 Bar
Focus	Active	Set Capillary	2500 V	Set Dry Heater	200 °C
Scan Begin	30 m/z	Set End Plate Offset	-500 V	Set Dry Gas	8.0 l/min
Scan End	1000 m/z	Set Charging Voltage	2000 V		

C15H11ClO3

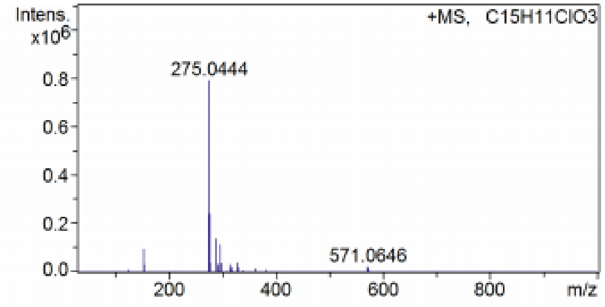

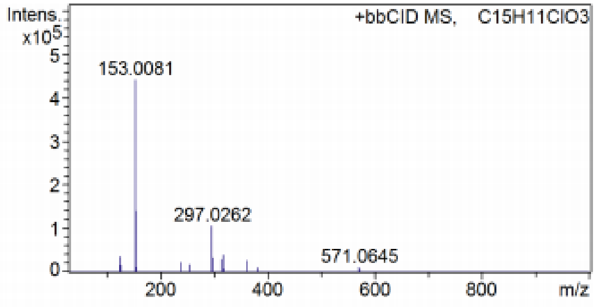

#	m/z	Res.	S/N	I	I %	FWHM
1	153.0081	27459	122.8	97882	12.4	0.0056
2	275.0444	37372	993.8	792264	100.0	0.0074
3	275.2748	34696	80.5	64142	8.1	0.0079
4	276.0478	35245	162.5	129534	16.3	0.0078
5	277.0416	35267	303.6	242052	30.6	0.0079
6	278.0450	34506	52.6	41928	5.3	0.0081
7	289.0600	38637	176.9	141049	17.8	0.0075
8	297.0262	35780	145.3	115871	14.6	0.0083
9	299.0233	34719	47.6	37987	4.8	0.0086
10	329.0524	37741	53.3	42477	5.4	0.0087

#	m/z	Res.	S/N	I	I %	FWHM
1	125.0132	26172	83.5	37227	8.4	0.0048
2	153.0081	29916	990.0	441460	100.0	0.0051
3	154.0115	27815	82.5	36799	8.3	0.0055
4	155.0052	28604	316.5	141122	32.0	0.0054
5	239.0681	32894	55.2	24602	5.6	0.0073
6	297.0262	36831	239.1	106623	24.2	0.0081
7	299.0233	34902	78.7	35081	7.9	0.0086
8	315.0368	36640	68.4	30519	6.9	0.0086
9	318.2975	37626	94.0	41939	9.5	0.0085
10	362.3237	37167	63.4	28284	6.4	0.0097

ABMM-33

Compound Spectrum List Report

Analysis Info

Analysis Name	\\Esitof\d\Data\March_29_2021\March_30_2021_Dr Ali ABMM-33_106_1_1896.d	Acquisition Date	3/30/2021 4:21:42 PM
Method	TargetScreener_impact-II_POS_bbCID.m	Operator	Demo User
Sample Name	March_30_2021_Dr Ali ABMM-33	Instrument	impact II 1825265.10265
Comment			

Acquisition Parameter

Source Type	ESI	Ion Polarity	Positive	Set Nebulizer	2.0 Bar
Focus	Active	Set Capillary	2500 V	Set Dry Heater	200 °C
Scan Begin	30 m/z	Set End Plate Offset	-500 V	Set Dry Gas	8.0 l/min
Scan End	1000 m/z	Set Collision Cell RF	1000.0 Vpp	Set Divert Valve	Waste

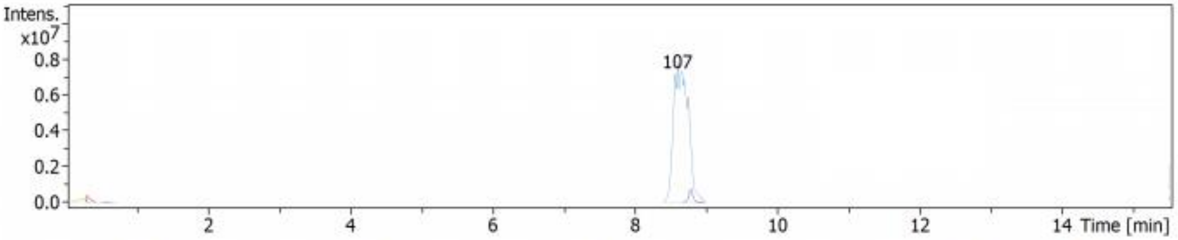

#	RT [min]	Area	Int. Type	I	S/N	Trace	Max. m/z	FWHM [min]
107	8.6	112631296	Dissect	7253972	13451.5	Dissect Cmpd 107, Dissect, 8.6 min	328.0822	0.1

C17H13NO6

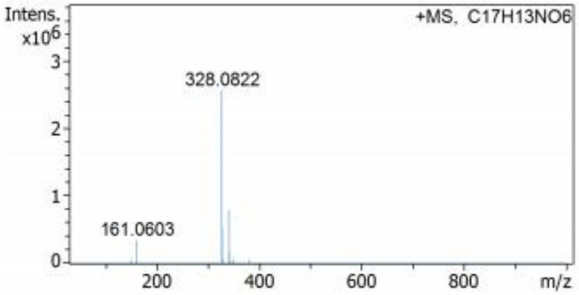

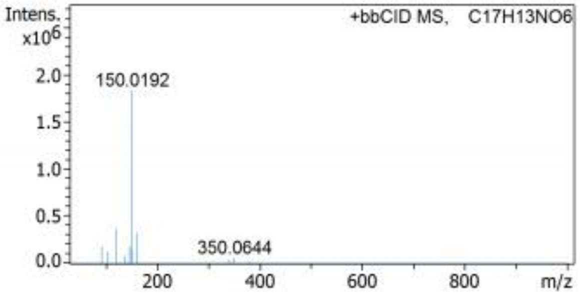

#	m/z	Res.	S/N	I	I %	FWHM
1	150.0192	27092	22.3	58127	2.3	0.0055
2	161.0603	29440	138.3	360101	14.0	0.0055
3	162.0636	27766	15.4	40183	1.6	0.0058
4	328.0822	38290	986.2	2567385	100.0	0.0086
5	329.0856	38471	211.6	550768	21.5	0.0086
6	330.0880	32752	30.9	80427	3.1	0.0101
7	342.0978	40663	306.9	799040	31.1	0.0084
8	343.1013	37021	68.7	178862	7.0	0.0093
9	350.0642	34809	25.8	67109	2.6	0.0101

#	m/z	Res.	S/N	I	I %	FWHM
1	92.0262	21885	102.6	188212	10.3	0.0042
2	104.0262	23554	71.3	130893	7.1	0.0044
3	120.0212	25231	205.7	377507	20.6	0.0048
4	136.0162	25788	50.8	93156	5.1	0.0053
5	146.0368	27704	103.4	189665	10.4	0.0053
6	150.0192	28162	997.8	1831003	100.0	0.0053
7	151.0225	27446	81.2	149072	8.1	0.0055
8	161.0603	29731	177.9	326464	17.8	0.0054
9	342.0980	35008	21.4	39235	2.1	0.0098

ABMM-34

Compound Spectrum List Report

Analysis Info

		Acquisition Date	3/30/2021 4:42:26 PM
Analysis Name	\\Esitof\d\Data\March_29_2021\March_30_2021_Dr Ali ABMM-34_107_1_1897.d		
Method	TargetScreener_impact-II_POS_bbCID.m	Operator	Demo User
Sample Name	March_30_2021_Dr Ali ABMM-34	Instrument	impact II 1825265.10265
Comment			

Acquisition Parameter

Source Type	ESI	Ion Polarity	Positive	Set Nebulizer	2.0 Bar
Focus	Active	Set Capillary	2500 V	Set Dry Heater	200 °C
Scan Begin	30 m/z	Set End Plate Offset	-500 V	Set Dry Gas	8.0 l/min
Scan End	1000 m/z	Set Collision Cell RF	1000.0 Vpp	Set Divert Valve	Waste

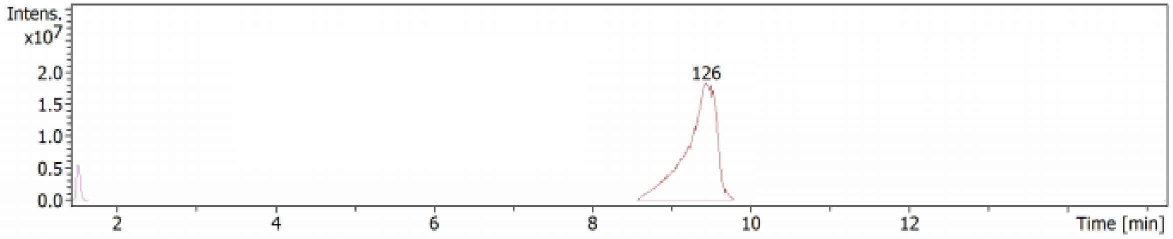

#	RT [min]	Area	Int. Type	I	S/N	Trace	Max. m/z	FWHM [min]
126	9.4	482663584	Dissect	18202466	28866.0	Dissect Cmpd 126, Dissect, 9.4 min	138.9952	0.2

Cmpd 126, Dissect, 9.4 min

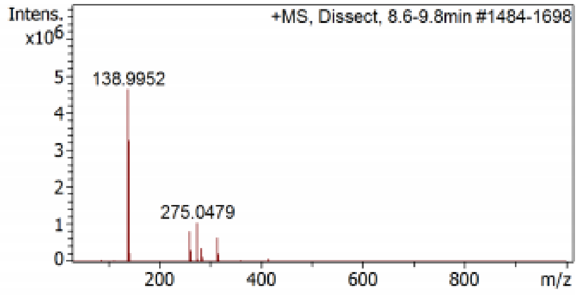

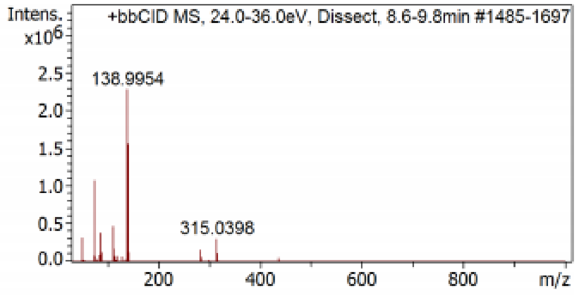

#	m/z	Res.	S/N	I	I %	FWHM
1	138.9952	19840	999.7	4630382	100.0	0.0070
2	139.9984	27036	175.2	811476	17.5	0.0052
3	140.9921	28499	705.1	3265663	70.5	0.0049
4	141.9955	26172	53.6	248322	5.4	0.0054
5	261.0316	37042	178.8	828052	17.9	0.0070
6	263.0288	34915	68.9	318982	6.9	0.0075
7	275.0479	19154	229.1	1060939	22.9	0.0144
8	283.0136	36721	81.0	375019	8.1	0.0077
9	315.0397	40714	142.4	659430	14.2	0.0077
10	317.0370	37913	49.8	230454	5.0	0.0084

#	m/z	Res.	S/N	I	I %	FWHM
1	51.0230	17160	140.7	322579	14.1	0.0030
2	75.0231	20571	472.6	1083444	47.5	0.0036
3	86.9998	21799	170.5	390925	17.1	0.0040
4	111.0000	25415	206.7	473777	20.8	0.0044
5	112.9971	24813	76.4	175095	7.7	0.0046
6	138.9954	17155	994.6	2280343	100.0	0.0081
7	139.9985	27274	177.4	406824	17.8	0.0051
8	140.9921	27831	679.8	1558658	68.4	0.0051
9	283.0137	35346	70.3	161200	7.1	0.0080
10	315.0398	40087	136.2	312362	13.7	0.0079

ABMM-35

Compound Spectrum List Report

Analysis Info

		Acquisition Date	3/30/2021 5:03:09 PM
Analysis Name	\\Esitof\d\Data\March_29_2021\March_30_2021_Dr Ali ABMM-35_108_1_1898.d		
Method	TargetScreener_impact-II_POS_bbCID.m	Operator	Demo User
Sample Name	March_30_2021_Dr Ali ABMM-35	Instrument	impact II 1825265.10265
Comment			

Acquisition Parameter

Source Type	ESI	Ion Polarity	Positive	Set Nebulizer	2.0 Bar
Focus	Active	Set Capillary	2500 V	Set Dry Heater	200 °C
Scan Begin	30 m/z	Set End Plate Offset	-500 V	Set Dry Gas	8.0 l/min
Scan End	1000 m/z	Set Collision Cell RF	1000.0 Vpp	Set Divert Valve	Waste

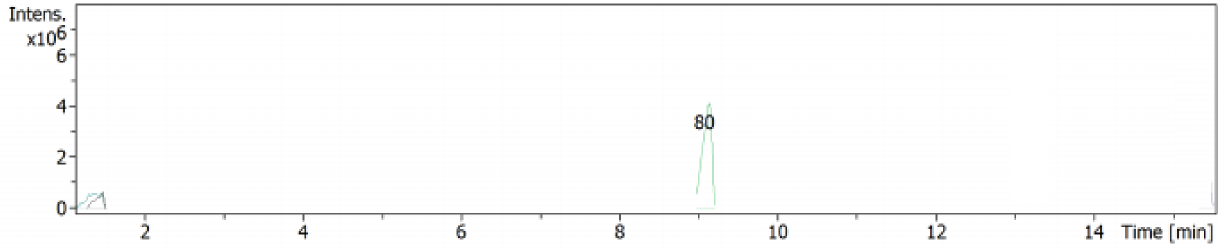

#	RT [min]	Area	Int. Type	I	S/N	Trace	Max. m/z	FWHM [min]
80	9.1	33805668	Dissect	2907746	24911.9	Dissect Cmpd 80, Dissect, 9.1 min	263.0475	0.2

C14H11ClO3

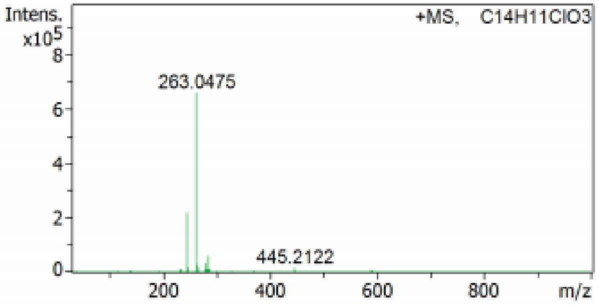

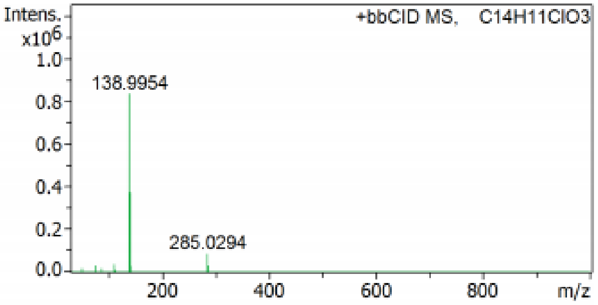

#	m/z	Res.	S/N	I	I %	FWHM
1	245.0369	29139	283.5	223122	33.8	0.0084
2	247.0338	36509	23.4	18387	2.8	0.0068
3	263.0475	25081	838.2	659640	100.0	0.0105
4	264.0509	36319	38.5	30311	4.6	0.0073
5	265.0444	37850	31.5	24777	3.8	0.0070
6	266.0478	35083	21.6	16979	2.6	0.0076
7	280.0738	38918	46.8	36818	5.6	0.0072
8	283.0743	32464	18.1	14232	2.2	0.0087
9	285.0292	38348	81.3	64016	9.7	0.0074

#	m/z	Res.	S/N	I	I %	FWHM
1	51.0230	17035	22.1	19043	2.3	0.0030
2	75.0231	20064	38.8	33483	4.0	0.0037
3	86.9999	21178	24.9	21454	2.6	0.0041
4	111.0000	25243	45.9	39562	4.7	0.0044
5	138.9954	18140	969.9	836647	100.0	0.0077
6	139.9985	27282	104.5	90149	10.8	0.0051
7	140.9921	27600	438.2	377985	45.2	0.0051
8	141.9955	27097	35.7	30815	3.7	0.0052
9	285.0294	36676	104.9	90472	10.8	0.0078
