# Supplementary material for: Design, Synthesis, Biological Evaluation and In Silico Study of Benzyloxybenzaldehyde Derivatives as Selective ALDH1A3 Inhibitors
Source: Molecules. 2021 Sep 23;26(19):5770. doi: 10.3390/molecules26195770 (PMC8510124; doi:10.3390/molecules26195770)
Supplement: Supplementary file 1 [file molecules-26-05770-s001.zip › NMR Supplementary Data.pdf]

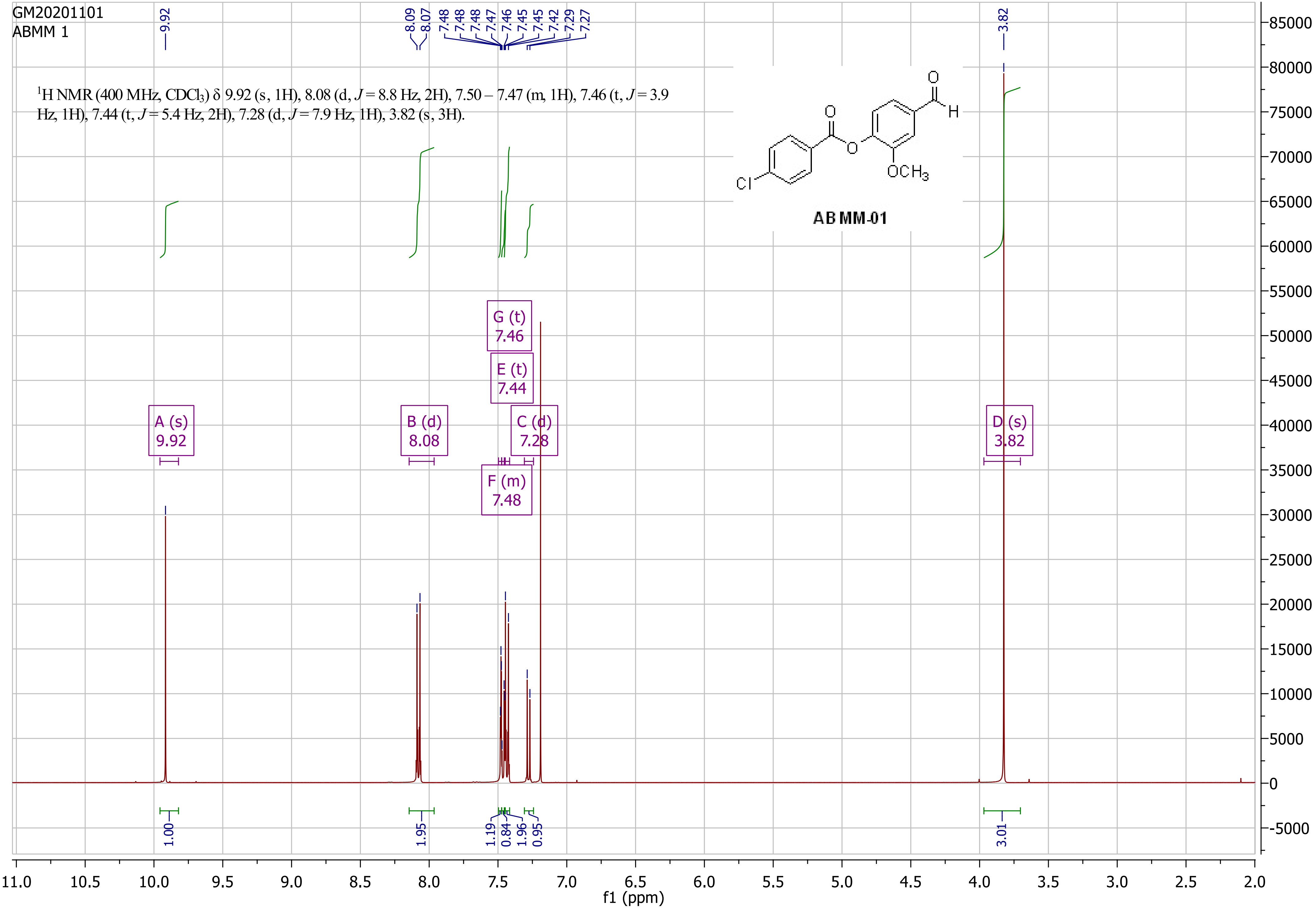
GM20201101
ABMM 1
1H NMR (400 MHz, CDCl3) δ 9.92 (s, 1H), 8.08 (d, J = 8.8 Hz, 2H), 7.50 – 7.47 (m, 1H), 7.46 (t, J = 3.9 Hz, 1H), 7.44 (t, J = 5.4 Hz, 2H), 7.28 (d, J = 7.9 Hz, 1H), 3.82 (s, 3H).
ABMM-01
A (s) 9.92
B (d) 8.08
C (d) 7.28
D (s) 3.82
E (t) 7.44
F (m) 7.48
G (t) 7.46
f1 (ppm)

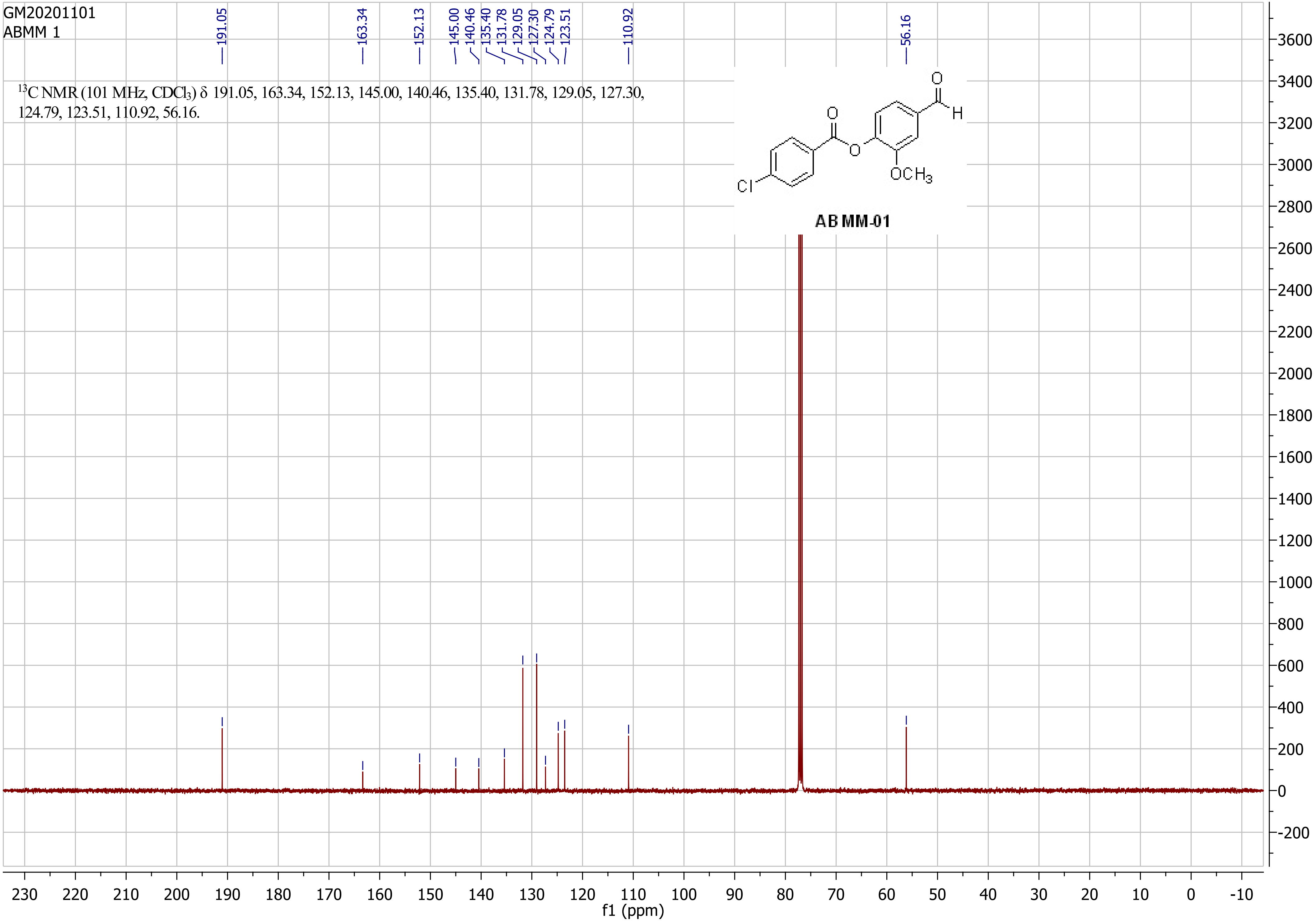

^{13}C NMR (101 MHz, $CDCl_3$) δ 191.05, 163.34, 152.13, 145.00, 140.46, 135.40, 131.78, 129.05, 127.30, 124.79, 123.51, 110.92, 56.16.

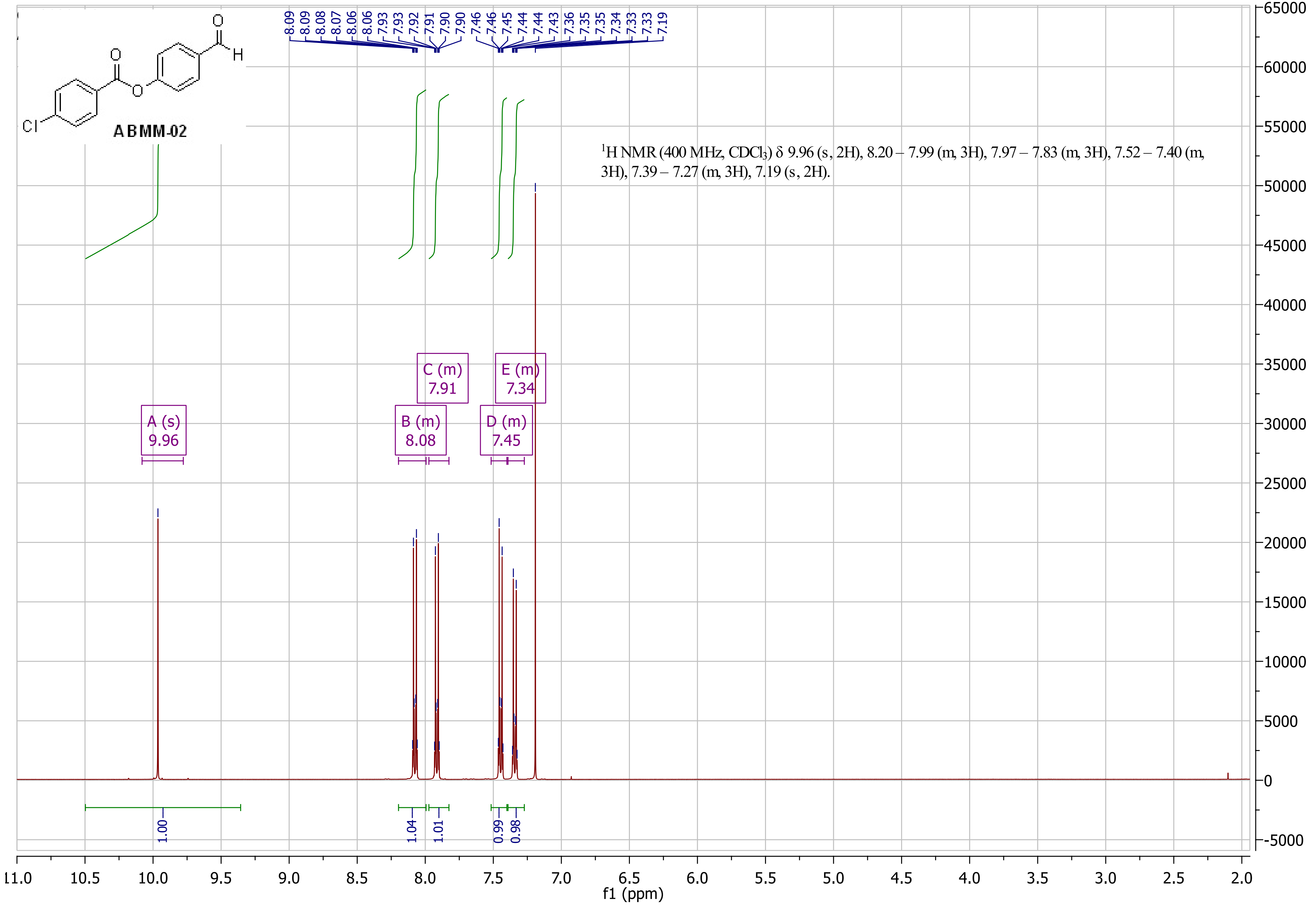

ABMM-02
Cl
O
H
8.09 8.09 8.08 8.07 8.06 7.93 7.93 7.92 7.91 7.90 7.90 7.46 7.46 7.45 7.44 7.44 7.43 7.36 7.35 7.35 7.34 7.33 7.33 7.19
^{1}H NMR (400 MHz, $CDCl_3$) δ 9.96 (s, 2H), 8.20 – 7.99 (m, 3H), 7.97 – 7.83 (m, 3H), 7.52 – 7.40 (m, 3H), 7.39 – 7.27 (m, 3H), 7.19 (s, 2H).
A (s) 9.96
B (m) 8.08
C (m) 7.91
D (m) 7.45
E (m) 7.34
1.00
1.04
1.01
0.99
0.98
f1 (ppm)

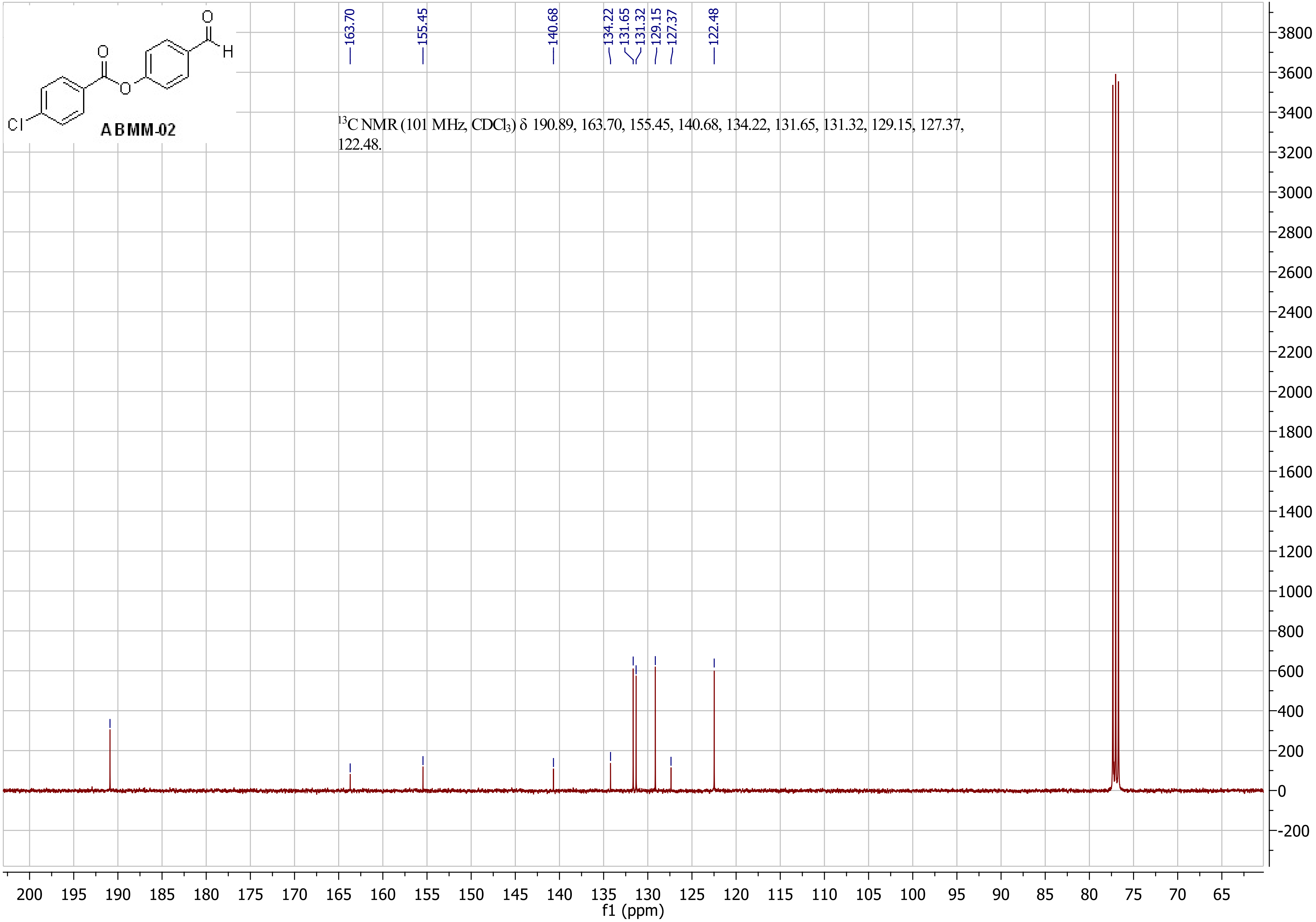

^{13}C NMR (101 MHz, $CDCl_3$) δ 190.89, 163.70, 155.45, 140.68, 134.22, 131.65, 131.32, 129.15, 127.37, 122.48.

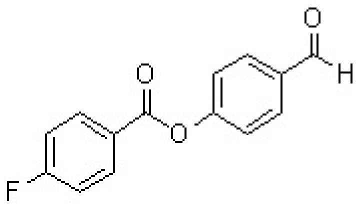

ABMM-03

^{1}H NMR (400 MHz, $CDCl_3$) δ 10.03 (s, 2H), 8.23 (dd, J = 8.9, 5.4 Hz, 4H), 7.98 (d, J = 8.6 Hz, 4H), 7.41 (d, J = 8.5 Hz, 4H), 7.21 (t, J = 8.6 Hz, 5H).

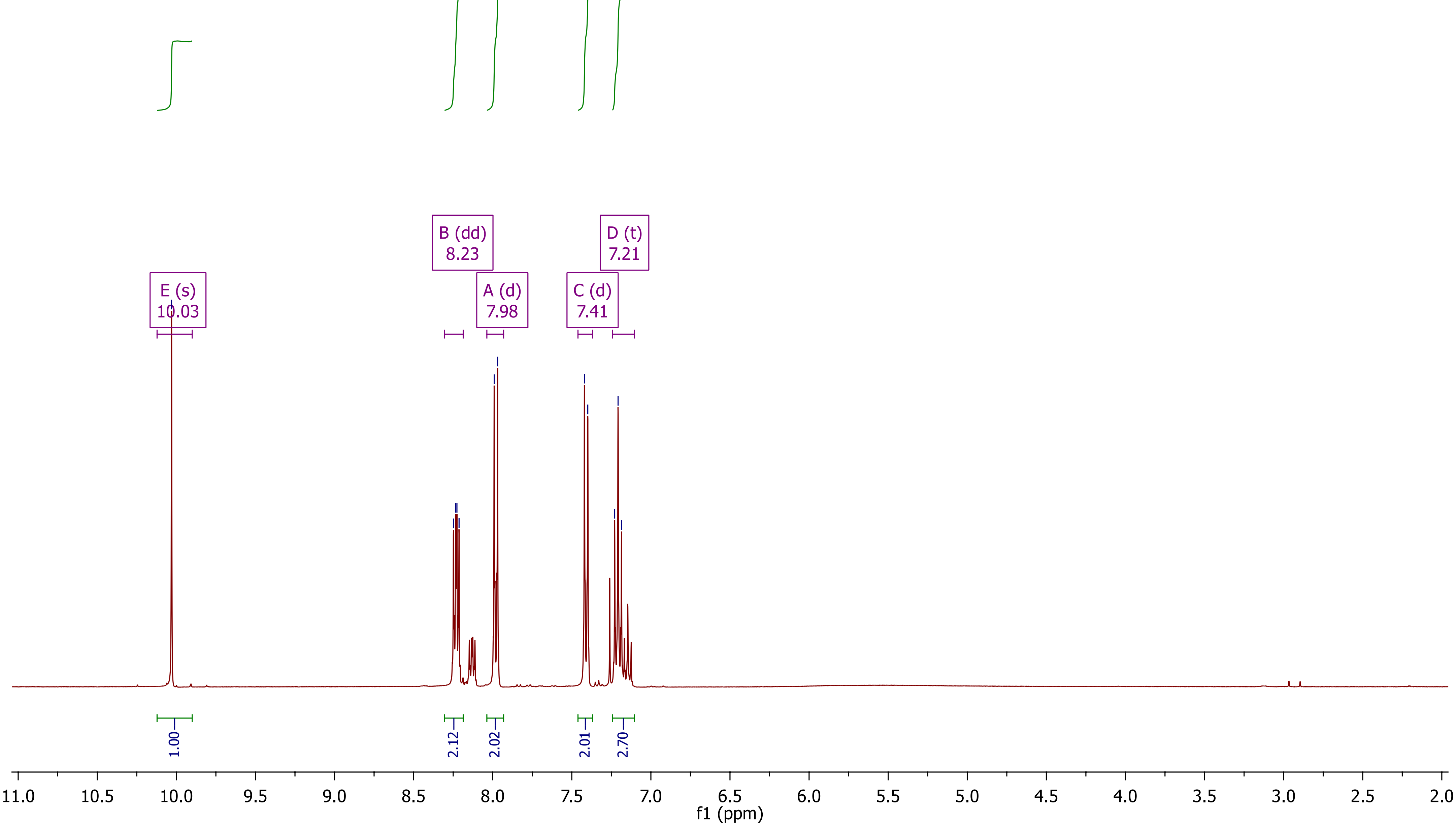

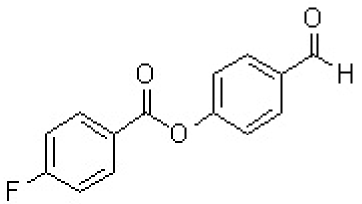

ABMM-03

^{13}C NMR (101 MHz, $CDCl_3$) δ 191.06, 170.28, 167.82, 165.28, 163.68, 155.67, 134.31, 133.15, 133.05, 131.45, 125.64, 125.33, 125.30, 122.64, 116.26, 116.04, 77.16.

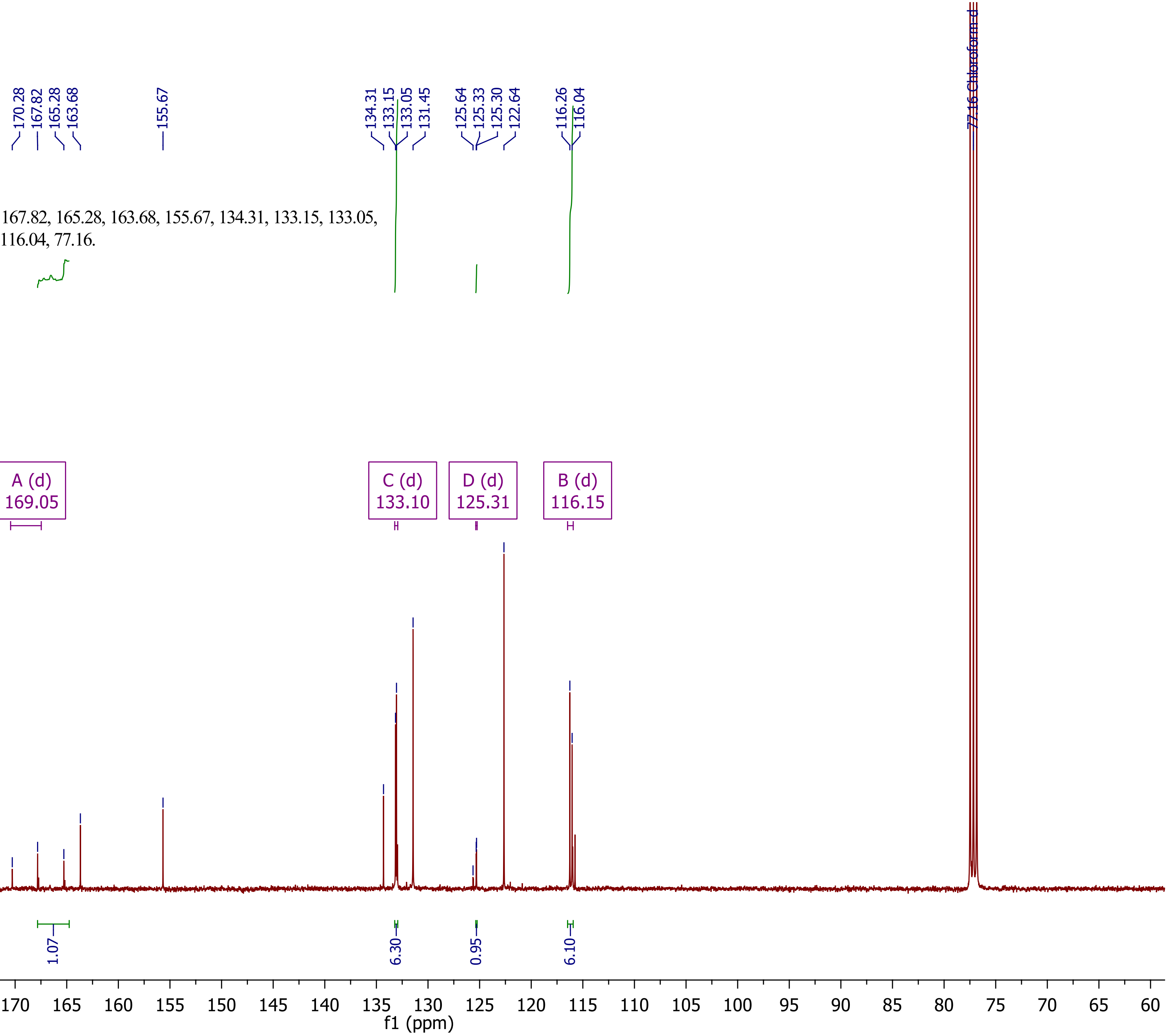

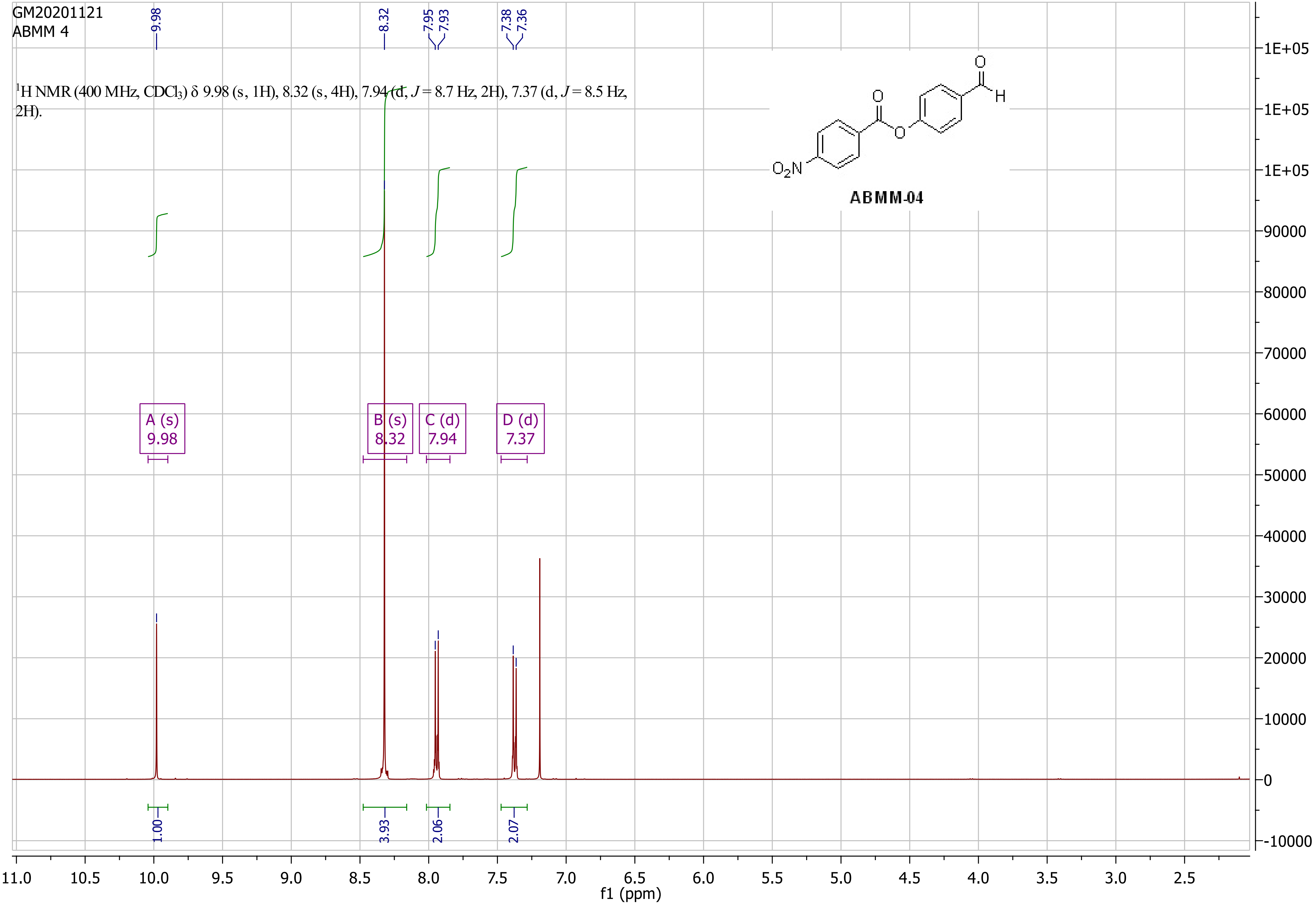
GM20201121
ABMM 4
9.98
8.32
7.95
7.93
7.38
7.36
^{1}H NMR (400 MHz, $CDCl_3$) δ 9.98 (s, 1H), 8.32 (s, 4H), 7.94 (d, J = 8.7 Hz, 2H), 7.37 (d, J = 8.5 Hz, 2H).
O
H
O
O
O_2N
ABMM-04
A (s)
9.98
B (s)
8.32
C (d)
7.94
D (d)
7.37
1.00
3.93
2.06
2.07
1E+05
1E+05
1E+05
90000
80000
70000
60000
50000
40000
30000
20000
10000
0
-10000
11.0
10.5
10.0
9.5
9.0
8.5
8.0
7.5
7.0
6.5
6.0
5.5
5.0
4.5
4.0
3.5
3.0
2.5
f1 (ppm)

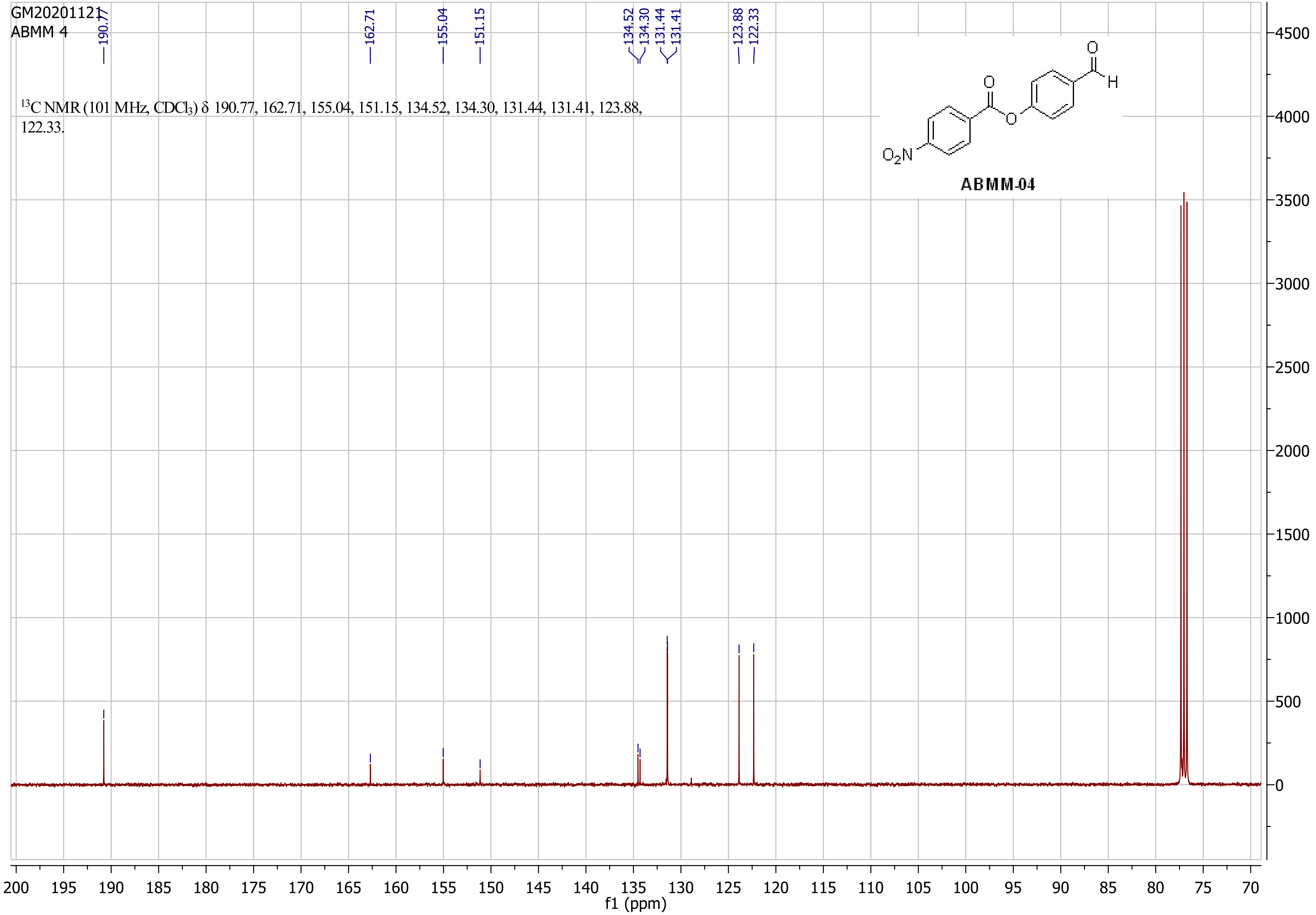

^{13}C NMR (101 MHz, $CDCl_3$) δ 190.77, 162.71, 155.04, 151.15, 134.52, 134.30, 131.44, 131.41, 123.88, 122.33.

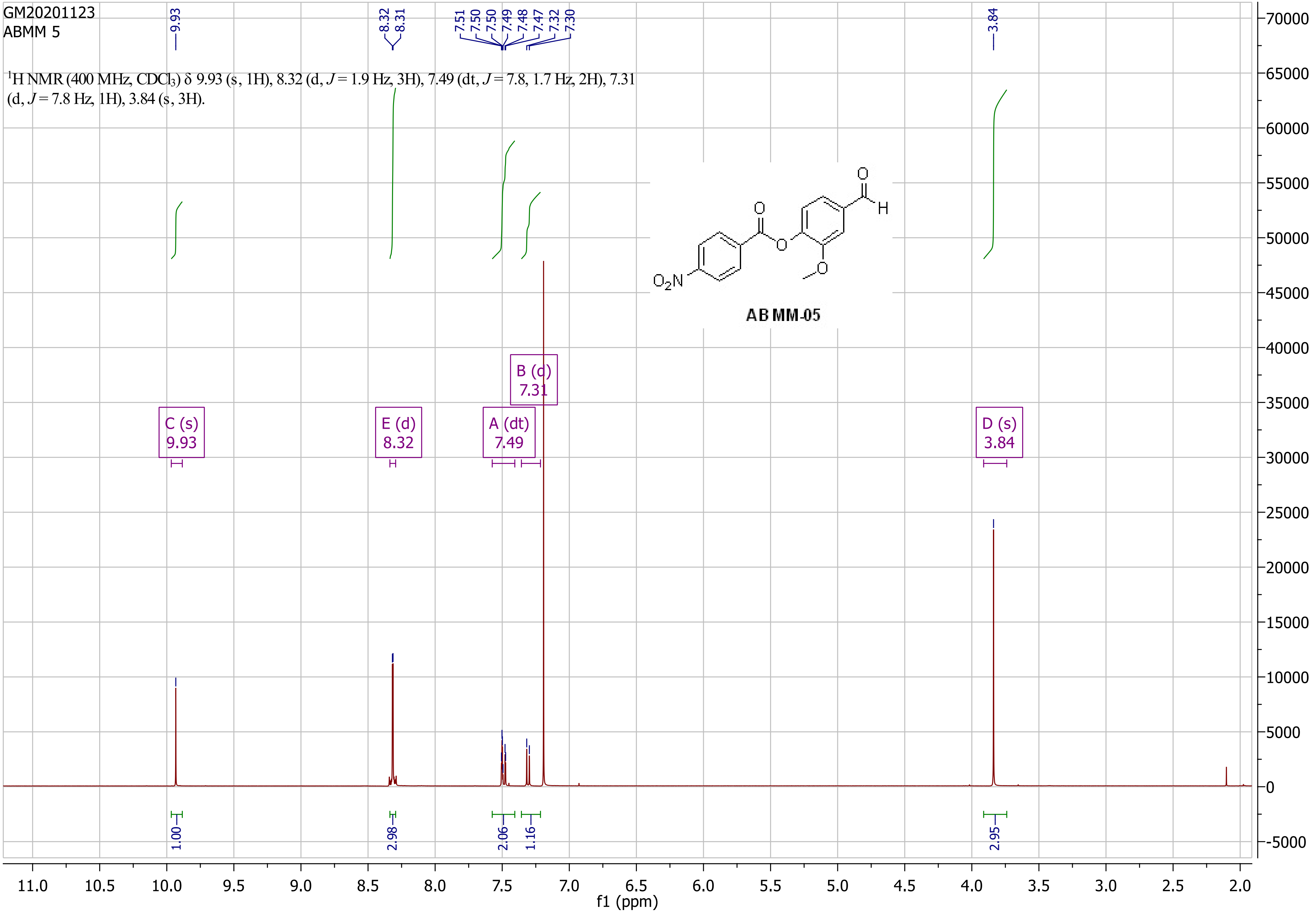

^{1}H NMR (400 MHz, $CDCl_3$) δ 9.93 (s, 1H), 8.32 (d, J = 1.9 Hz, 3H), 7.49 (dt, J = 7.8, 1.7 Hz, 2H), 7.31 (d, J = 7.8 Hz, 1H), 3.84 (s, 3H).

^{13}C NMR (101 MHz, $CDCl_3$) δ 190.94, 162.33, 151.92, 151.06, 144.56, 135.68, 134.25, 131.54, 124.75, 123.79, 123.34, 111.01, 56.19.

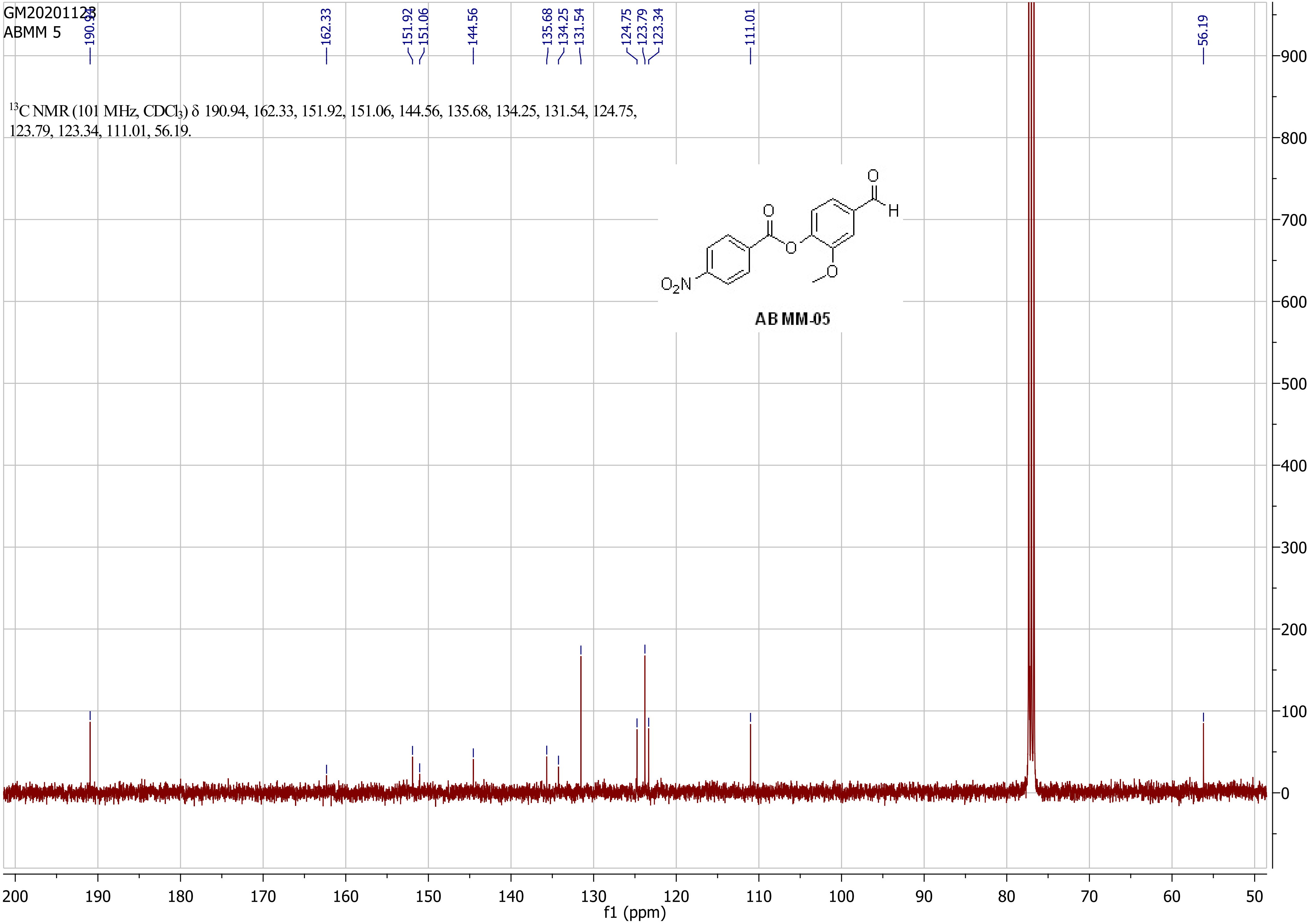

AB MM-05

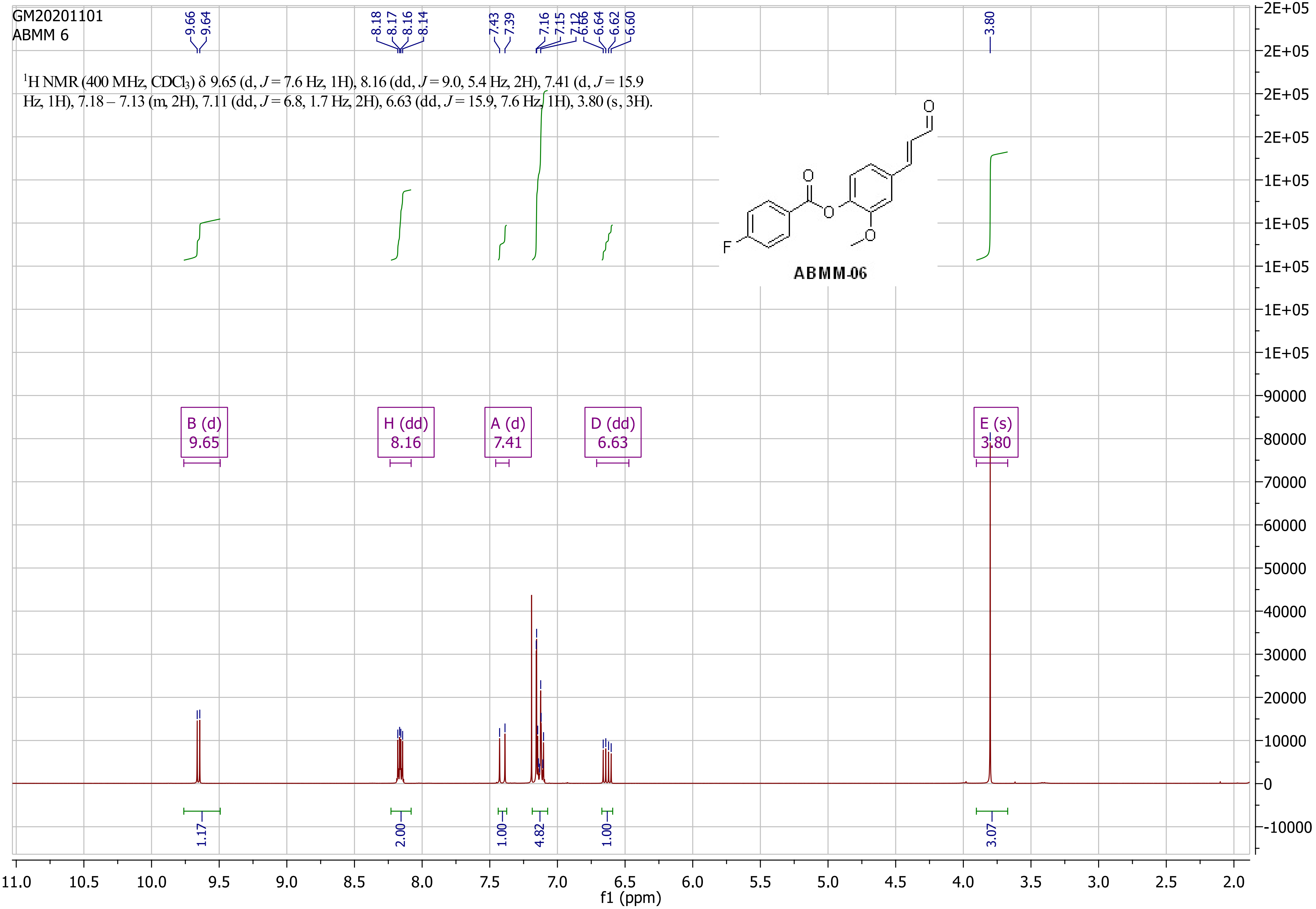

GM20201101
ABMM 6
^{1}H NMR (400 MHz, CDCl3) δ 9.65 (d, J = 7.6 Hz, 1H), 8.16 (dd, J = 9.0, 5.4 Hz, 2H), 7.41 (d, J = 15.9 Hz, 1H), 7.18 – 7.13 (m, 2H), 7.11 (dd, J = 6.8, 1.7 Hz, 2H), 6.63 (dd, J = 15.9, 7.6 Hz, 1H), 3.80 (s, 3H).
9.66
9.64
8.18
8.17
8.16
8.14
7.43
7.39
7.16
7.15
7.12
6.66
6.64
6.62
6.60
3.80
F
O
ABMM-06
B (d) 9.65
H (dd) 8.16
A (d) 7.41
D (dd) 6.63
E (s) 3.80
1.17
2.00
1.00
4.82
1.00
3.07
f1 (ppm)

GM20201101
ABMM-06

^{13}C NMR (101 MHz, $CDCl_3$) δ 193.45, 167.55, 165.01, 163.46, 151.86, 151.81, 142.35, 133.09, 133.07, 132.98, 128.83, 125.26, 125.24, 123.63, 121.93, 115.97, 115.75, 111.49, 56.03.

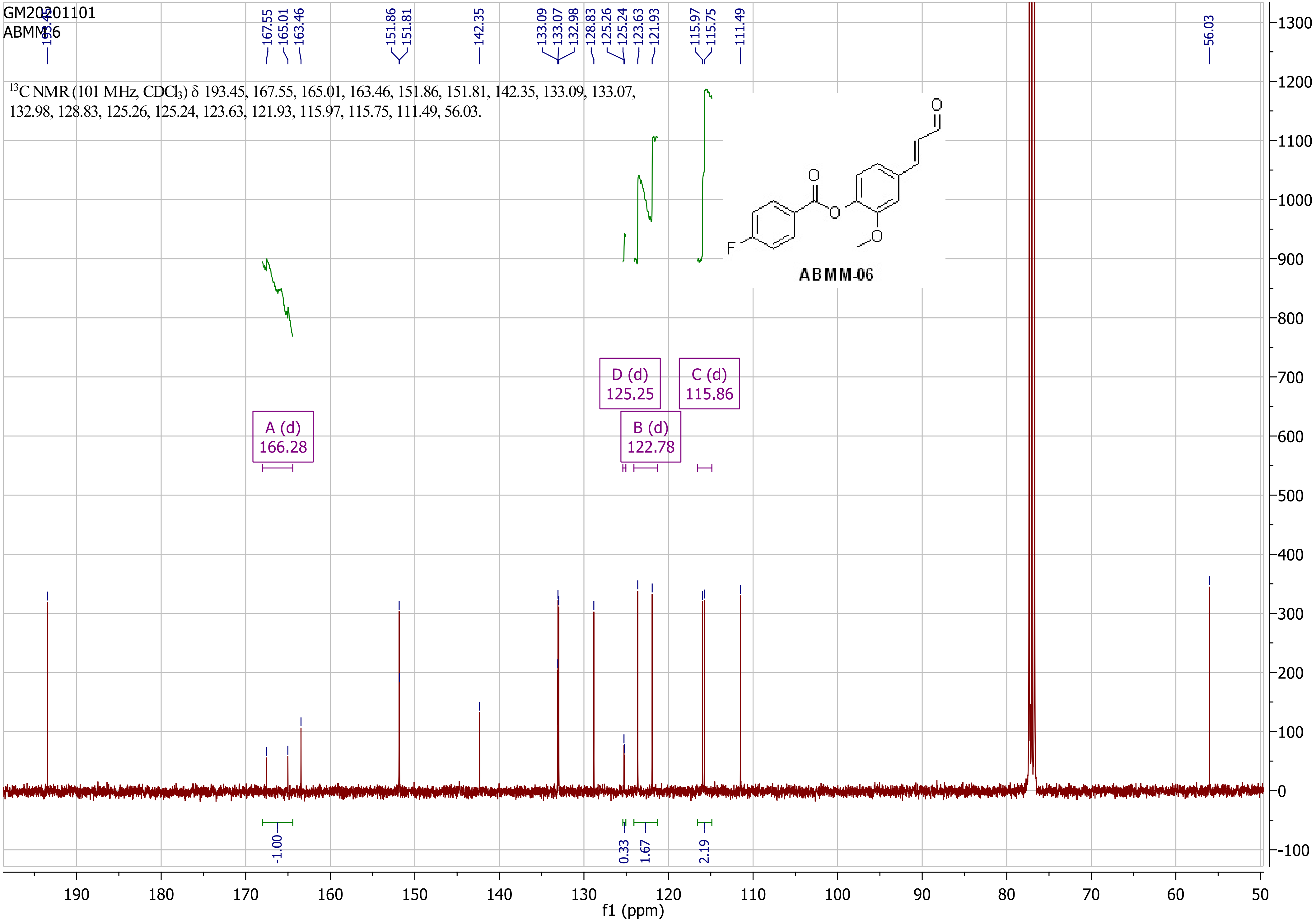

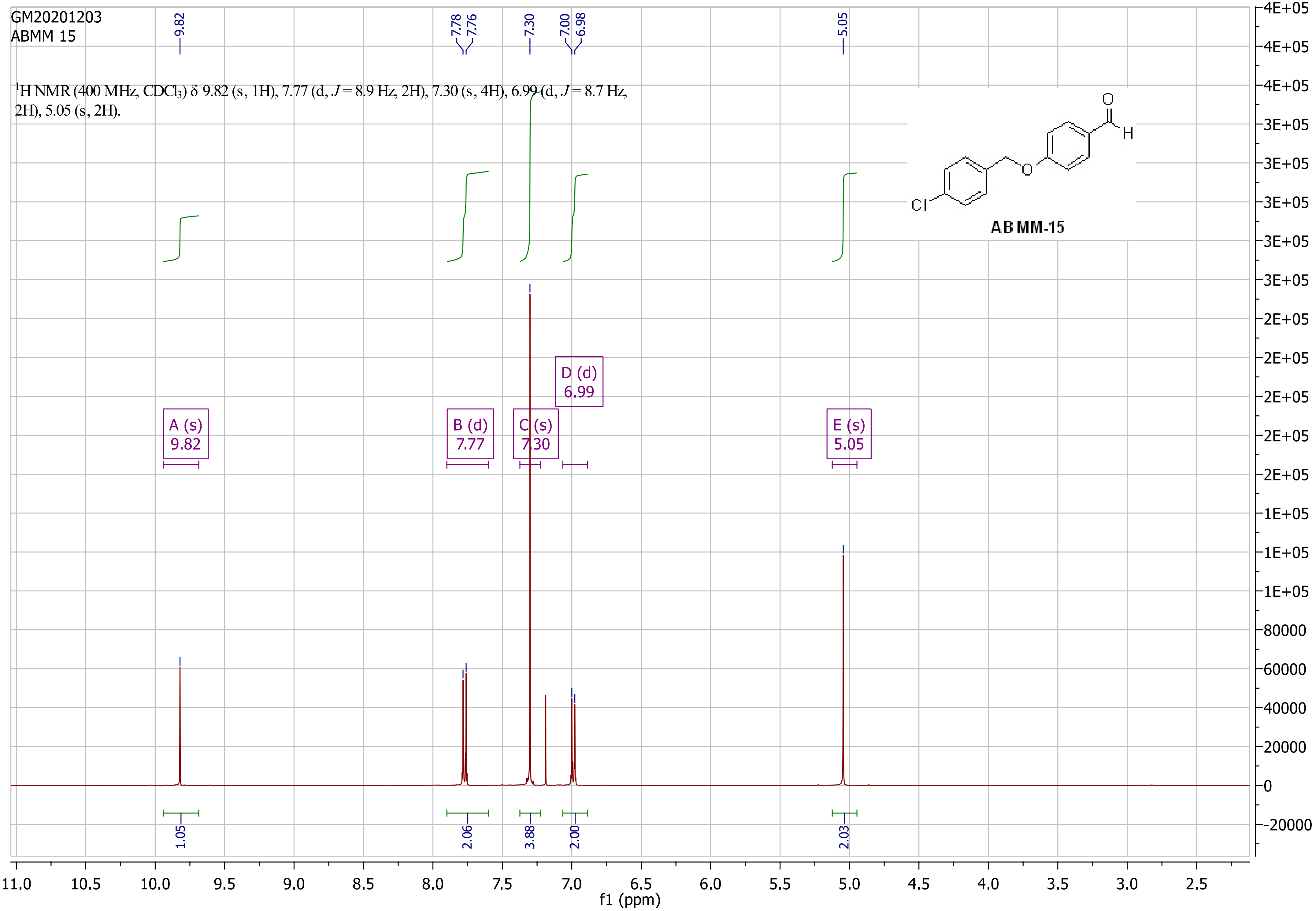
GM20201203
ABMM 15
^{1}H NMR (400 MHz, $CDCl_3$) δ 9.82 (s, 1H), 7.77 (d, J = 8.9 Hz, 2H), 7.30 (s, 4H), 6.99 (d, J = 8.7 Hz, 2H), 5.05 (s, 2H).
9.82
7.78
7.76
7.30
7.00
6.98
5.05
AB MM-15
A (s) 9.82
B (d) 7.77
C (s) 7.30
D (d) 6.99
E (s) 5.05
1.05
2.06
3.88
2.00
2.03
f1 (ppm)

GM20201203
ABMM 15

^{13}C NMR (101 MHz, $CDCl_3$) δ 190.75, 163.44, 134.46, 134.22, 132.03, 130.32, 128.95, 128.81, 115.13, 69.48.

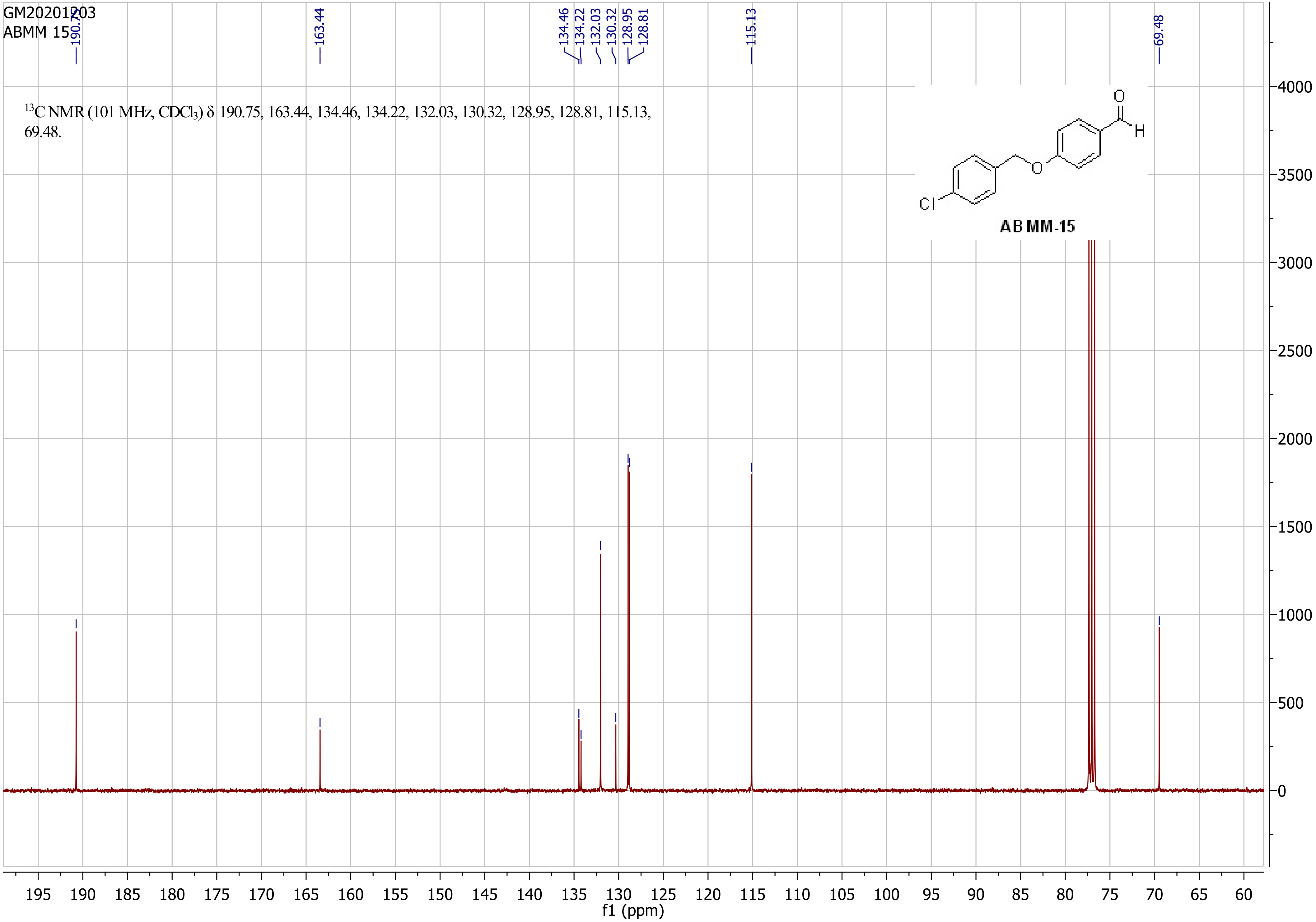

GM20201207
ABMM-16

^1H NMR (400 MHz, $CDCl_3$) δ 9.78 (s, 1H), 7.37 (d, *J* = 1.8 Hz, 1H), 7.35 – 7.26 (m, 5H), 6.89 (d, *J* = 8.2 Hz, 1H), 5.13 (s, 2H), 3.88 (s, 3H).

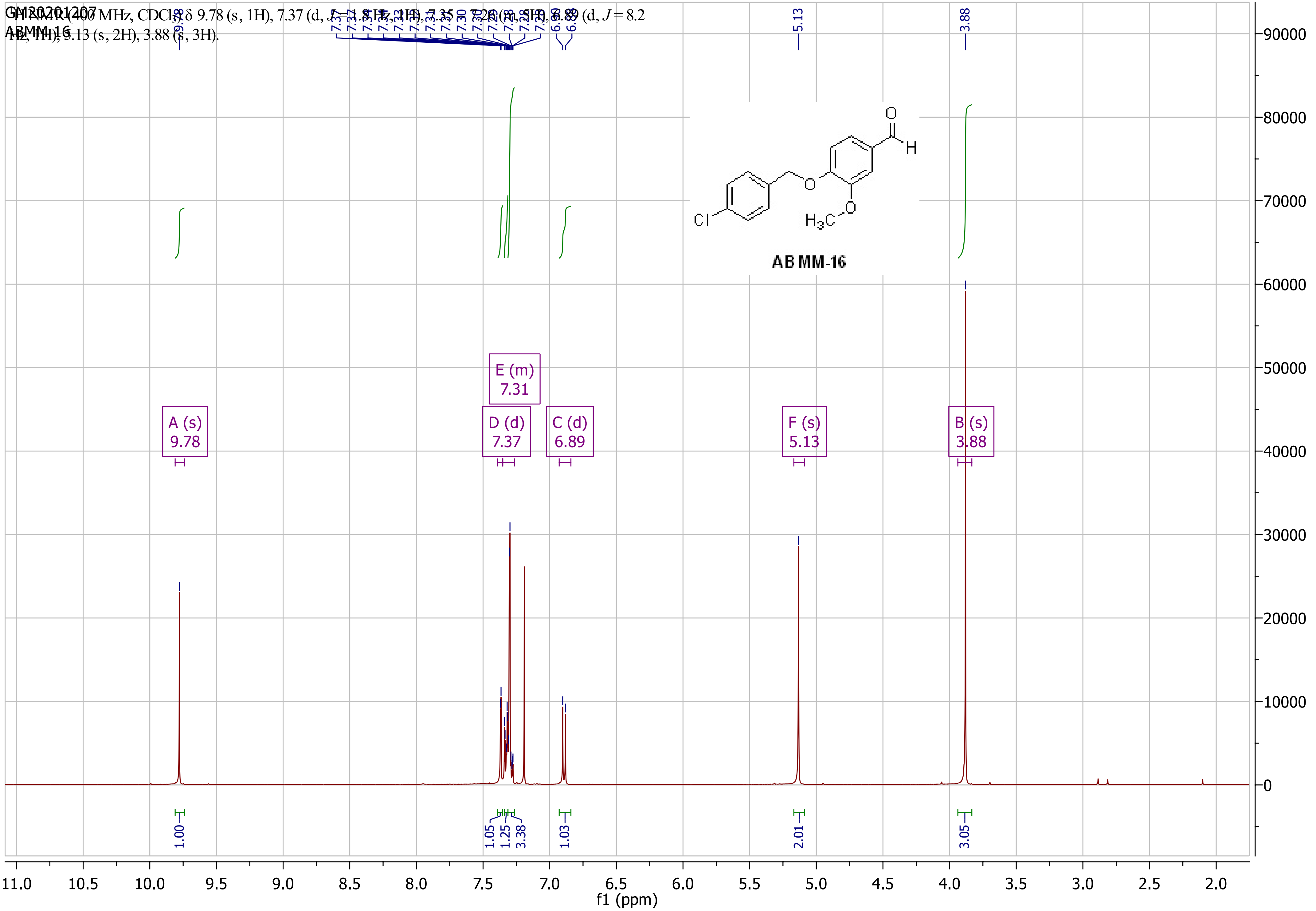

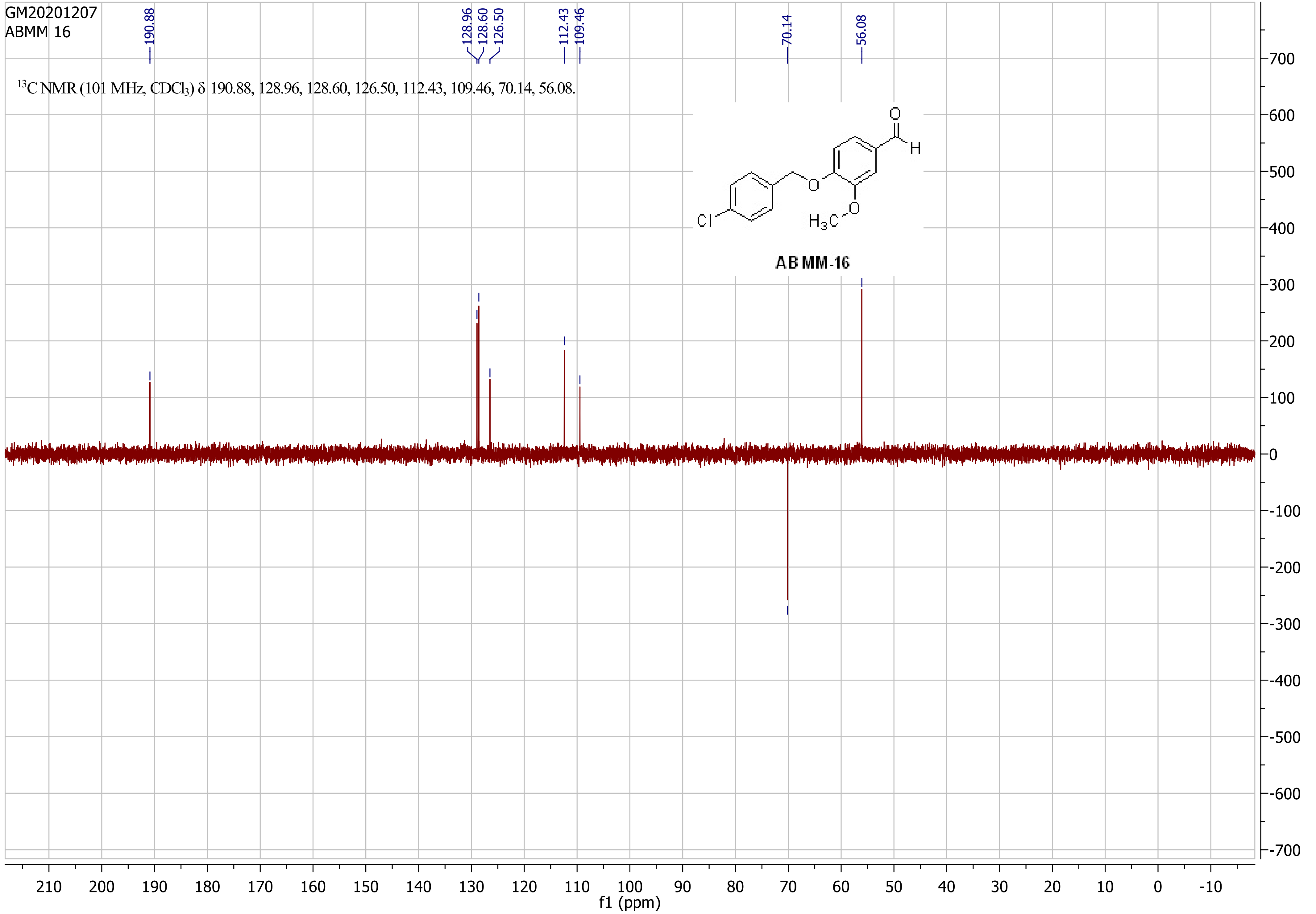
GM20201207
ABMM 16
190.88
128.96
128.60
126.50
112.43
109.46
70.14
56.08
^{13}C NMR (101 MHz, $CDCl_3$) δ 190.88, 128.96, 128.60, 126.50, 112.43, 109.46, 70.14, 56.08.
O
H
O
Cl
H_3C
O
AB MM-16
f1 (ppm)

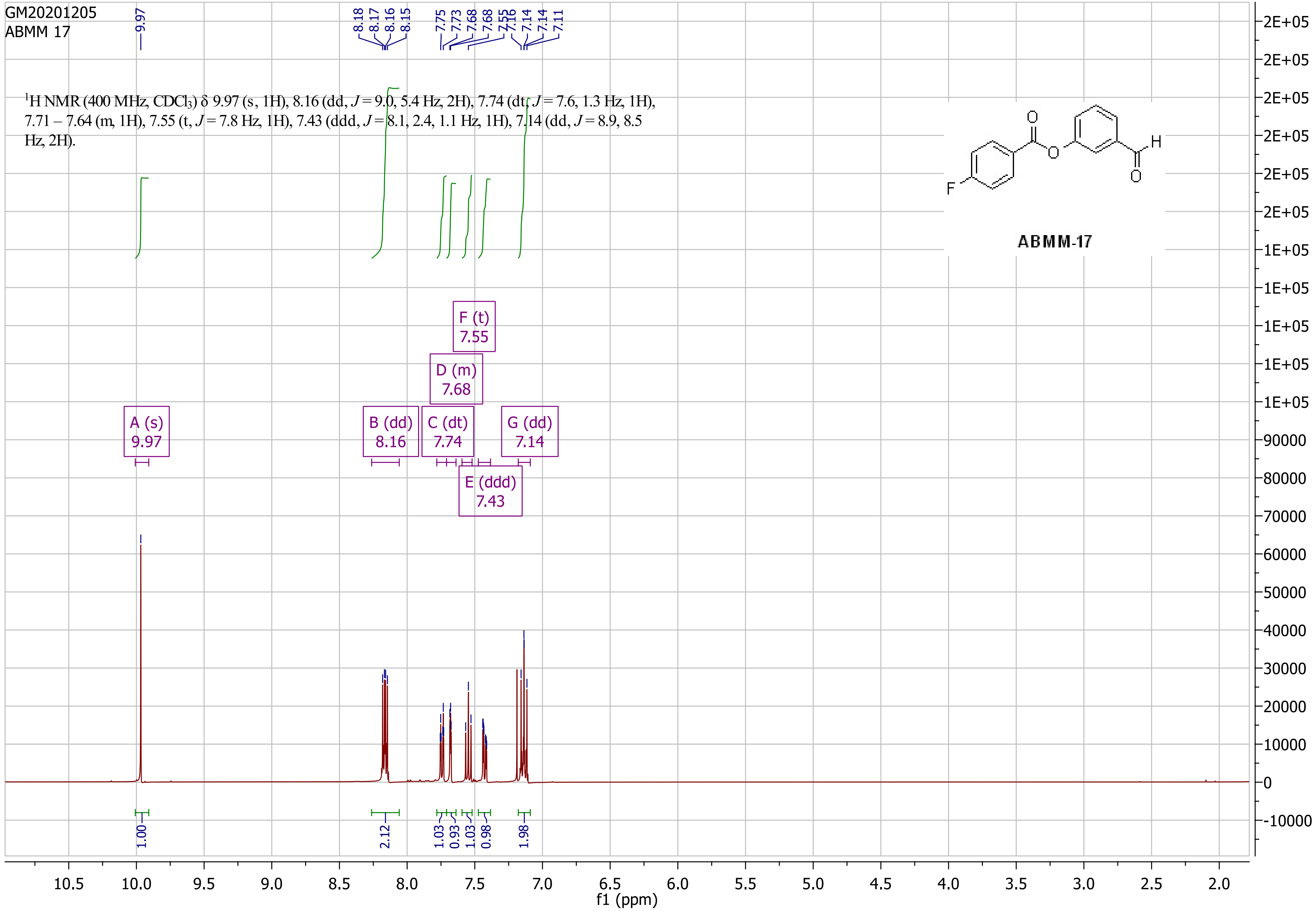
GM20201205
ABMM 17
^{1}H NMR (400 MHz, $CDCl_3$) δ 9.97 (s, 1H), 8.16 (dd, J = 9.0, 5.4 Hz, 2H), 7.74 (dt, J = 7.6, 1.3 Hz, 1H), 7.71 – 7.64 (m, 1H), 7.55 (t, J = 7.8 Hz, 1H), 7.43 (ddd, J = 8.1, 2.4, 1.1 Hz, 1H), 7.14 (dd, J = 8.9, 8.5 Hz, 2H).
ABMM-17
A (s) 9.97
B (dd) 8.16
C (dt) 7.74
D (m) 7.68
E (ddd) 7.43
F (t) 7.55
G (dd) 7.14
9.97
8.18 8.17 8.16 8.15
7.75 7.73 7.68 7.68 7.55 7.16 7.14 7.14 7.11
1.00
2.12
1.03 0.93 1.03 0.98
1.98
f1 (ppm)

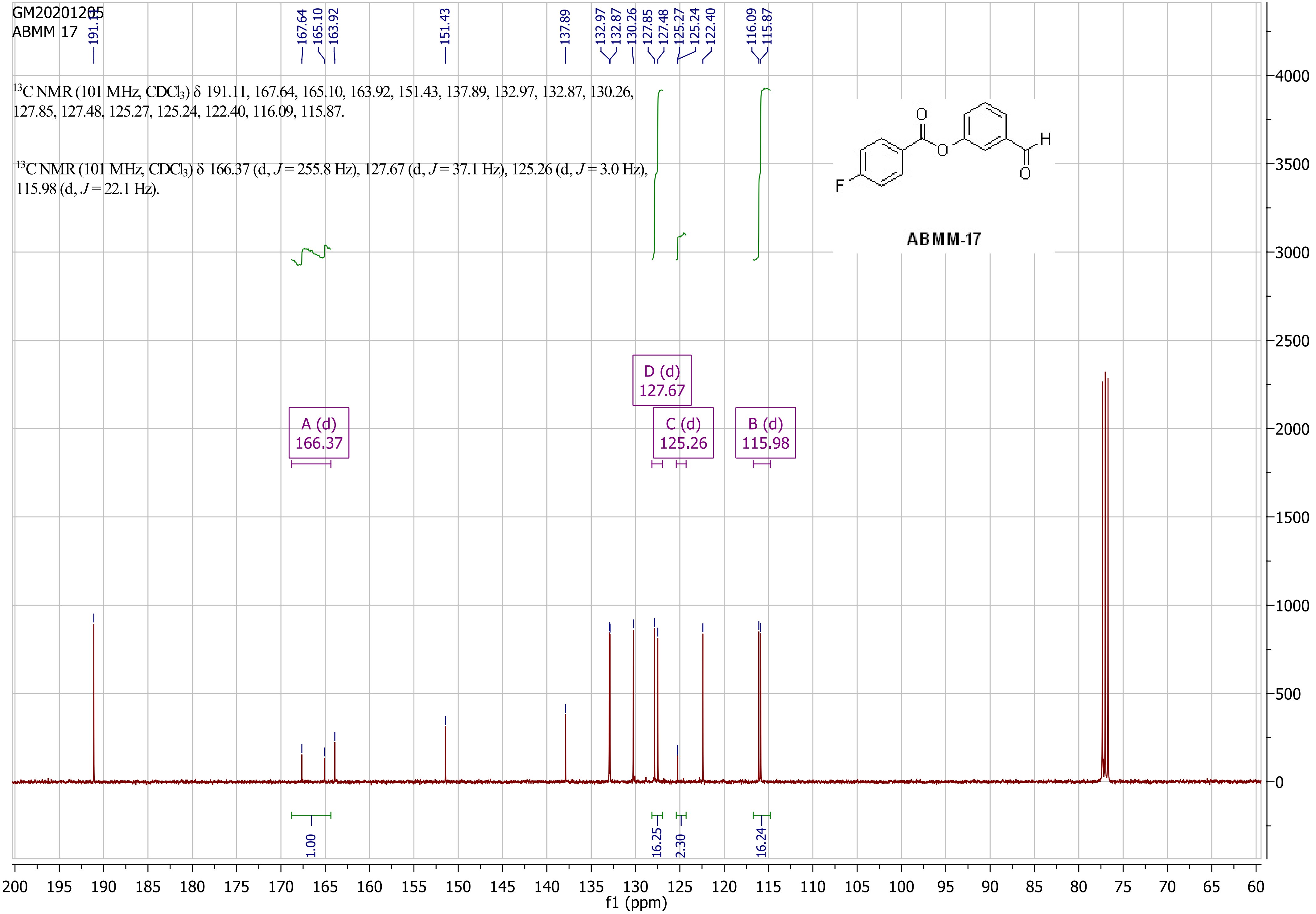
GM20201205
ABMM 17
191.11
167.64
165.10
163.92
151.43
137.89
132.97
132.87
130.26
127.85
127.48
125.27
125.24
122.40
116.09
115.87
^{13}C NMR (101 MHz, CDCl$_3$) δ 191.11, 167.64, 165.10, 163.92, 151.43, 137.89, 132.97, 132.87, 130.26, 127.85, 127.48, 125.27, 125.24, 122.40, 116.09, 115.87.
^{13}C NMR (101 MHz, CDCl$_3$) δ 166.37 (d, J = 255.8 Hz), 127.67 (d, J = 37.1 Hz), 125.26 (d, J = 3.0 Hz), 115.98 (d, J = 22.1 Hz).
ABMM-17
A (d) 166.37
B (d) 115.98
C (d) 125.26
D (d) 127.67
1.00
16.25
2.30
16.24
f1 (ppm)

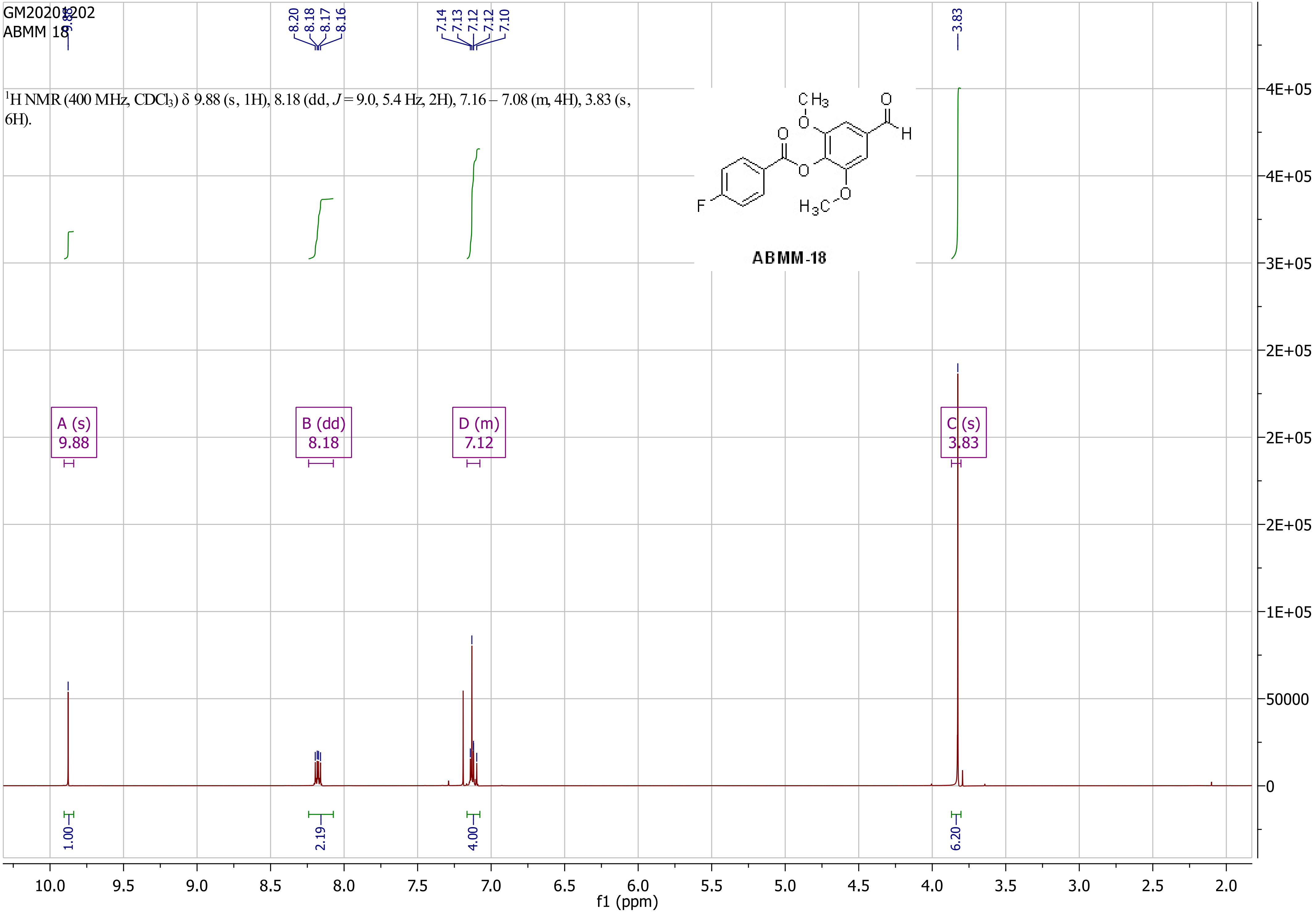
GM20201202
ABMM 18
9.88
8.20
8.18
8.17
8.16
7.14
7.13
7.12
7.12
7.10
3.83
¹H NMR (400 MHz, CDCl3) δ 9.88 (s, 1H), 8.18 (dd, J = 9.0, 5.4 Hz, 2H), 7.16 – 7.08 (m, 4H), 3.83 (s, 6H).
CH3
O
O
H
O
O
F
H3C
O
ABMM-18
A (s)
9.88
B (dd)
8.18
D (m)
7.12
C (s)
3.83
1.00
2.19
4.00
6.20
4E+05
4E+05
3E+05
2E+05
2E+05
2E+05
1E+05
50000
0
10.0
9.5
9.0
8.5
8.0
7.5
7.0
6.5
6.0
5.5
5.0
4.5
4.0
3.5
3.0
2.5
2.0
f1 (ppm)

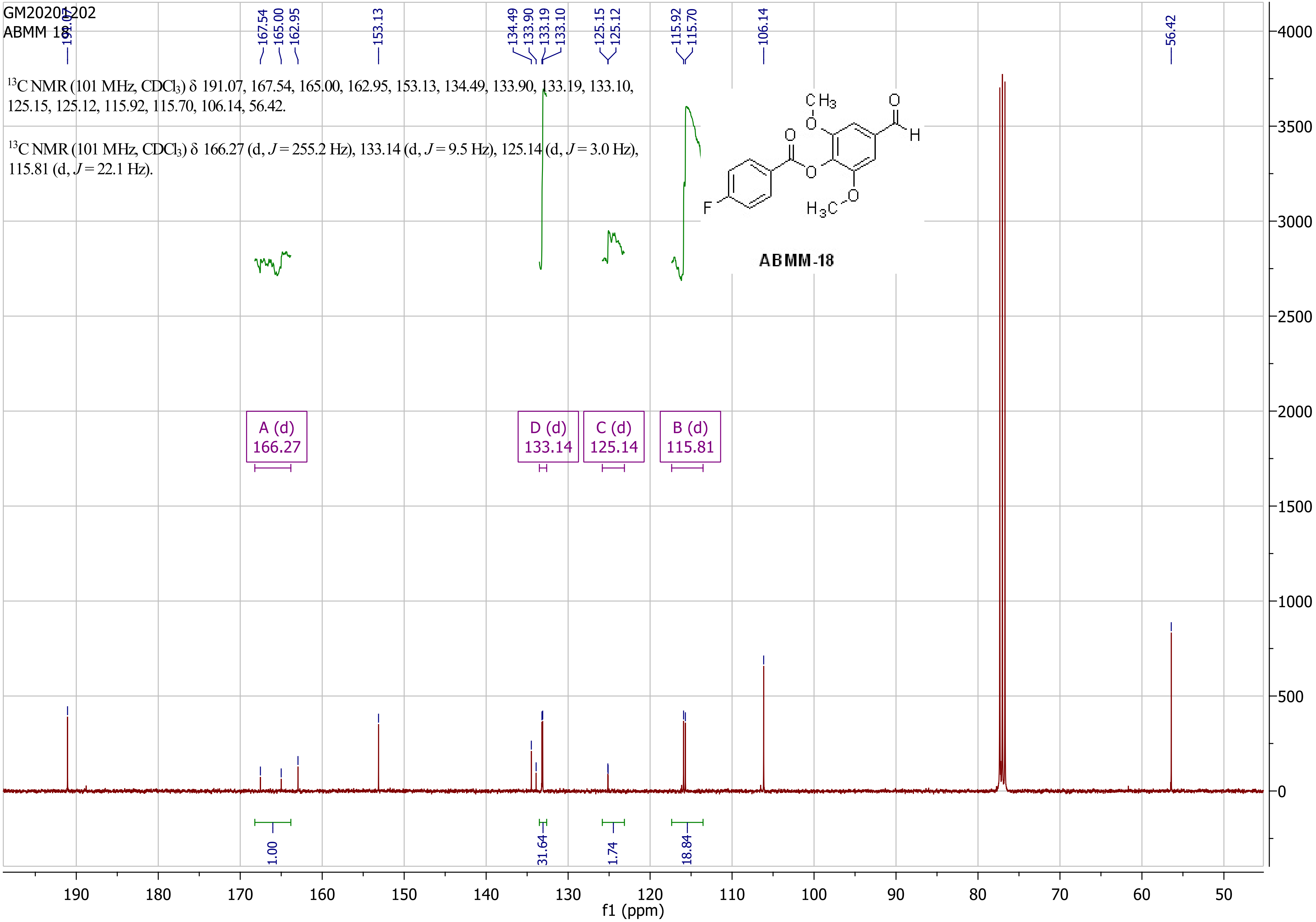

GM20201202
ABMM 18
^{13}C NMR (101 MHz, CDCl$_3$) δ 191.07, 167.54, 165.00, 162.95, 153.13, 134.49, 133.90, 133.19, 133.10, 125.15, 125.12, 115.92, 115.70, 106.14, 56.42.
^{13}C NMR (101 MHz, CDCl$_3$) δ 166.27 (d, J = 255.2 Hz), 133.14 (d, J = 9.5 Hz), 125.14 (d, J = 3.0 Hz), 115.81 (d, J = 22.1 Hz).
A (d) 166.27
D (d) 133.14
C (d) 125.14
B (d) 115.81
1.00
31.64
1.74
18.84
ABMM-18
f1 (ppm)

GM20201129
ABMM 19

^{1}H NMR (400 MHz, $CDCl_3$) δ 8.16 (dd, J = 9.0, 5.4 Hz, 2H), 7.11 (t, J = 8.7 Hz, 2H), 7.03 (d, J = 8.1 Hz, 1H), 6.99 – 6.90 (m, 2H), 6.55 (d, J = 15.9 Hz, 1H), 6.28 (dt, J = 15.9, 5.7 Hz, 1H), 4.28 (td, J = 5.7, 1.4 Hz, 2H), 3.76 (s, 3H), 1.41 (t, J = 5.9 Hz, 1H).

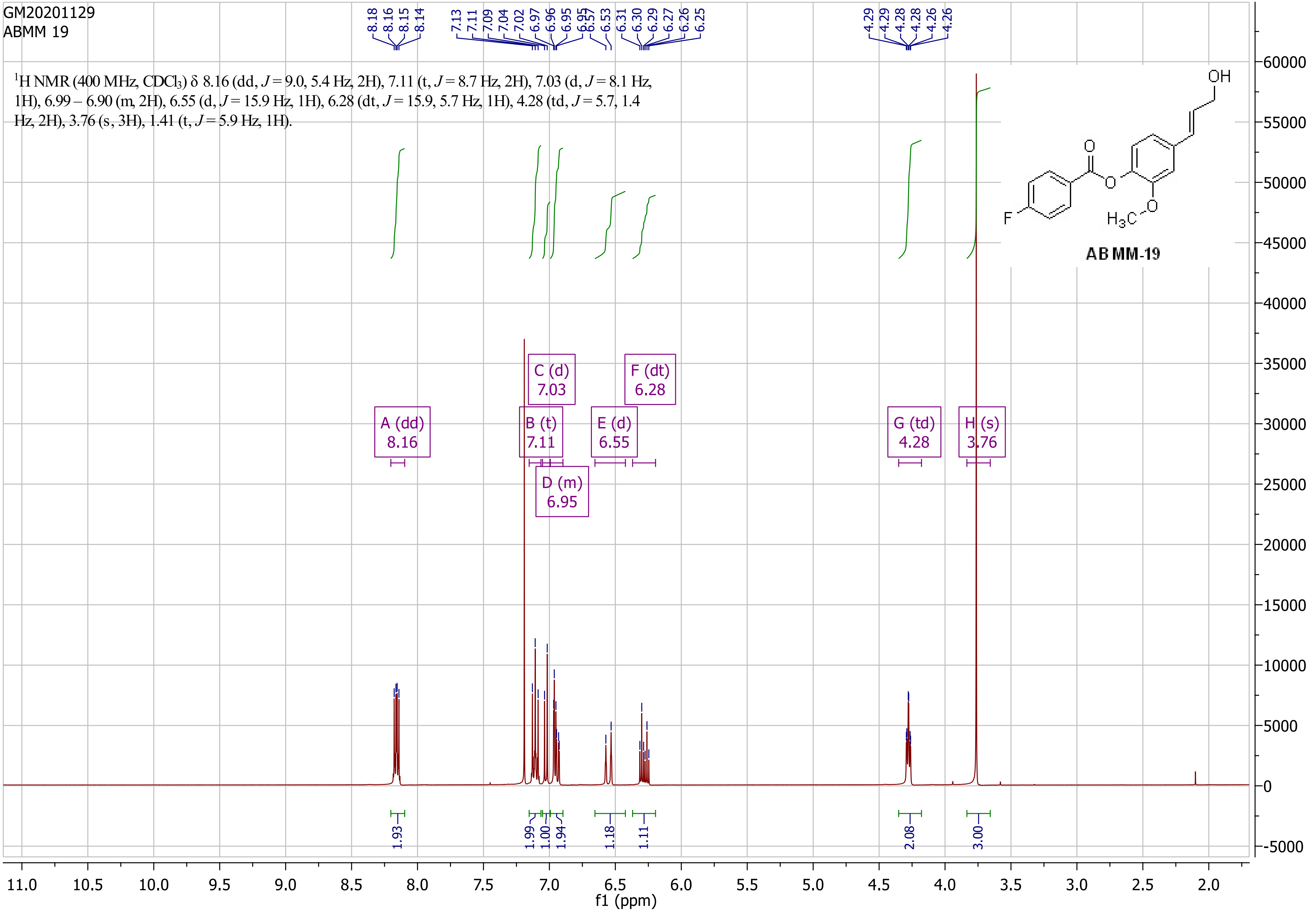

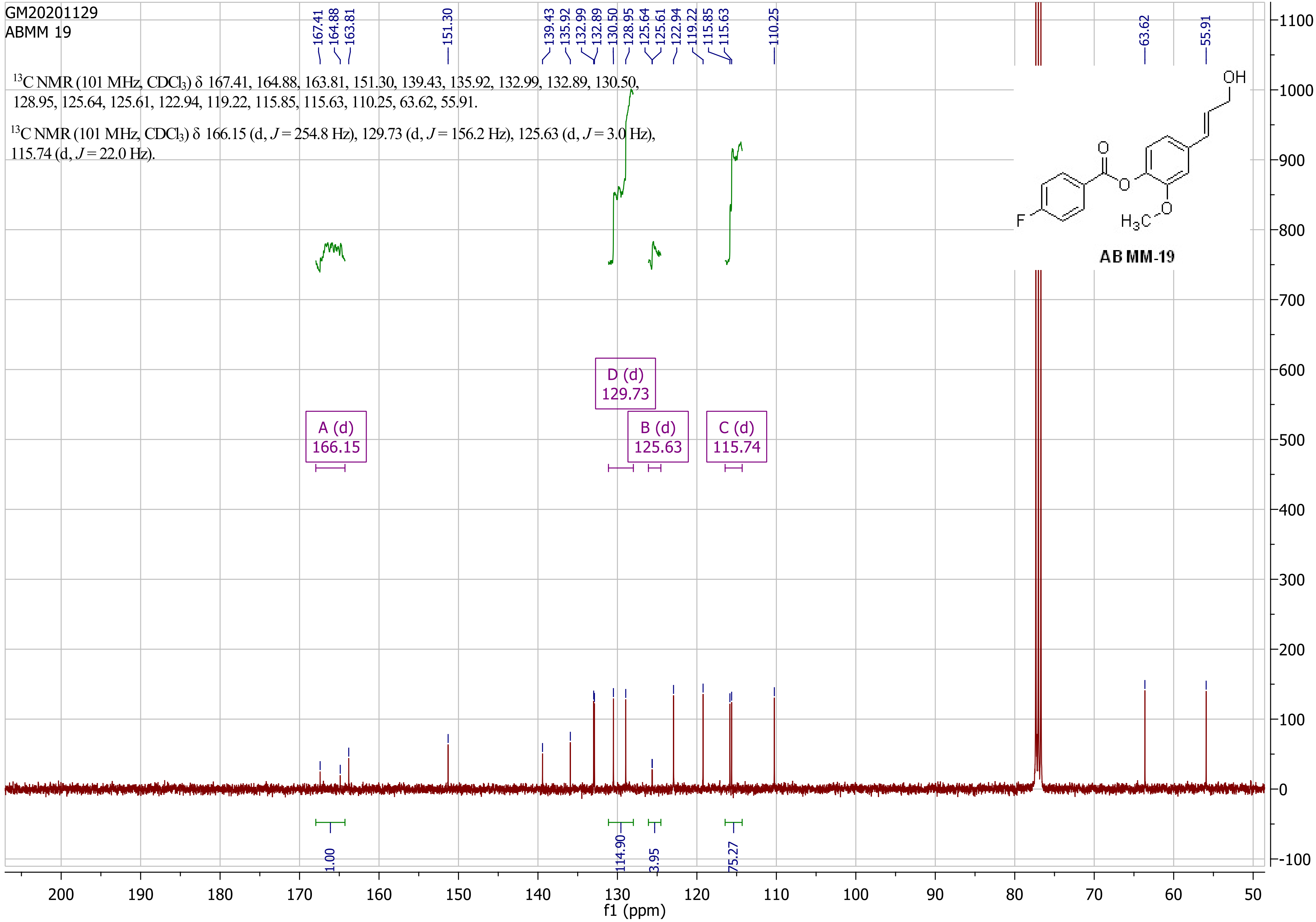
GM20201129
ABMM 19
167.41
164.88
163.81
151.30
139.43
135.92
132.99
132.89
130.50
128.95
125.64
125.61
122.94
119.22
115.85
115.63
110.25
63.62
55.91
13C NMR (101 MHz, CDCl3) δ 167.41, 164.88, 163.81, 151.30, 139.43, 135.92, 132.99, 132.89, 130.50, 128.95, 125.64, 125.61, 122.94, 119.22, 115.85, 115.63, 110.25, 63.62, 55.91.
13C NMR (101 MHz, CDCl3) δ 166.15 (d, J = 254.8 Hz), 129.73 (d, J = 156.2 Hz), 125.63 (d, J = 3.0 Hz), 115.74 (d, J = 22.0 Hz).
OH
O
O
F
H3C
O
AB MM-19
D (d)
129.73
A (d)
166.15
B (d)
125.63
C (d)
115.74
1.00
114.90
3.95
75.27
f1 (ppm)

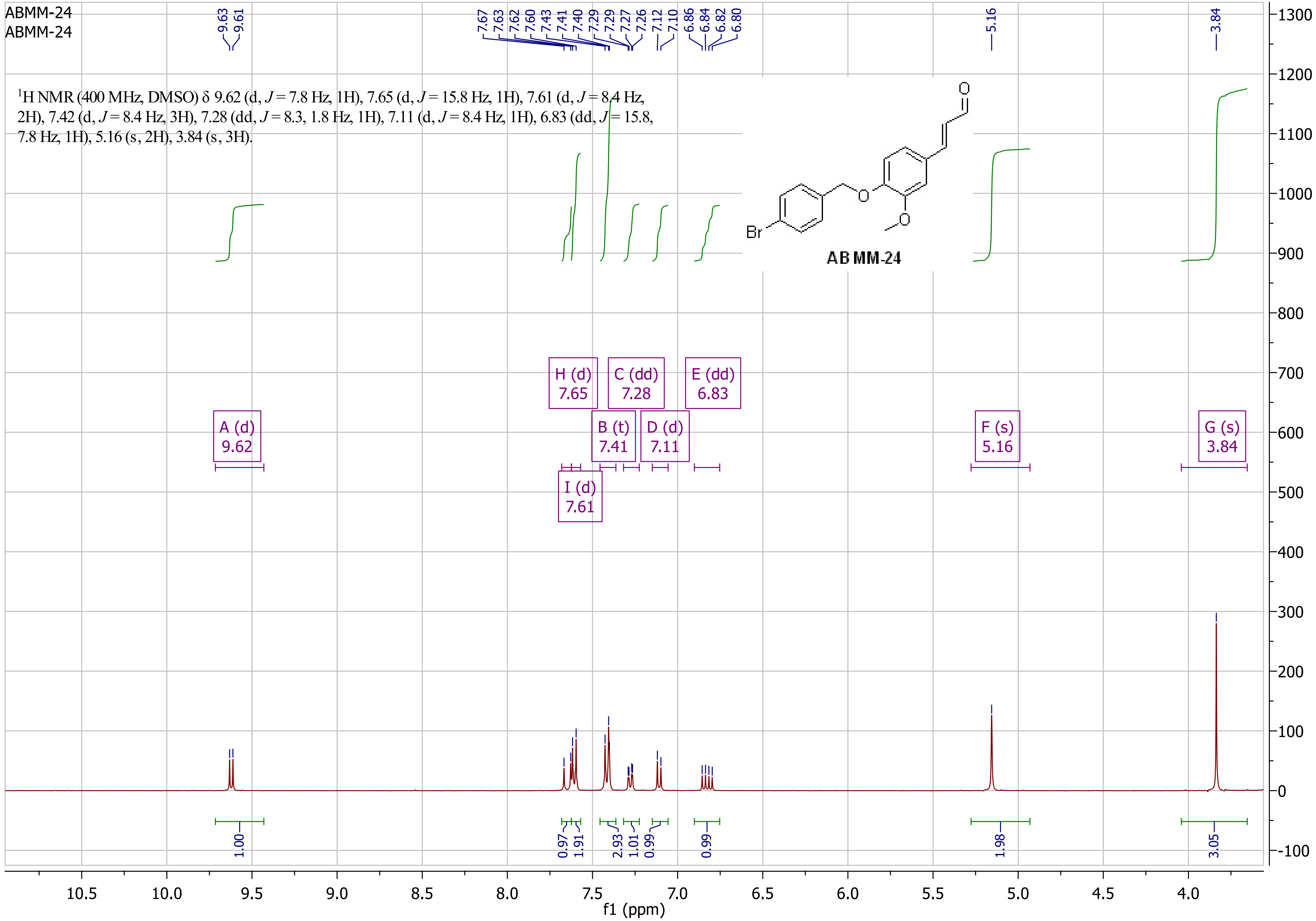
ABMM-24
ABMM-24
^{1}H NMR (400 MHz, DMSO) δ 9.62 (d, J = 7.8 Hz, 1H), 7.65 (d, J = 15.8 Hz, 1H), 7.61 (d, J = 8.4 Hz, 2H), 7.42 (d, J = 8.4 Hz, 3H), 7.28 (dd, J = 8.3, 1.8 Hz, 1H), 7.11 (d, J = 8.4 Hz, 1H), 6.83 (dd, J = 15.8, 7.8 Hz, 1H), 5.16 (s, 2H), 3.84 (s, 3H).
AB MM-24
Br
O
A (d) 9.62
H (d) 7.65
I (d) 7.61
B (t) 7.41
C (dd) 7.28
D (d) 7.11
E (dd) 6.83
F (s) 5.16
G (s) 3.84
f1 (ppm)

^{13}C NMR (101 MHz, DMSO) δ 194.65, 153.95, 150.80, 149.80, 136.61, 131.87, 130.40, 127.85, 127.26, 123.95, 121.58, 113.70, 111.42, 69.48, 56.22.

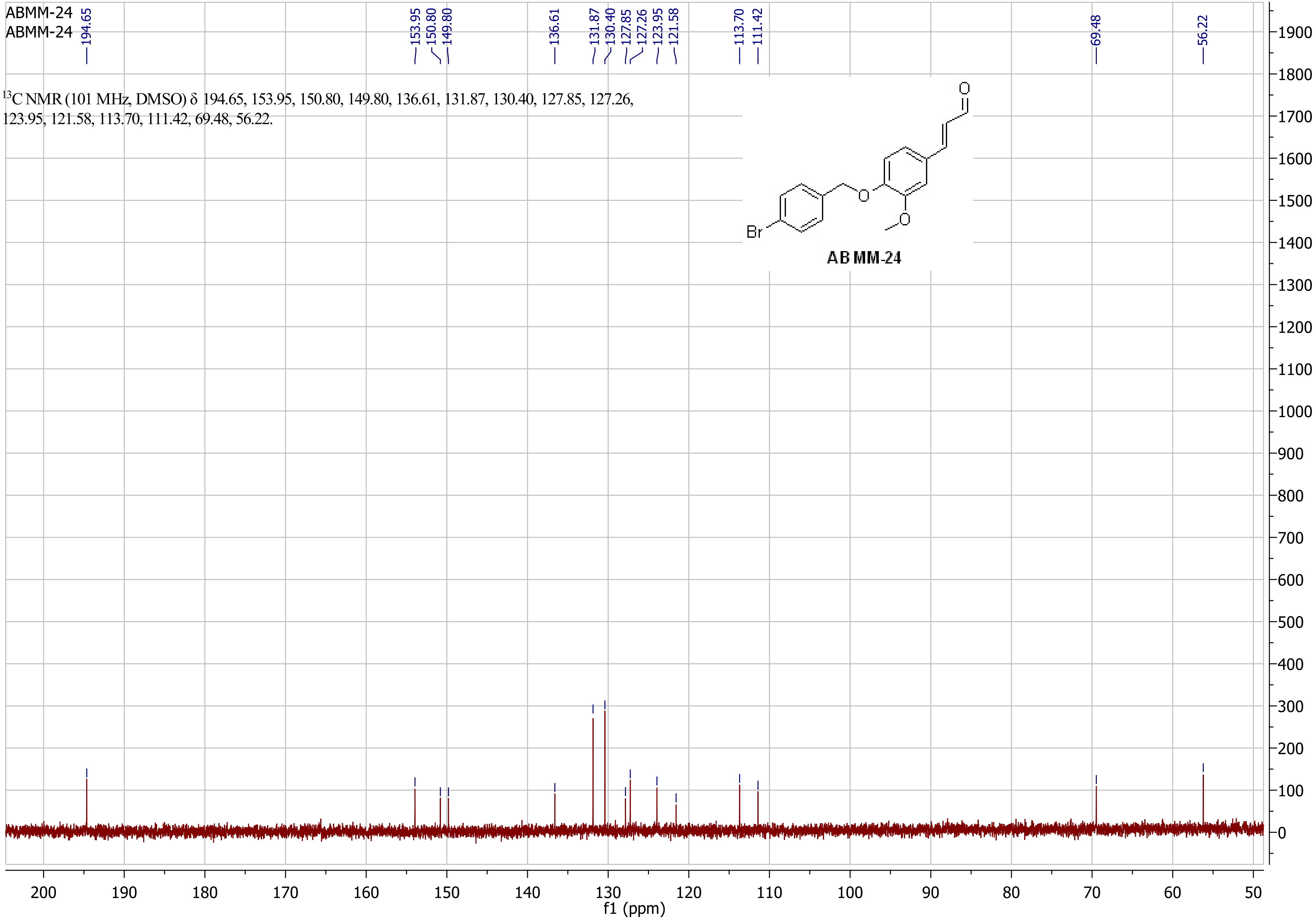

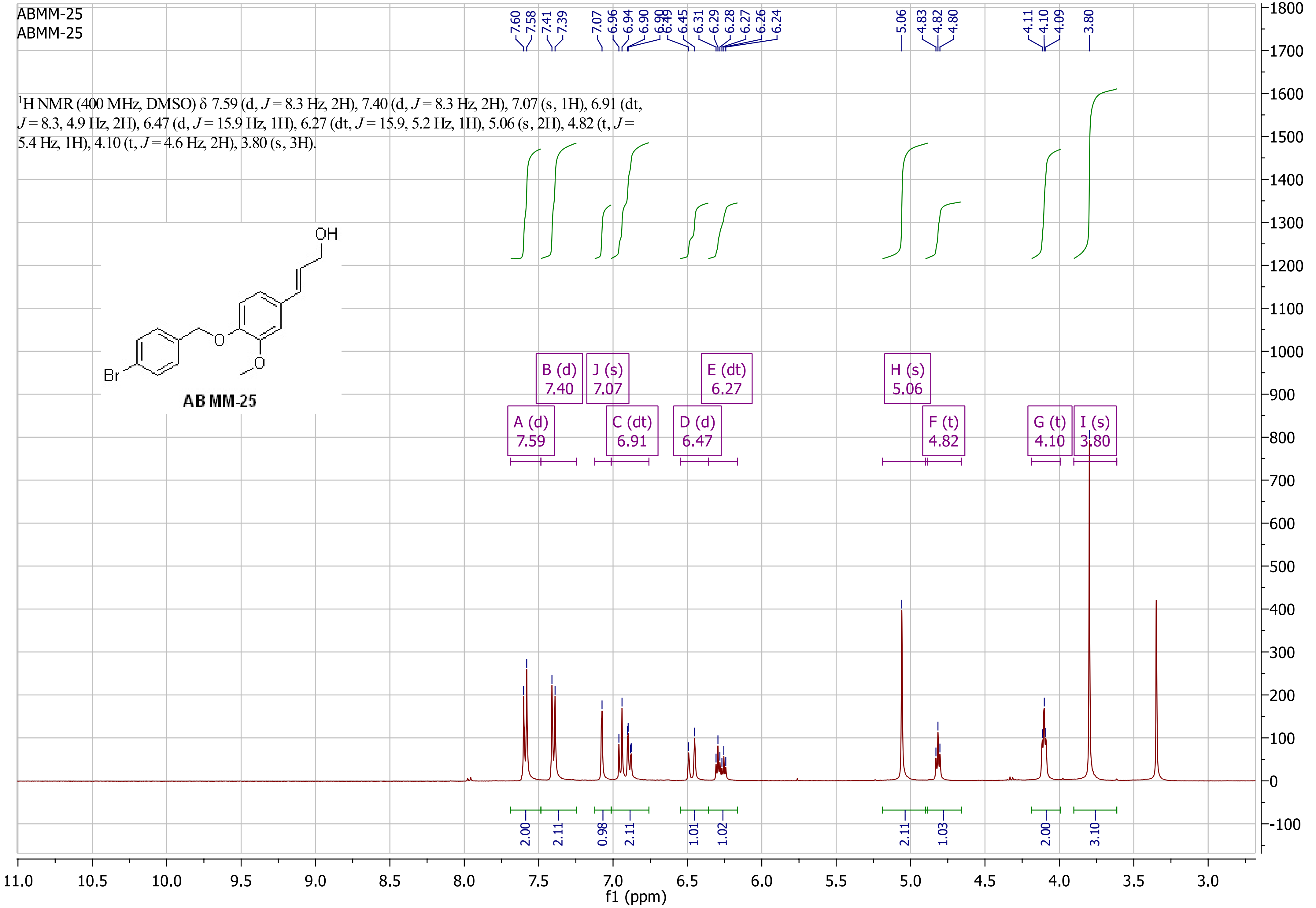
ABMM-25
ABMM-25
^{1}H NMR (400 MHz, DMSO) δ 7.59 (d, J = 8.3 Hz, 2H), 7.40 (d, J = 8.3 Hz, 2H), 7.07 (s, 1H), 6.91 (dt, J = 8.3, 4.9 Hz, 2H), 6.47 (d, J = 15.9 Hz, 1H), 6.27 (dt, J = 15.9, 5.2 Hz, 1H), 5.06 (s, 2H), 4.82 (t, J = 5.4 Hz, 1H), 4.10 (t, J = 4.6 Hz, 2H), 3.80 (s, 3H).
7.60 7.58 7.41 7.39 7.07 6.96 6.94 6.90 6.90 6.49 6.45 6.31 6.29 6.28 6.27 6.26 6.24 5.06 4.83 4.82 4.80 4.11 4.10 4.09 3.80
OH
O
O
Br
AB MM-25
A (d) 7.59
B (d) 7.40
J (s) 7.07
C (dt) 6.91
D (d) 6.47
E (dt) 6.27
H (s) 5.06
F (t) 4.82
G (t) 4.10
I (s) 3.80
2.00 2.11 0.98 2.11 1.01 1.02 2.11 1.03 2.00 3.10
f1 (ppm)

ABMM-25
ABMM-25

^{13}C NMR (101 MHz, DMSO) δ 149.73, 147.45, 137.14, 131.78, 130.96, 130.29, 129.33, 128.89, 121.37, 119.47, 114.22, 109.96, 69.58, 62.06, 56.00.

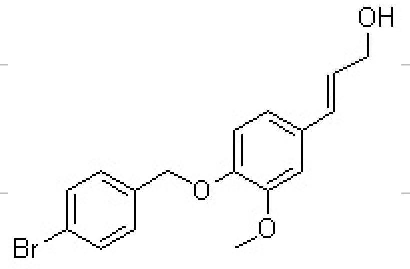

AB MM-25

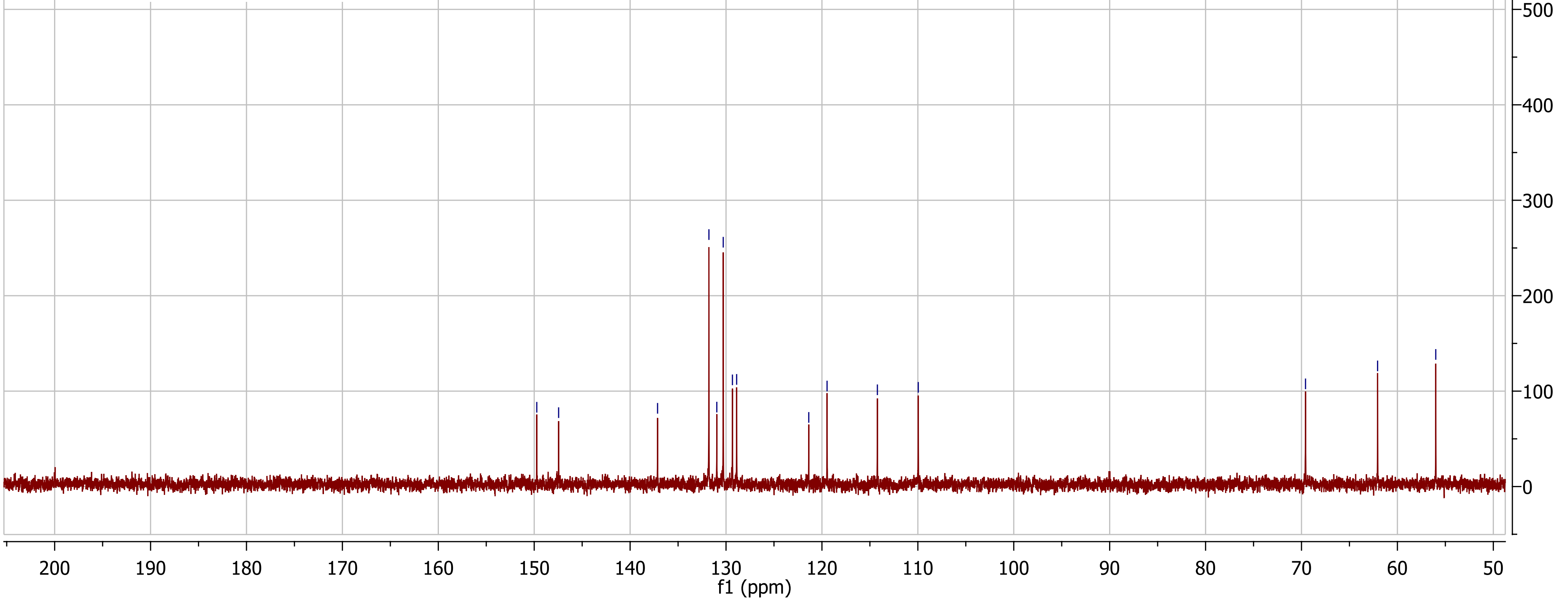

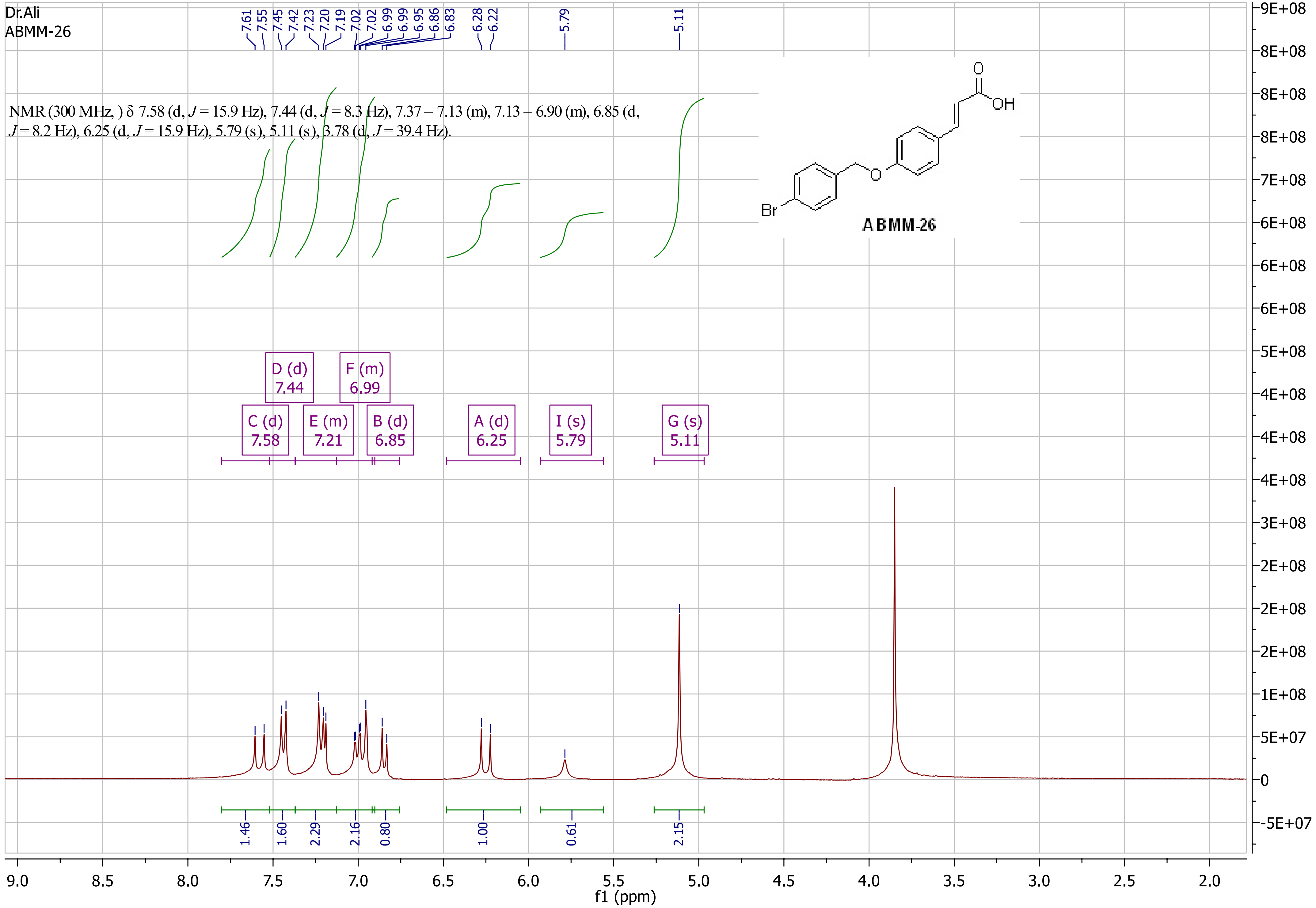
Dr.Ali
ABMM-26
NMR (300 MHz,) δ 7.58 (d, J = 15.9 Hz), 7.44 (d, J = 8.3 Hz), 7.37 – 7.13 (m), 7.13 – 6.90 (m), 6.85 (d, J = 8.2 Hz), 6.25 (d, J = 15.9 Hz), 5.79 (s), 5.11 (s), 3.78 (d, J = 39.4 Hz).
7.61 7.55 7.45 7.42 7.23 7.20 7.19 7.02 7.02 6.99 6.99 6.95 6.86 6.83 6.28 6.22 5.79 5.11
O
OH
O
Br
ABMM-26
D (d) 7.44
F (m) 6.99
C (d) 7.58
E (m) 7.21
B (d) 6.85
A (d) 6.25
I (s) 5.79
G (s) 5.11
1.46 1.60 2.29 2.16 0.80 1.00 0.61 2.15
f1 (ppm)

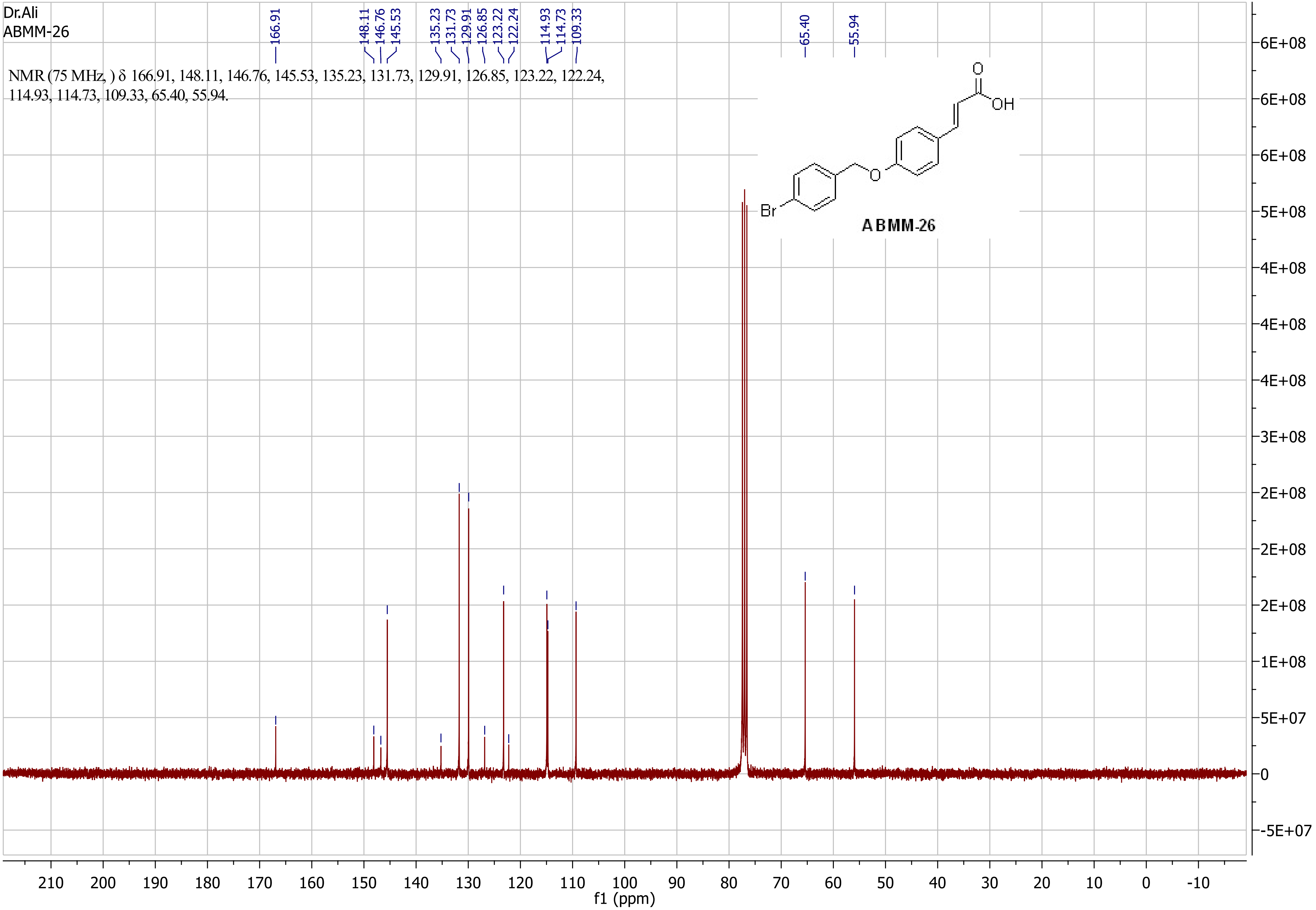
Dr.Ali
ABMM-26
NMR (75 MHz,) δ 166.91, 148.11, 146.76, 145.53, 135.23, 131.73, 129.91, 126.85, 123.22, 122.24, 114.93, 114.73, 109.33, 65.40, 55.94.
166.91
148.11
146.76
145.53
135.23
131.73
129.91
126.85
123.22
122.24
114.93
114.73
109.33
65.40
55.94
O
OH
O
Br
ABMM-26
f1 (ppm)

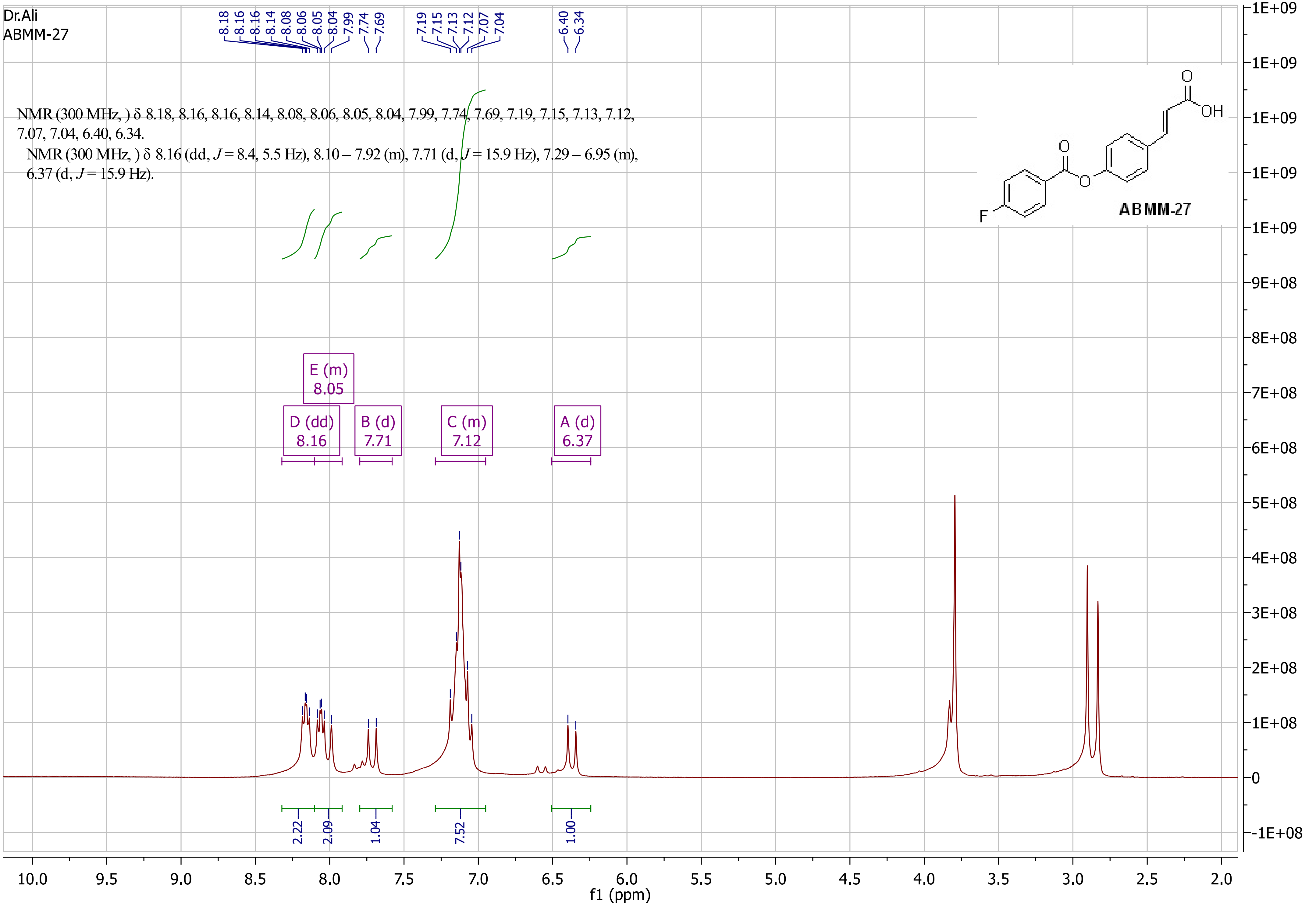
Dr.Ali
ABMM-27
8.18 8.16 8.16 8.14 8.08 8.06 8.05 8.04 7.99 7.74 7.69 7.19 7.15 7.13 7.12 7.07 7.04 6.40 6.34
NMR (300 MHz,) δ 8.18, 8.16, 8.16, 8.14, 8.08, 8.06, 8.05, 8.04, 7.99, 7.74, 7.69, 7.19, 7.15, 7.13, 7.12, 7.07, 7.04, 6.40, 6.34.
NMR (300 MHz,) δ 8.16 (dd, J = 8.4, 5.5 Hz), 8.10 – 7.92 (m), 7.71 (d, J = 15.9 Hz), 7.29 – 6.95 (m), 6.37 (d, J = 15.9 Hz).
F
O
OH
ABMM-27
E (m)
8.05
D (dd)
8.16
B (d)
7.71
C (m)
7.12
A (d)
6.37
2.22
2.09
1.04
7.52
1.00
f1 (ppm)

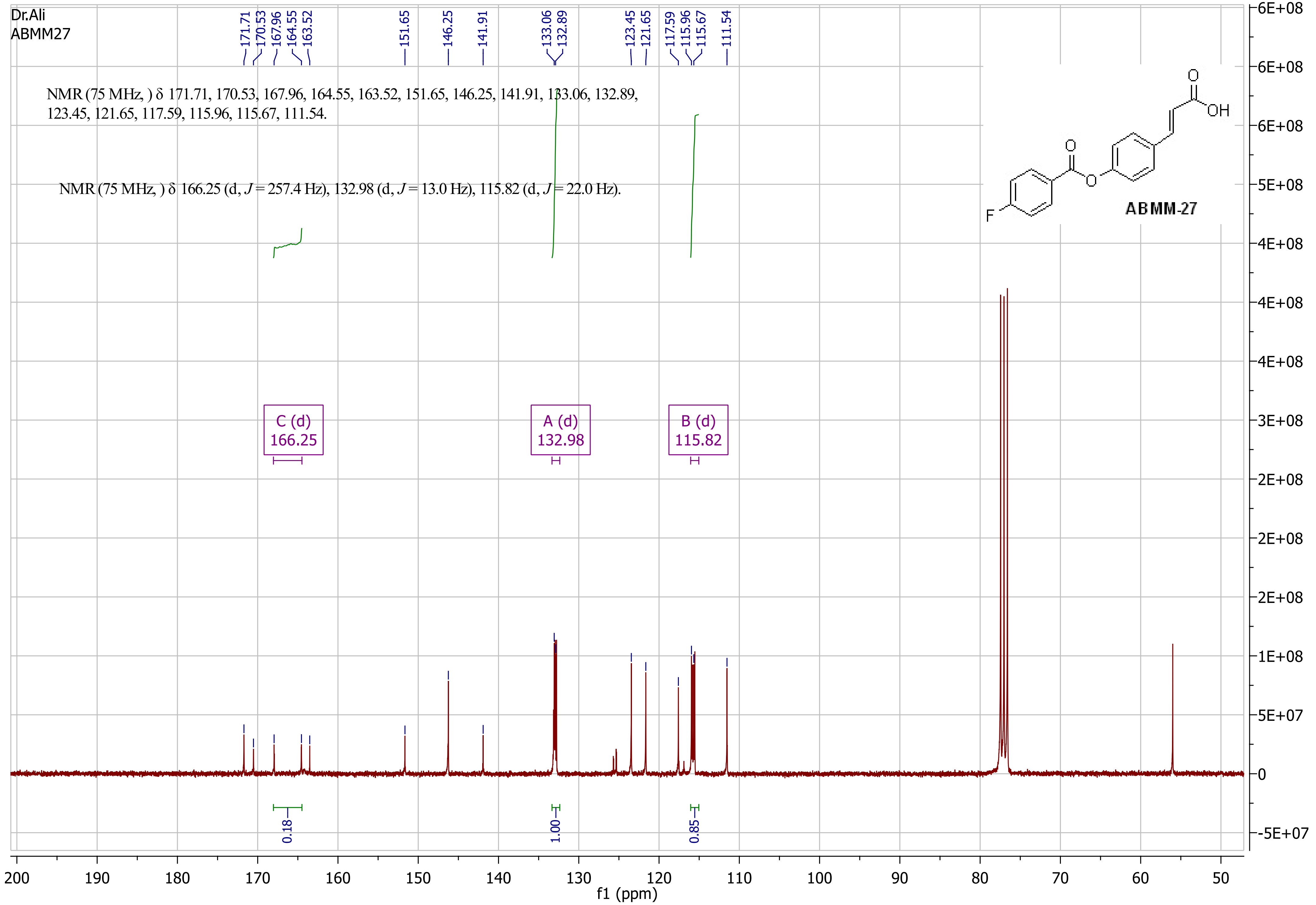
Dr.Ali
ABMM27
171.71
170.53
167.96
164.55
163.52
151.65
146.25
141.91
133.06
132.89
123.45
121.65
117.59
115.96
115.67
111.54
NMR (75 MHz,) δ 171.71, 170.53, 167.96, 164.55, 163.52, 151.65, 146.25, 141.91, 133.06, 132.89, 123.45, 121.65, 117.59, 115.96, 115.67, 111.54.
NMR (75 MHz,) δ 166.25 (d, J = 257.4 Hz), 132.98 (d, J = 13.0 Hz), 115.82 (d, J = 22.0 Hz).
O
OH
O
O
F
ABMM-27
C (d)
166.25
A (d)
132.98
B (d)
115.82
0.18
1.00
0.85
6E+08
5E+08
4E+08
3E+08
2E+08
1E+08
5E+07
0
-5E+07
200
190
180
170
160
150
140
130
120
110
100
90
80
70
60
50
f1 (ppm)

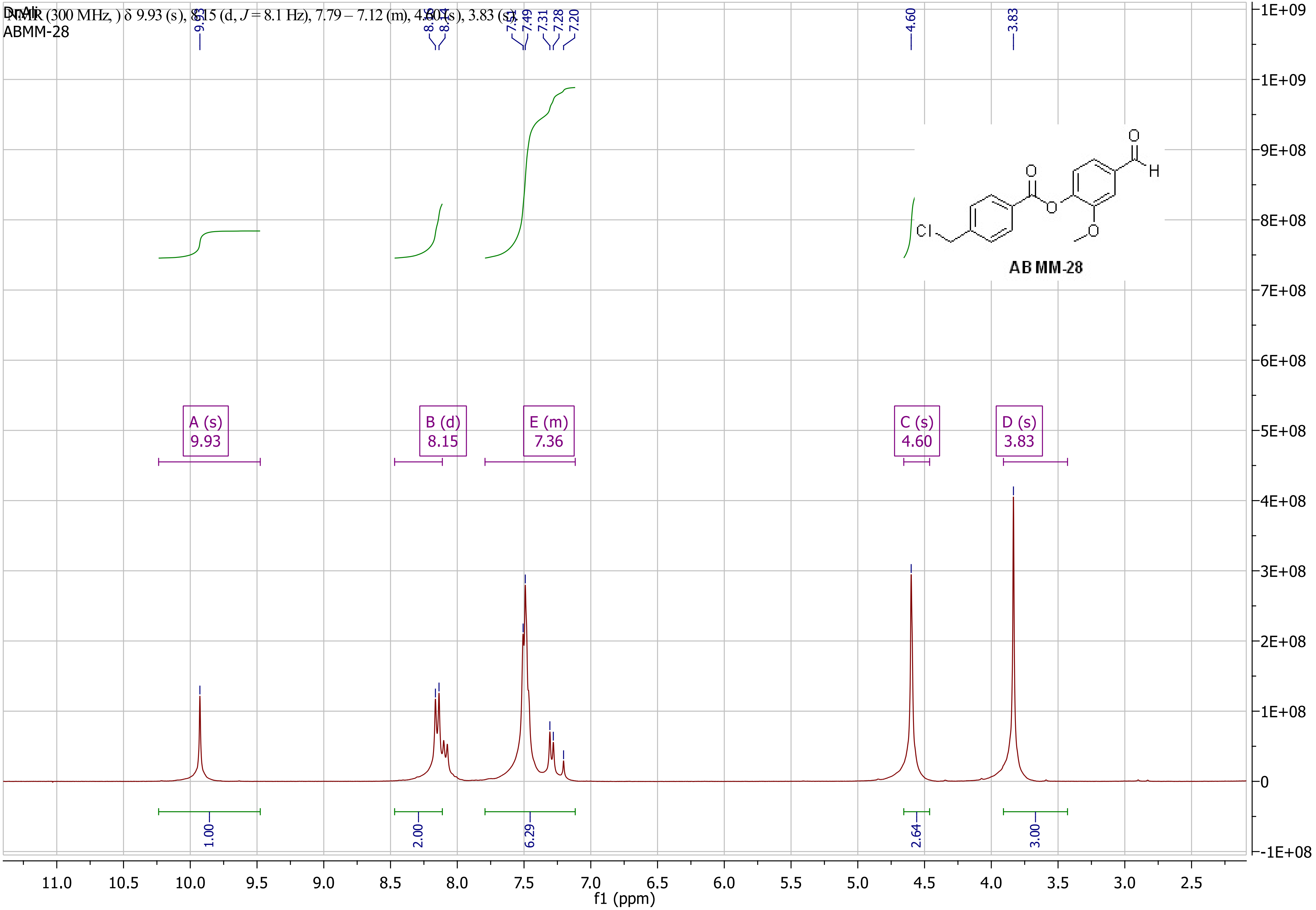

DrAli
1H NMR (300 MHz,) δ 9.93 (s), 8.15 (d, J = 8.1 Hz), 7.79 – 7.12 (m), 4.60 (s), 3.83 (s).
ABMM-28
9.93
8.16
8.14
7.51
7.49
7.31
7.28
7.20
4.60
3.83
AB MM-28
A (s) 9.93
B (d) 8.15
E (m) 7.36
C (s) 4.60
D (s) 3.83
1.00
2.00
6.29
2.64
3.00
f1 (ppm)

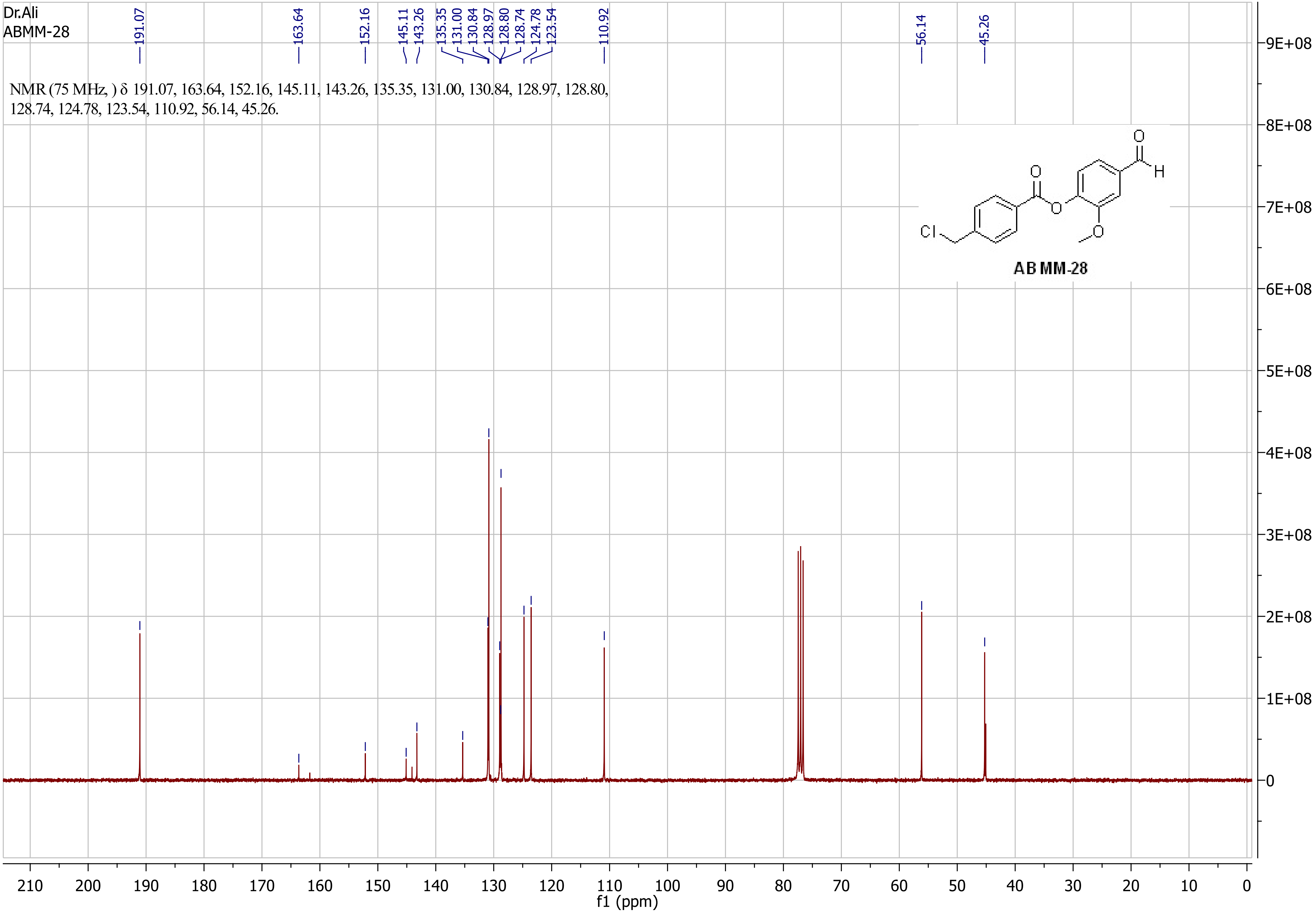

Dr.Ali
ABMM-28
191.07
163.64
152.16
145.11
143.26
135.35
131.00
130.84
128.97
128.80
128.74
124.78
123.54
110.92
56.14
45.26
NMR (75 MHz,) δ 191.07, 163.64, 152.16, 145.11, 143.26, 135.35, 131.00, 130.84, 128.97, 128.80, 128.74, 124.78, 123.54, 110.92, 56.14, 45.26.
AB MM-28
f1 (ppm)

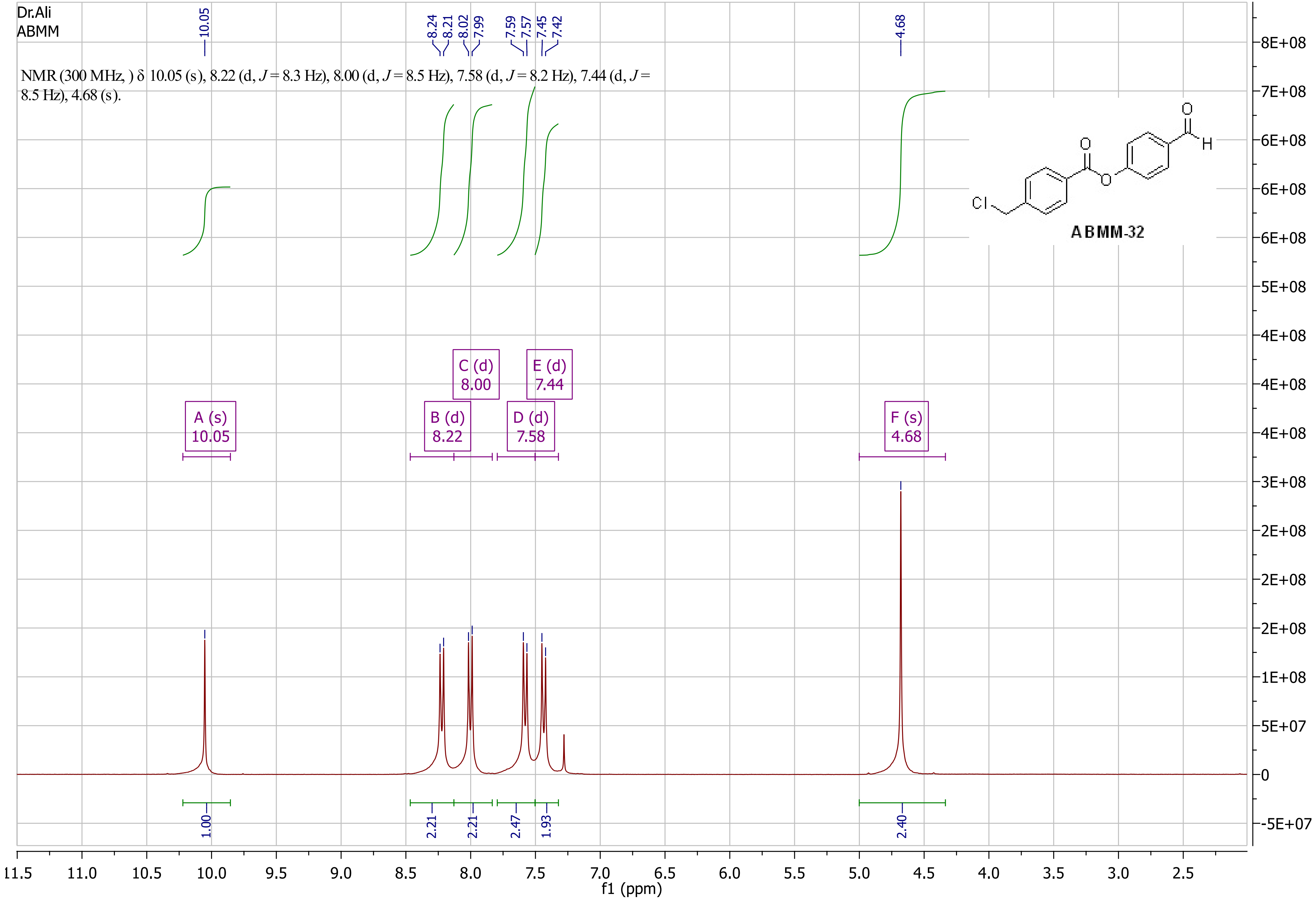
Dr.Ali
ABMM
NMR (300 MHz,) δ 10.05 (s), 8.22 (d, J = 8.3 Hz), 8.00 (d, J = 8.5 Hz), 7.58 (d, J = 8.2 Hz), 7.44 (d, J = 8.5 Hz), 4.68 (s).
10.05
8.24
8.21
8.02
7.99
7.59
7.57
7.45
7.42
4.68
A (s) 10.05
B (d) 8.22
C (d) 8.00
D (d) 7.58
E (d) 7.44
F (s) 4.68
1.00
2.21
2.21
2.47
1.93
2.40
Cl
O
O
O
H
ABMM-32
8E+08
7E+08
6E+08
6E+08
6E+08
5E+08
4E+08
4E+08
4E+08
3E+08
2E+08
2E+08
2E+08
1E+08
5E+07
0
-5E+07
11.5
11.0
10.5
10.0
9.5
9.0
8.5
8.0
7.5
7.0
6.5
6.0
5.5
5.0
4.5
4.0
3.5
3.0
2.5
f1 (ppm)

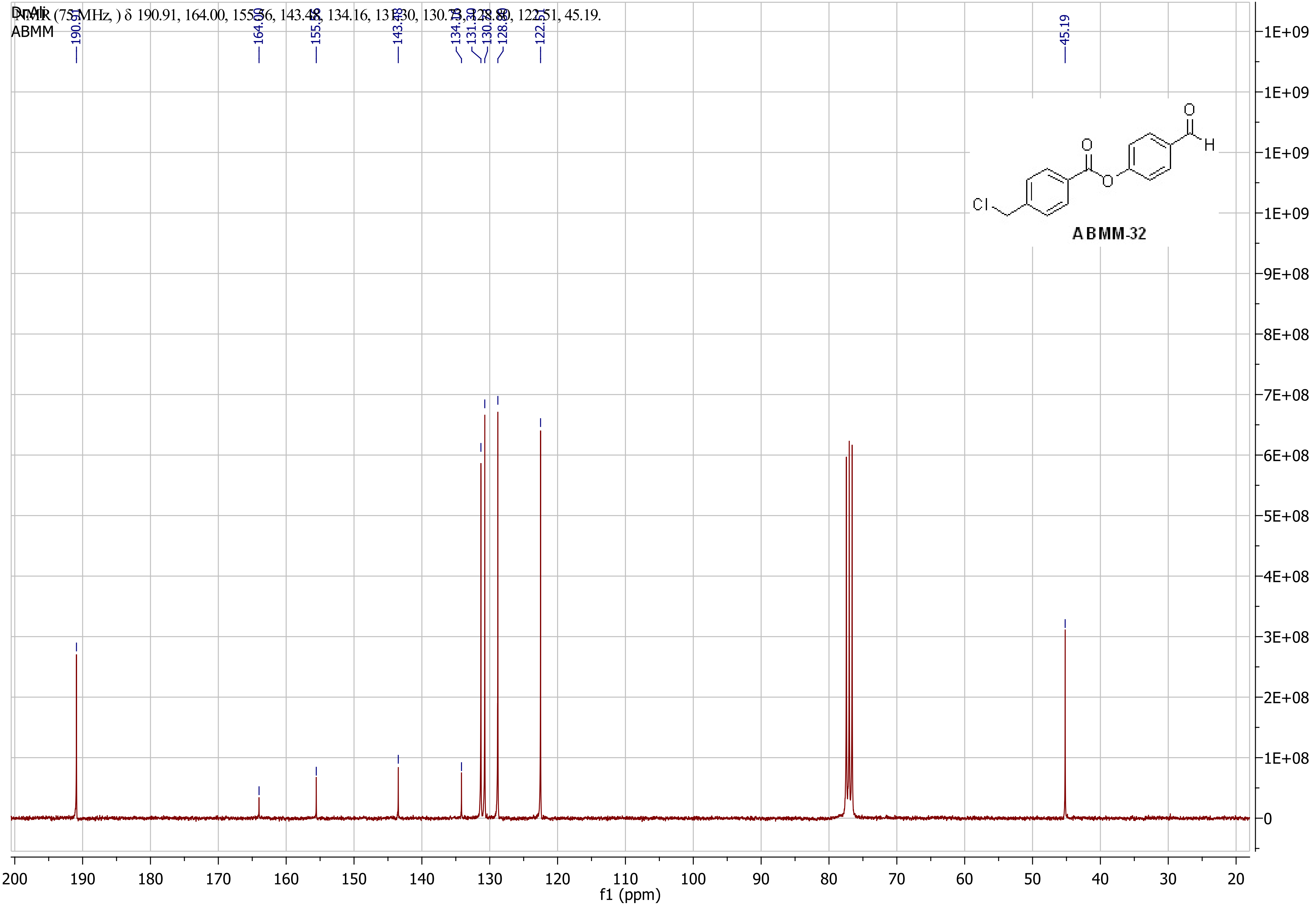
DrAli
NMR (75 MHz,) δ 190.91, 164.00, 155.56, 143.48, 134.16, 131.30, 130.73, 128.80, 122.51, 45.19.
ABMM
190.91
164.00
155.56
143.48
134.16
131.30
130.73
128.80
122.51
45.19
ABMM-32
Cl
O
H
f1 (ppm)

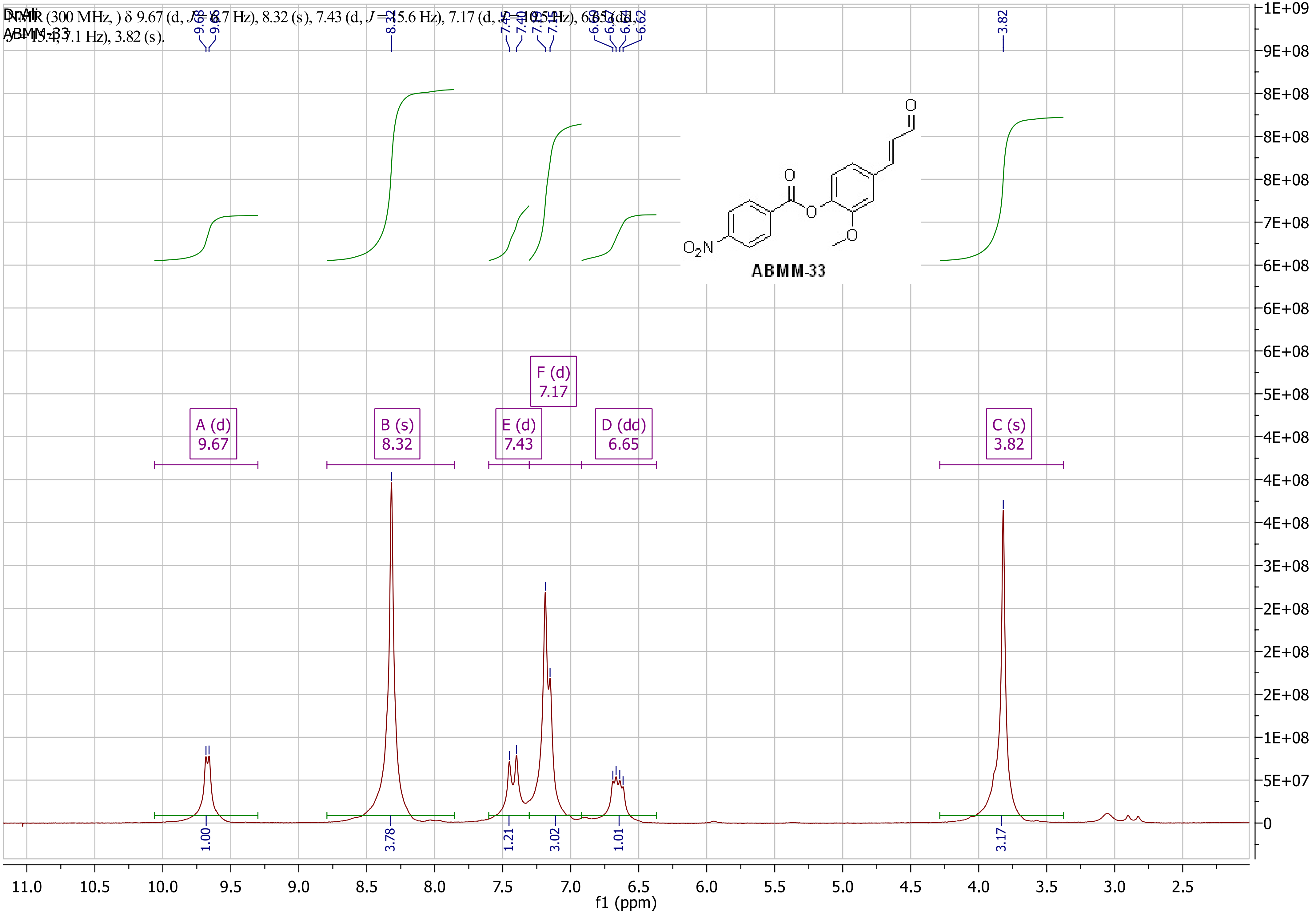
DrAli
ABMM-33
NMR (300 MHz,) δ 9.67 (d, J = 6.7 Hz), 8.32 (s), 7.43 (d, J = 15.6 Hz), 7.17 (d, J = 10.5 Hz), 6.65 (dd, J = 15.4, 7.1 Hz), 3.82 (s).
ABMM-33
O2N
A (d) 9.67
B (s) 8.32
E (d) 7.43
F (d) 7.17
D (dd) 6.65
C (s) 3.82
1.00
3.78
1.21
3.02
1.01
3.17
f1 (ppm)

Dr.Ali
ABMM-33

NMR (75 MHz,) δ 193.35, 162.57, 151.54, 151.00, 141.85, 134.41, 133.49, 131.49, 129.06, 123.74, 123.37, 121.88, 111.54, 56.04.

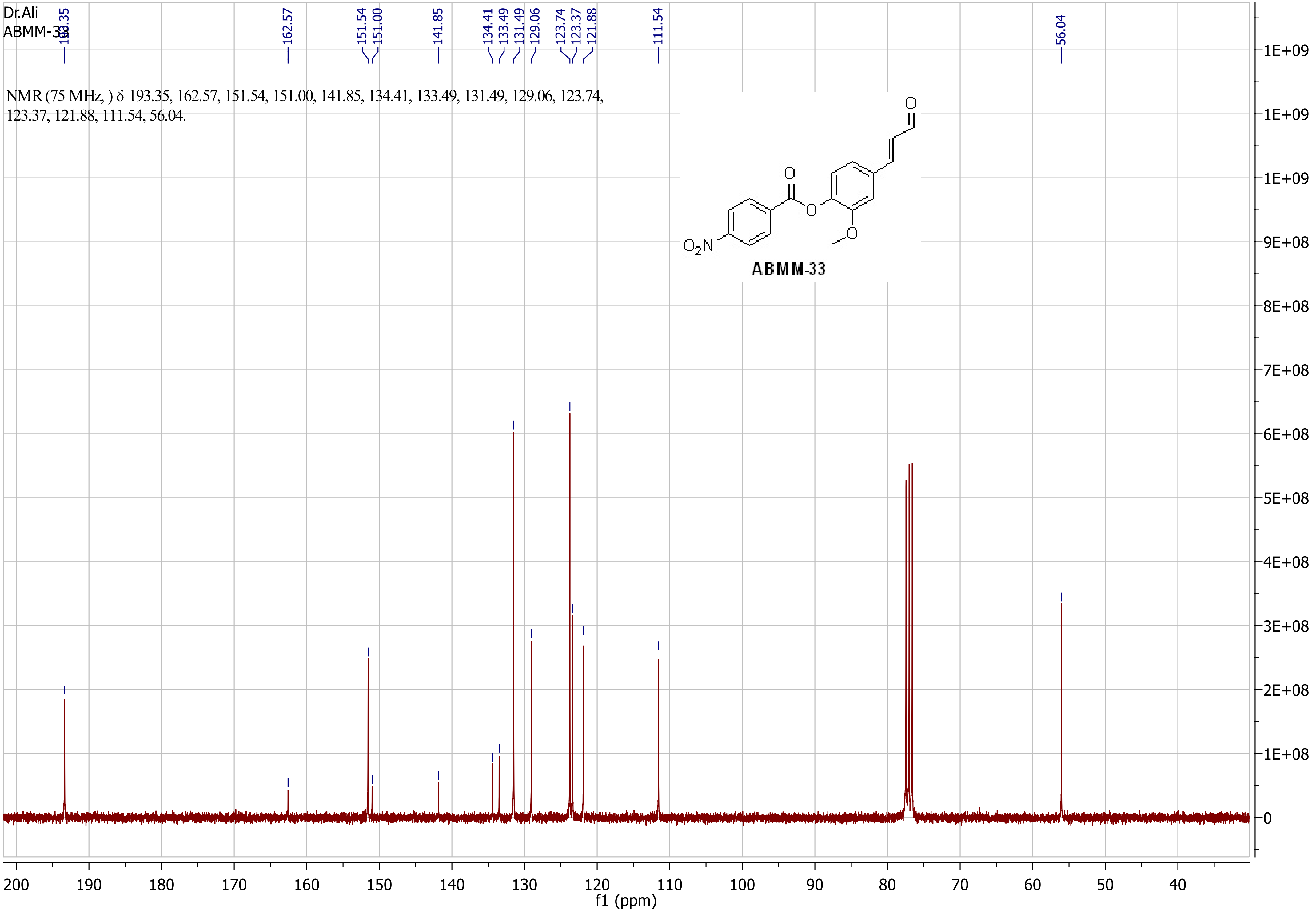

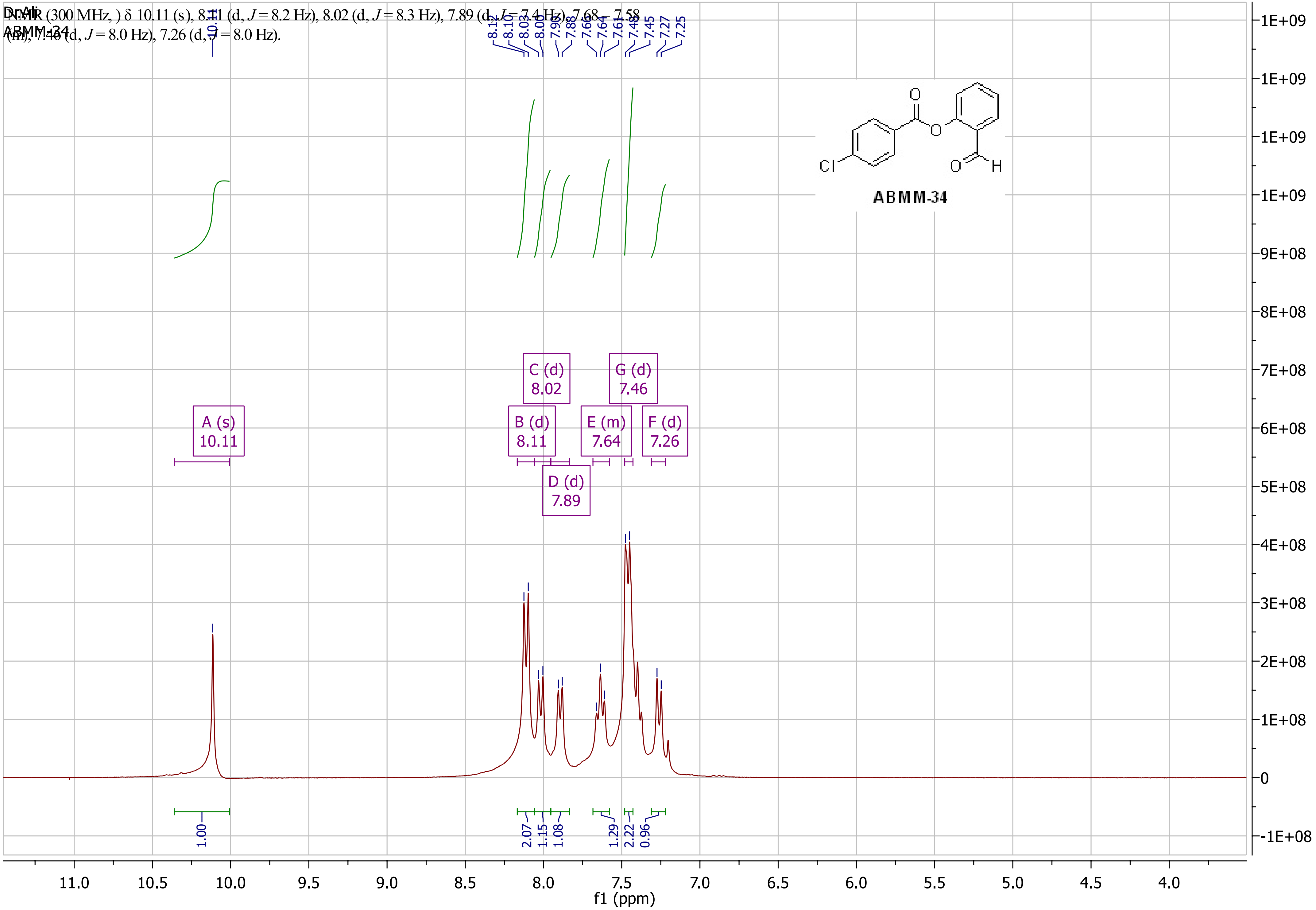
DrAli
ABMM-34
NMR (300 MHz,) δ 10.11 (s), 8.11 (d, J = 8.2 Hz), 8.02 (d, J = 8.3 Hz), 7.89 (d, J = 7.4 Hz), 7.68 – 7.58 (m), 7.46 (d, J = 8.0 Hz), 7.26 (d, J = 8.0 Hz).
10.11
8.12
8.10
8.03
8.00
7.90
7.88
7.66
7.64
7.61
7.48
7.45
7.27
7.25
Cl
O
O
O
H
ABMM-34
A (s)
10.11
B (d)
8.11
C (d)
8.02
D (d)
7.89
E (m)
7.64
G (d)
7.46
F (d)
7.26
1.00
2.07
1.15
1.08
1.29
2.22
0.96
1E+09
9E+08
8E+08
7E+08
6E+08
5E+08
4E+08
3E+08
2E+08
1E+08
0
-1E+08
11.0
10.5
10.0
9.5
9.0
8.5
8.0
7.5
7.0
6.5
6.0
5.5
5.0
4.5
4.0
f1 (ppm)

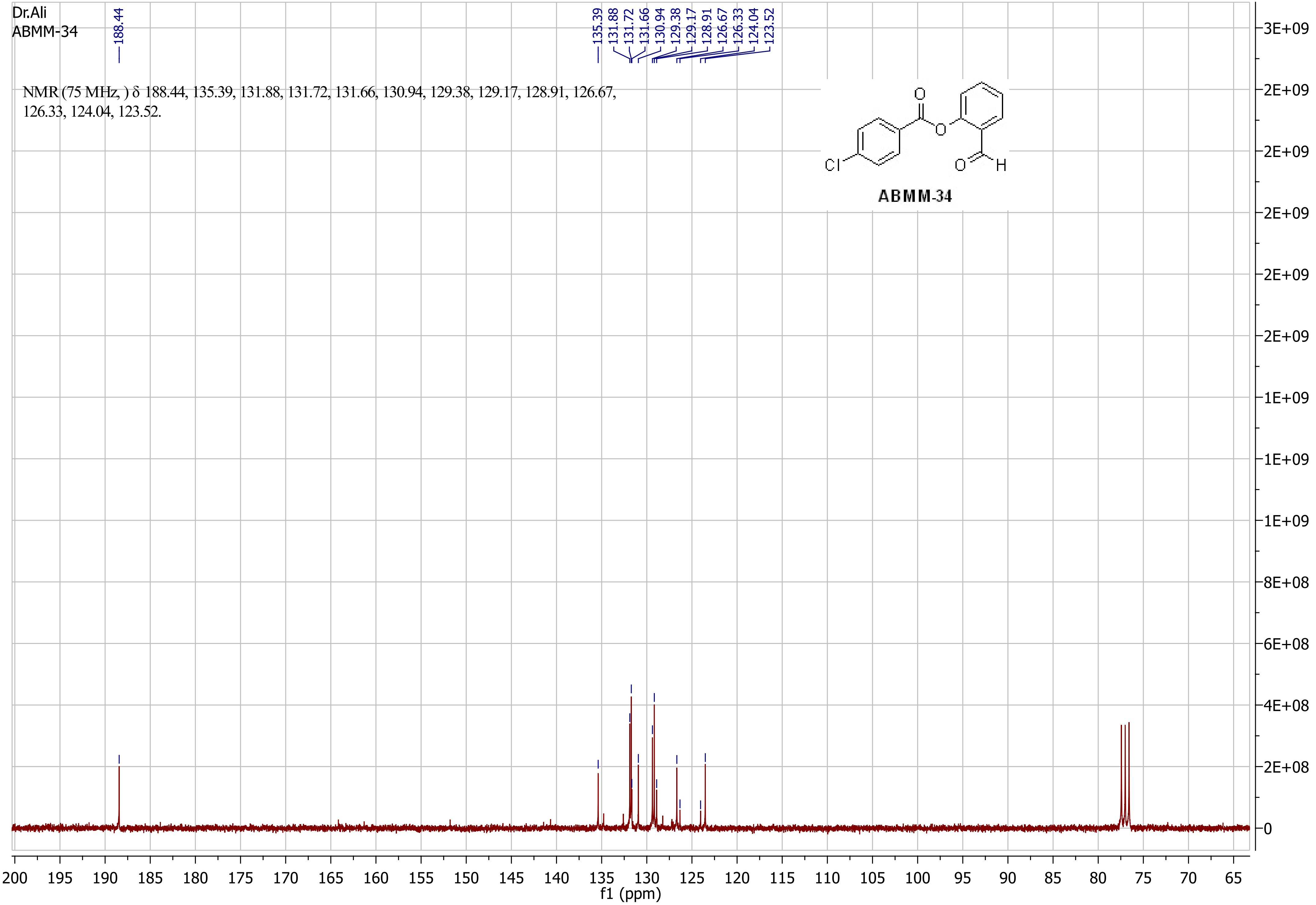
Dr.Ali
ABMM-34
188.44
135.39
131.88
131.72
131.66
130.94
129.38
129.17
128.91
126.67
126.33
124.04
123.52
NMR (75 MHz,) δ 188.44, 135.39, 131.88, 131.72, 131.66, 130.94, 129.38, 129.17, 128.91, 126.67, 126.33, 124.04, 123.52.
O
O
Cl
O
H
ABMM-34
f1 (ppm)
200 195 190 185 180 175 170 165 160 155 150 145 140 135 130 125 120 115 110 105 100 95 90 85 80 75 70 65
3E+09
2E+09
2E+09
2E+09
2E+09
2E+09
1E+09
1E+09
1E+09
8E+08
6E+08
4E+08
2E+08
0

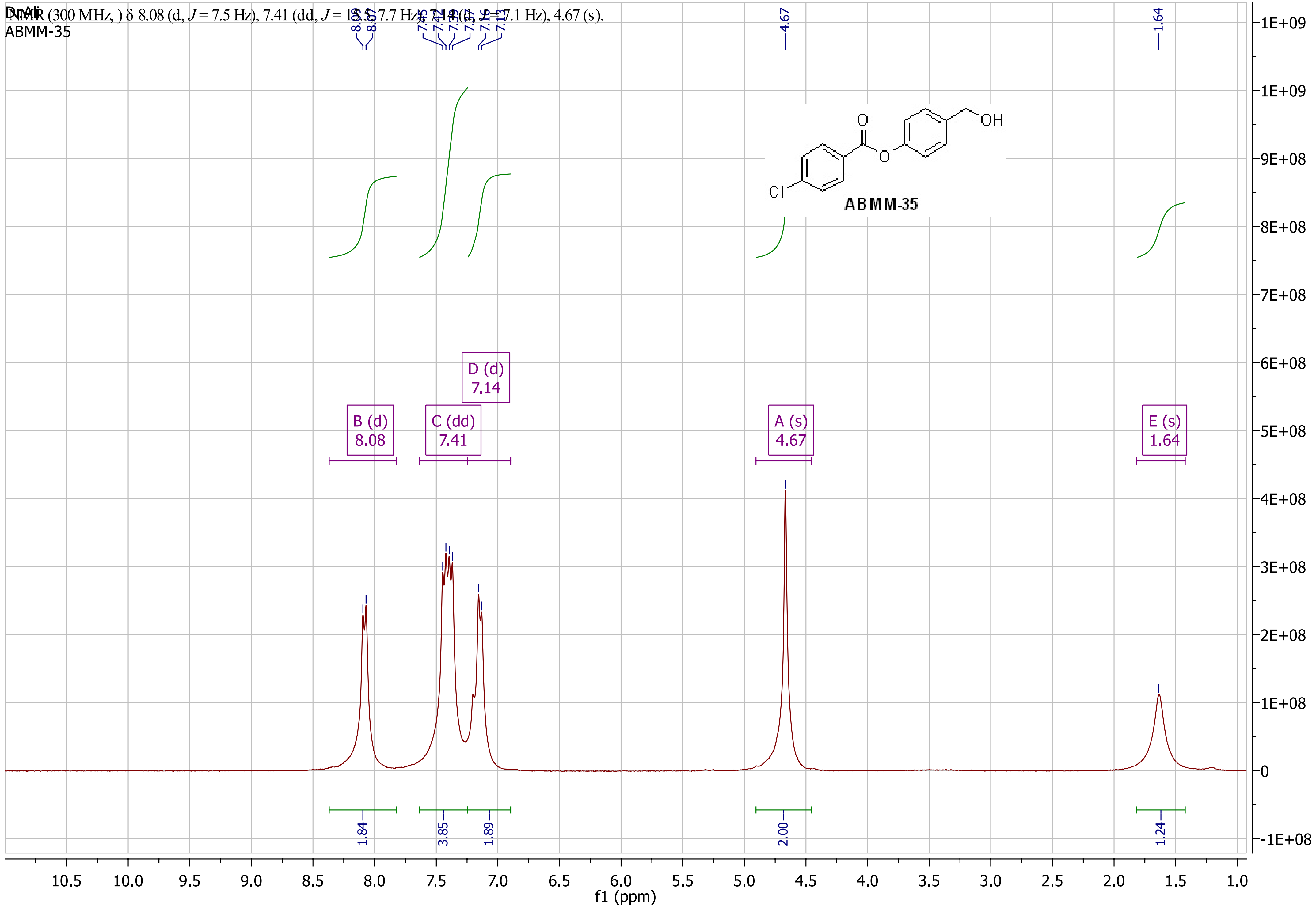

DrAli
ABMM-35
ABMM-35
B (d) 8.08
C (dd) 7.41
D (d) 7.14
A (s) 4.67
E (s) 1.64
1.84
3.85
1.89
2.00
1.24
f1 (ppm)

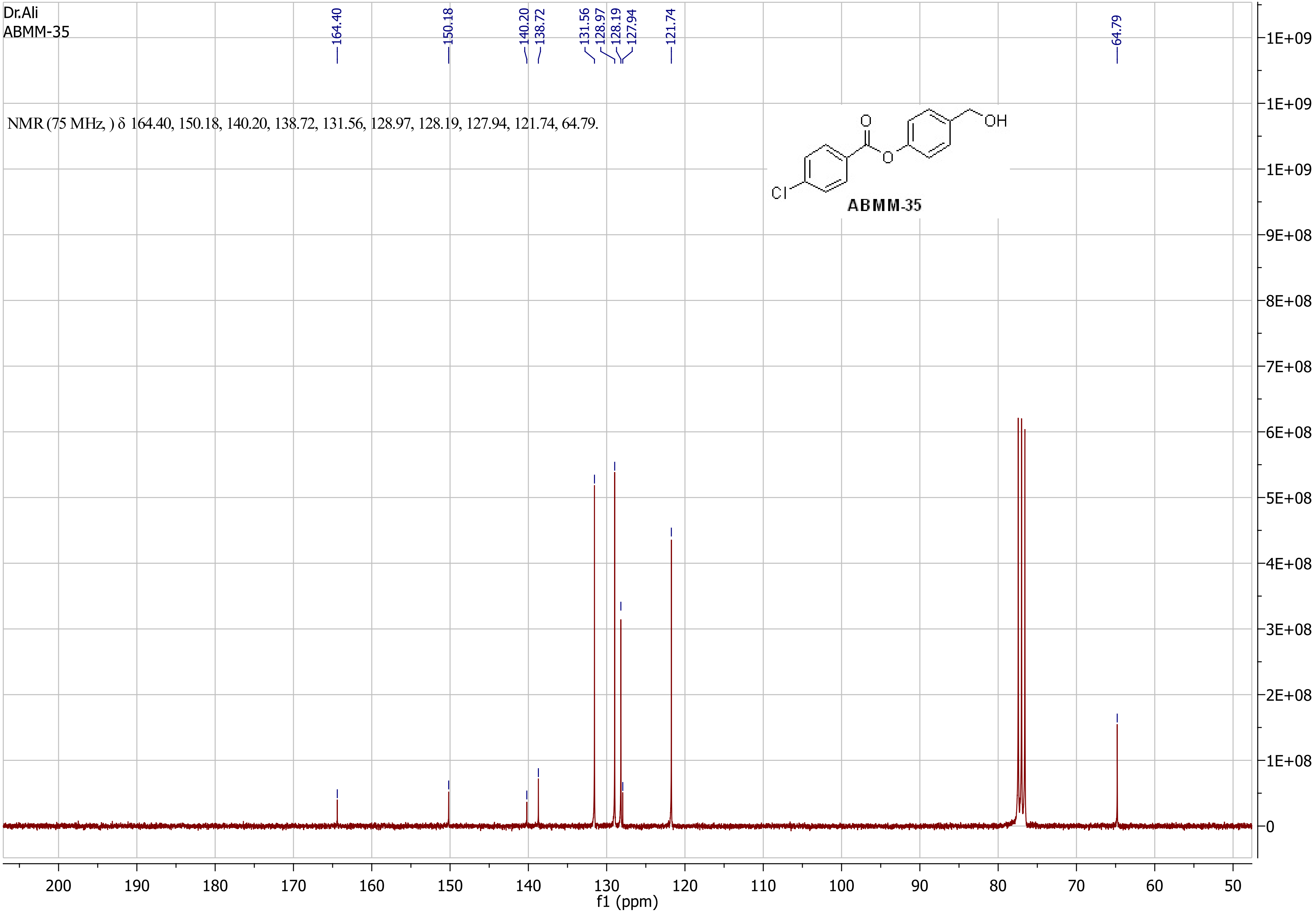
Dr.Ali
ABMM-35
164.40
150.18
140.20
138.72
131.56
128.97
128.19
127.94
121.74
64.79
NMR (75 MHz,) δ 164.40, 150.18, 140.20, 138.72, 131.56, 128.97, 128.19, 127.94, 121.74, 64.79.
O
O
OH
Cl
ABMM-35
1E+09
1E+09
1E+09
9E+08
8E+08
7E+08
6E+08
5E+08
4E+08
3E+08
2E+08
1E+08
0
200
190
180
170
160
150
140
130
120
110
100
90
80
70
60
50
f1 (ppm)
